# **α**-Diversity analysis of hepatic transcriptome reveals distinct pathways in alcohol-associated hepatitis

**DOI:** 10.1172/jci.insight.200727

**Published:** 2026-01-08

**Authors:** Sudrishti Chaudhary, Jia-Jun Liu, Silvia Liu, Marissa Di, Juliane I. Beier, Ramon Bataller, Josepmaria Argemi, Panayiotis V. Benos, Gavin E. Arteel

**Affiliations:** 1 Department of Medicine, Division of Gastroenterology, Hepatology and Nutrition,; 2 Pharmacology and Chemical Biology,; 3 Organ Pathobiology and Therapeutics Institute,; 4 Pittsburgh Liver Research Center, and; 5 Department of Computational and Systems Biology, University of Pittsburgh, Pittsburgh, Pennsylvania, USA.; 6 Institut d’Investigacions Biomediques August Pi i Sunyer (IDIBAPS), University of Barcelona, Barcelona, Spain.; 7 Department of Internal Medicine, Liver Unit, Clinical University of Navarra, Navarra, Spain.; 8 Department of Epidemiology, University of Florida, Gainesville, Florida, USA.

**Keywords:** Hepatology, Inflammation, Transcriptomics

## Abstract

Next-generation sequencing can identify previously uncharacterized gene expression patterns in disease. Beyond differentially expressed gene (DEG) analysis, we investigated the ability of within-population diversity (α-diversity) of the transcriptome to reveal additional biological information in alcohol-associated liver disease (ALD), comparing differential Shannon diversity (DSD) to transcriptome heterogeneity changes. RNA sequencing data from normal livers and patients with early ALD and severe AH were analyzed. α-Diversity indices and percentage Shannon diversity of a gene, which refers to this gene’s contribution to total Shannon entropy, were calculated. Ingenuity pathway analysis identified canonical pathways determined by DEG and DSD approaches. ALD significantly decreased hepatic transcriptome α-diversity, correlating with increased relative contribution of select genes. These changes were driven by lower-abundance gene expression loss. DEG and DSD analyses showed overlapping genes and canonical pathways, but DSD also identified additional genes and pathways not highlighted by DEGs, including fatty acid oxidation, extracellular matrix degradation, and cholesterol metabolism pathways that may represent additional therapeutic targets. Importantly, DSD more effectively identified differences between ASH and AH. Overall, α-diversity analysis revealed that ALD progressively reduces transcriptome heterogeneity, and that DSD provides complementary insights into disease mechanisms missed by standard approaches.

## Abstract 2

The work represents a notable methodological advancement that bridges ecological theory with clinical hepatology, offering new perspectives on disease progression and potentially informative biomarkers.

## Graphical abstract

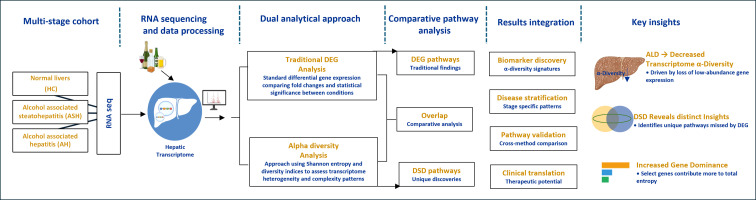

## Introduction

Alcohol-associated hepatitis (AH) is a subacute form of alcohol-associated liver disease (ALD), characterized by jaundice, ascites, and other sequelae associated with severe hepatic decompensation. AH has a high mortality rate of 30%–50% at 3 months and 40% at 6 months (1). Although the clinical progression of AH has been well described for decades, the underlying mechanisms that drive AH are incompletely understood. The only FDA-approved therapy for AH (corticosteroids) has been used since the 1950s, despite having only limited efficacy in patients with AH (2). Hence, the need arises for developing better therapeutic options, for which it is crucial to understand the underlying mechanisms of AH.

To address these gaps in understanding, the National Institute on Alcohol Abuse and Alcoholism (NIAAA) launched AlcHepNet in 2012 (3); this multicenter translational and clinical research program was designed to accelerate the understanding of AH. This program also led to the storage of large cohorts containing clinical data and biobanked samples, which have been available to researchers in the field for primary and secondary analyses. These studies and the establishment of clinical biobanks have paved the way for future research and development of new information and insights for AH. For example, multiplatform next-generation sequencing (NGS) of transcripts from liver biopsies highlighted expression changes in patients with AH that suggest a pseudofetal hepatic function (4).

NGS is a powerful tool for detecting gene expression patterns, also uncovering unrecognized or cryptic gene expressions in disease states (5). Differentially expressed gene (DEG) analysis has been established as a common genetic analysis method, focusing on genes that are significantly upregulated (expressed at higher levels) or downregulated (expressed at lower levels) under different conditions (6). These approaches have been useful in detecting targets or biomarkers and helping generate hypotheses and mechanisms behind diseases. However, DEG analysis assumes transcriptional independence, as it focuses on individual genes. For this reason, DEG analysis is often coupled with pattern recognition algorithms (e.g., Ingenuity Pathway Analysis; IPA) to identify changes in enrichment in specific biological pathways. However, pattern recognition approaches rely on curated knowledge, which is incomplete, and do not agnostically identify patterns de novo (7).

In addition to analysis at the individual gene level, transcriptomic data are often analyzed using algorithms developed from ecosystem diversity analyses. For example, β-diversity indices and multivariate analyses are often employed to describe how the overall transcriptome differs between 2 different states (e.g., diseased vs. healthy). β-Diversity compares the gene expression pattern between conditions and quantifies the similarity or dissimilarity of the different groups. For example, principal component analysis (PCA) clusters samples that have similar expression profiles (i.e., low β-diversity). These approaches build on the understanding that environmental stressors often impact the totality of gene expression and provide a more holistic view of comparative transcriptome dynamics. However, these approaches are used predominantly for visualizing the overall changes in gene expression and not for identifying specific mechanisms and pathways (8).

In contrast with between-sample diversity (i.e., β-diversity), within-sample diversity (i.e., α-diversity) is rarely used to analyze transcriptomic data sets. Moving away from the traditional DEG approach, this study employs indices of α-diversity and introduces an analysis of gene expression based on α-diversity (i.e., Differential Shannon Diversity; DSD). Unlike DEG analysis, which focuses on individual gene expression changes and can be sensitive to outliers, or indices of β-diversity, which measure variation between samples but may not pinpoint specific genes, α-diversity indices like Shannon diversity can provide a more holistic view of transcriptome complexity while still allowing for gene-level analysis. The DSD approach allows for the quantification of each gene’s contribution to overall transcriptome diversity, potentially revealing subtle but important changes that might be missed by traditional methods. The purpose of this study was therefore to determine the effect of AH on indices’ α-diversity of the hepatic transcriptome, as well as investigate the pattern of gene expression determined by DSD.

## Results

### ALD decreases the α-diversity of the hepatic transcriptome.

**Figure 1:**
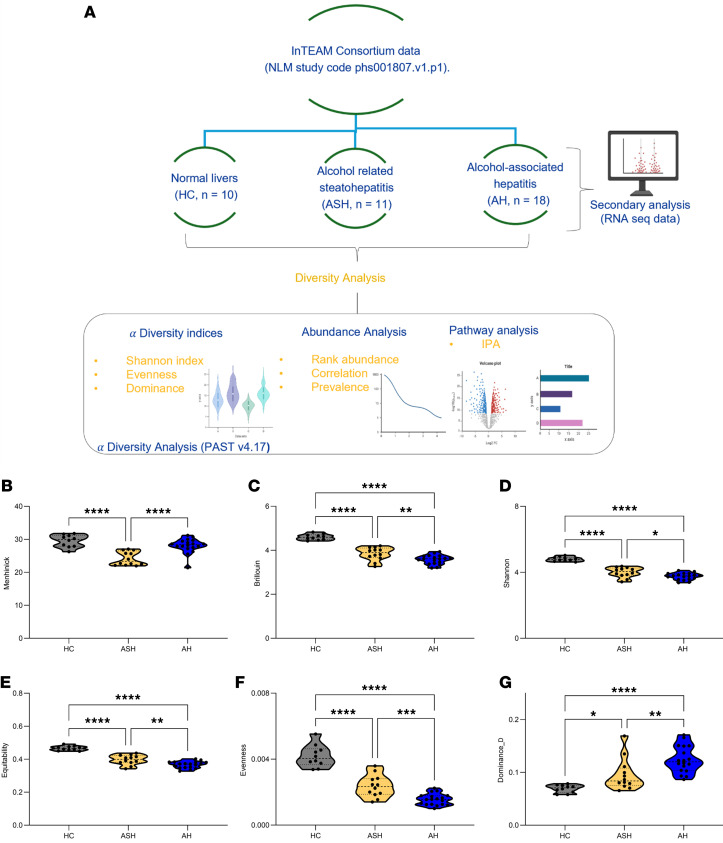
Study design and analytical workflow for transcriptome analysis of alcohol-associated liver disease progression. (**A**) Secondary analysis of RNA-seq data to characterize transcriptional changes during disease progression, examining gene expression across healthy controls (HC, *n* = 10), patients with alcohol-associated steatohepatitis (ASH, *n* = 11), and patients with alcohol-associated hepatitis (AH, *n* = 18). Analysis included α-diversity assessment using PAST v4.17 (Shannon index, evenness, dominance), abundance analysis with gene ranking and rank differences, and pathway analysis using IPA. (**B**–**G**) Indices of α-diversity. Violin plots showing the distribution of α-diversity as measured by (**B**) Menhinick, (**C**) Brillouin, (**D**) Shannon, (**E**) Equitability, (**F**) Evenness, and (**G**) Dominance indices for the 3 groups: HC, ASH, and AH. One-way ANOVA analysis with Tukey’s post hoc test revealed significant grouping between groups. *P < 0.05; **P < 0.01; ***P < 0.001; ****P < 0.0001.

Figure 1A outlines a transcriptome study from the InTEAM Consortium (NLM study code phs001807.v1.p1) comparing gene expression profiles across disease groups: healthy controls (HCs; 10 participants), patients with early silent ALD (ASH; 11 participants), and patients with AH (18 participants). We further performed secondary analysis, which specifically focused on RNA-seq data to understand how gene expression patterns change during ALD progression. α-Diversity analysis was performed to assess transcriptome diversity through Shannon index, evenness, and dominance metrics, along with abundance analysis that ranks genes from lowest to highest expression and calculates rank differences between groups. We also conducted pathway analysis using IPA to identify differentially regulated biological pathways across the progression, from healthy liver to AH. To evaluate potential differences in α-diversity profiles of individuals with the disease spectra (ASH, AH) and HCs, α-diversity was quantified by richness, evenness, dominance, and related indices (Figure 1, B–G).

The Menhinick index attempts to estimate species richness (i.e., number of unique species) independently of sample size. It is calculated here as the number of genes with non-zero expression in the sample divided by the square root of the sum of read counts of all genes in the sample. ASH significantly decreased the Menhinick index, compared with HC, whereas AH showed a partial recovery toward HC values (Figure 1B). This pattern suggests that while early ALD (ASH) is characterized by a loss of detectable transcripts, severe disease (AH) may involve reexpression of genes not typically expressed in healthy liver, potentially including aberrant or fetal transcripts, even as overall diversity continues to decline by other measures. The Brillouin (Figure 1C) and Shannon (Figure 1D) diversity indices showed a stepwise reduction from HC to ASH to AH. Indices of Equitability (Figure 1E) and Evenness (Figure 1F) followed a similar trend, indicating that the distribution of level of gene expression became less uniform with ALD disease severity. The Dominance_D index, which quantifies the extent to which expression is concentrated in a few highly expressed genes rather than distributed evenly across the transcriptome (Figure 1G), showed an inverse pattern, with AH having the highest value. This increased dominance in AH indicates that a smaller subset of genes accounts for a disproportionately large share of total transcription in severe disease. Taken together, these results collectively suggest a progressive decrease in hepatic gene expression diversity and evenness, coupled with an increase in dominance, as liver disease severity progresses from healthy to ASH to AH.

### Diversity and changes by abundance.

**Figure 2:**
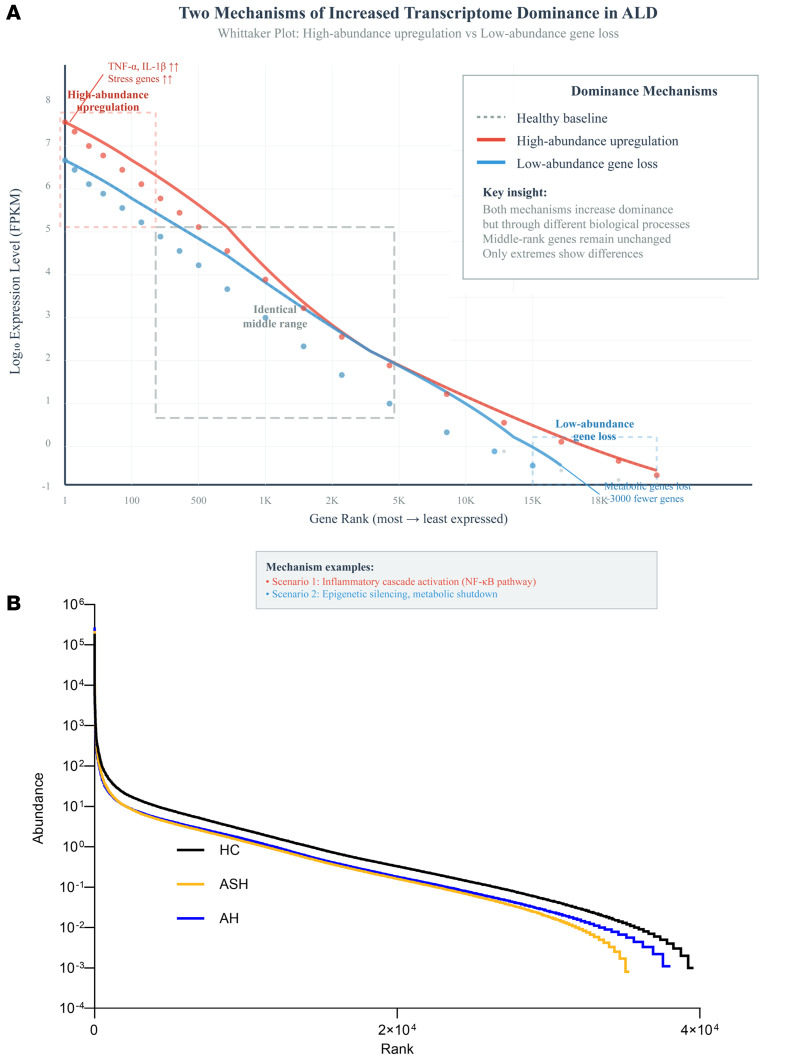
Diversity and changes by abundance. (**A**) Conceptual Whittaker plot illustrating 2 mechanisms that could increase transcriptome dominance in ALD: either upregulation of high-abundance genes (red) or loss of low-abundance genes (blue), both deviating from the healthy baseline (gray dashed line). Mid-ranked genes remain relatively stable. Red and blue boxes highlight distinct biological processes with different therapeutic implications. (**B**) Whittaker plot showing changes by abundance and prevalence rank. Relative abundance-rank curve of α-diversity in different groups based on average of each gene per group.

The above-described changes to indices of diversity indices (Figure 1, B–G) can be generally described by either an increase in the relative expression of highly abundant genes or via a loss of expression in low-abundance genes. Figure 2A demonstrates 2 potential mechanisms by which ALD could cause increased transcriptome dominance. The red curve shows high-abundance gene upregulation, where already highly expressed genes are further amplified through inflammatory cascades, effectively concentrating transcriptional resources among dominant genes. The blue curve illustrates low-abundance gene loss, where weakly expressed genes are systematically silenced through epigenetic suppression or metabolic shutdown, reducing the total transcriptome diversity. Importantly, middle-ranked genes remain unchanged in both scenarios, indicating that dominance increases through changes at expression extremes rather than global shifts. This reveals that disease-associated transcriptome dominance can result from either amplifying dominant pathways or eliminating minor contributors, with significant implications for targeted therapeutic strategies. Figure 2B provides a comprehensive analysis of gene expression as a factor of expression abundance to visualize these potential mechanisms. It shows the pattern of abundance as a function of prevalence rank (Whittaker plot). This plot shows a similar overall pattern of gene expression between the groups, with subtle differences apparent only in the lower-abundance genes as shown in the schematic representation in Figure 2A.

### Pathway analysis of significantly changed genes determined by DEG and DSD analyses.

**Figure 3:**
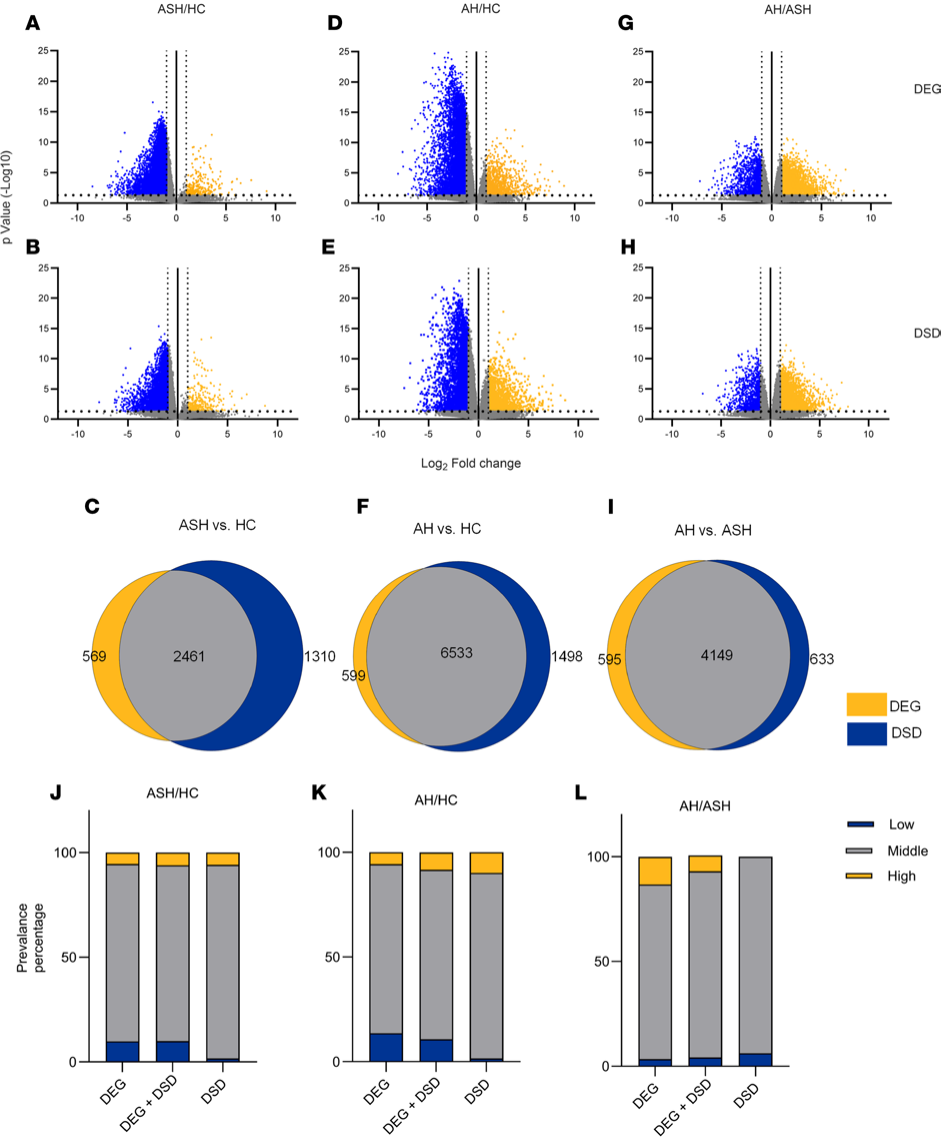
DEG versus DSD: shared and unique changes. Volcano plot representation of differentially expressed genes (DEGs) and the Differential Shannon Diversity (DSD) data sets in ASH versus HC (**A** and **B**), AH versus HC (**D** and **E**), and AH versus ASH (**G** and **H**). The yellow and blue points indicate increased and decreased gene expression, respectively. The *x*-axis shows log_2_(fold-change) and the *y*-axis the –log_10_(*P* value) (*P* ≥ 1.3) of the gene expression. Venn diagrams depicting unique and common subsets of genes shared by both DEGs and DSD approaches in (**C**) ASH versus HC, (**F**) AH versus HC, and (**I**) ASH versus AH groups. Bar diagrams (**J**–**L**) depicting percentage of low-, medium-, and high-abundance genes across the groups, common and unique to DEG and DSD.

The above-described analyses (Figure 1) indicate an interesting effect of ALD disease severity on indices of transcription diversity, indicating a stepwise deregulation of lower-abundance genes in ASH and AH. However, these analyses describe global changes in the transcriptome and do not highlight specific changes in genes. The effect of ALD disease severity on changes in gene expression was therefore determined using DSD (see Methods for details) and compared with traditional DEG analysis. The volcano plots, which show –log_10_(*P* value) as a function of log_2_(fold change) (log_2_FC), and Venn diagrams of the DEG and the DSD data sets are presented in Figure 3, A–I). DEG and DSD analyses yielded quantitatively similar results, as depicted by volcano plots for ASH versus HC (Figure 3, A and B), AH versus HC (Figure 3, D and E), and AH versus ASH (Figure 3, G and H). Figure 3, C, F, and I depict Venn diagrams of genes identified by DEG and/or DSD that were significantly upregulated or downregulated and were common or unique to both the approaches. Venn diagrams for each of the diseases ASH/HC (Figure 3C), AH/HC (Figure 3F), and AH/ASH (Figure 3I) depict significant overlap between genes chosen by both DEG and DSD techniques. Moreover, there were genes uniquely identified by both analytical approaches. For example, both DEG and DSD analyses identified more unique genes in the AH versus ASH group compared with other groups with respect to HCs; thus, the uniquely identified genes were more balanced between the approaches in the AH versus ASH group. The above results revealed an overall pattern of gene expression between the groups, with subtle differences apparent only in the lower-abundance genes. The prevalence percentage was calculated for the unique as well as common set of genes derived from the Venn analysis in all the disease states (Figure 3, J–L), which also supported the above results that show that the high-prevalence genes were generally less impacted by both disease states, and the medium- and lower-abundance genes show higher variability in response to ASH and AH. The percentage coefficient variation (CV), calculated by the standard deviation divided by the mean, showed no difference between DEG and DSD analysis in gene expression across the groups (Supplemental Figure 1; supplemental material available online with this article; https://doi.org/10.1172/jci.insight.200727DS1).

**Figure 4:**
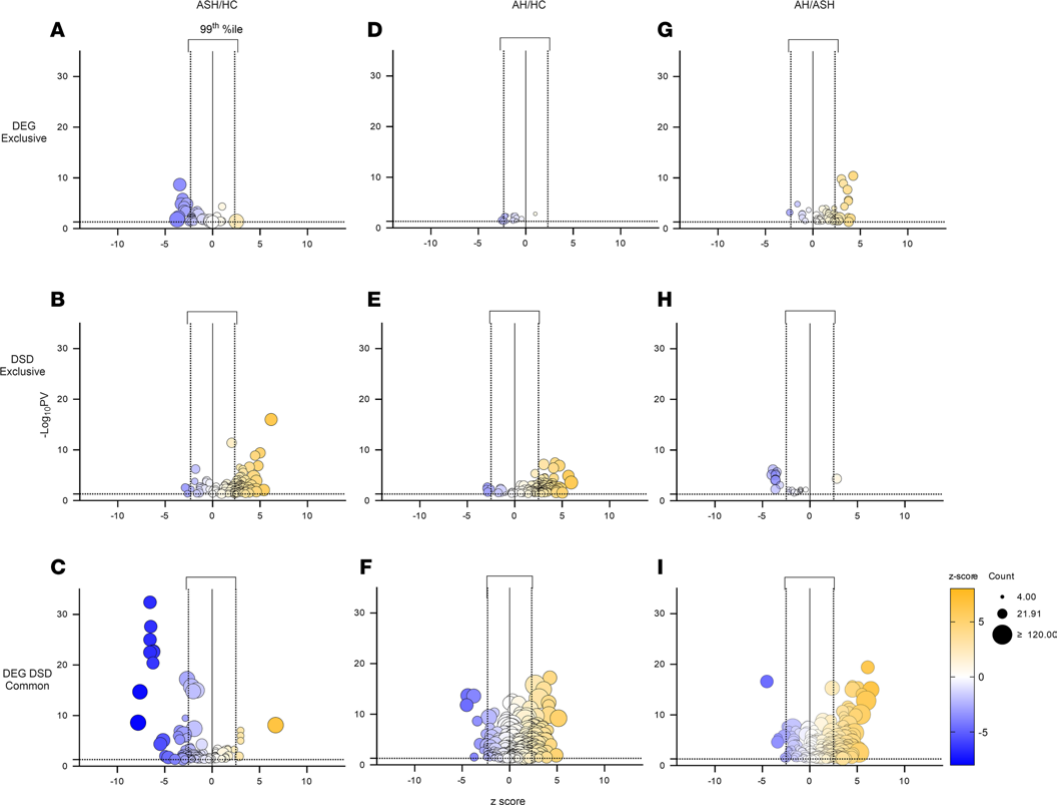
Pathway enrichment analysis of DEG and DSD. Bubble plot showing significantly enriched pathways both common and exclusive for the differentially expressed genes (DEGs) and percentage Shannon diversity (DSD) in disease groups ASH versus HC (**A**–**C**) and AH versus HC (**D**–**F**) and AH versus ASH (**G**–**I**). Size of the bubbles is proportional to the gene count. The *y*-axis represents the negative logarithm of the log_10_(*P* value) for the genes, and the *x*-axis displays the *z* score. The threshold for displaying the bubble labels was set to a *z* score of 2.3. Bubbles for genes belonging to a high *z* score are depicted in yellow and a low *z* score in blue.

Figure 4, A–I, shows a modified volcano plot depicting the results of Ingenuity Pathway Analysis (IPA) of significantly changed genes determined by DEG and DSD in the various comparisons where the enriched Gene Ontology (GO) terms (–log_10_[*P* value]) were plotted as a function of the *z* score. These bubble plots showed significantly enriched pathways both common and exclusive for the DEGs and DSDs in disease groups ASH, AH versus HC, and AH versus ASH. IPA identified both overlapping and unique canonical pathways by both analyses in each of the groups (Supplemental Tables 2–10). The DSD analysis identified additional genes and pathways not highlighted by the DEG approach, specifically in the AH versus ASH group. Some of these pathways have been established to potentially contribute to ALD, e.g., fatty acid oxidation (GO: 0019395, *P* = 1.34 × 10^–5^; Supplemental Tables 8–10).

**Figure 5:**
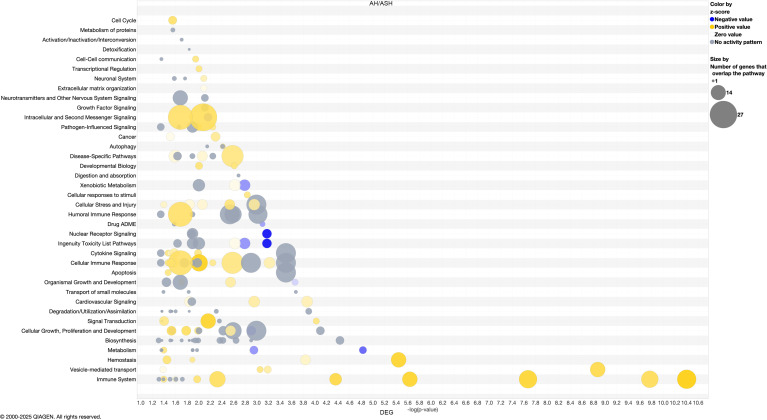
Canonical pathways for DEG exclusive genes. Canonical Pathway scores plotted as pathway category based on gene expression level for the DEG exclusive pathways in the AH versus ASH group. The log_10_(*P* value) for each pathway is plotted on the *x*-axis versus the pathway categories plotted along the *y*-axis. By default, the coloring relates to the pathway’s *z* score, and the bubble size relates to the number of dataset genes that overlap each pathway, as shown in the legend.

**Figure 6:**
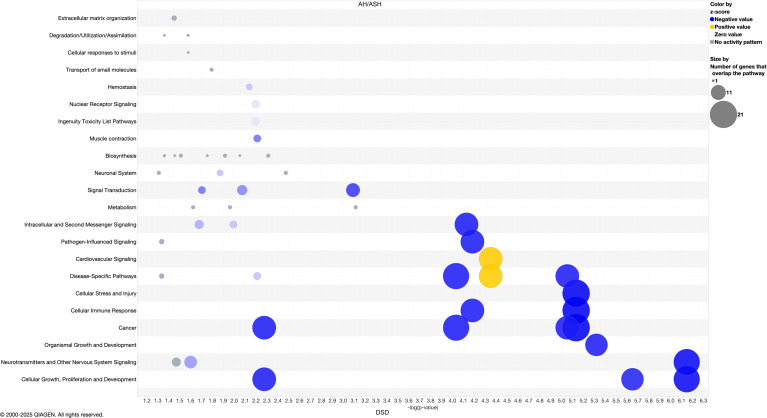
Canonical pathways for DSD exclusive genes. Canonical pathway scores plotted as pathway category based on gene expression level for the DEG exclusive pathways in the AH versus ASH group. The log_10_(*P* value) for each pathway is plotted on the *x*-axis versus the pathway categories plotted along the *y*-axis. By default, the coloring relates to the pathway’s *z* score, and the bubble size relates to the number of dataset genes that overlap each pathway, as shown in the legend.

**Figure 7:**
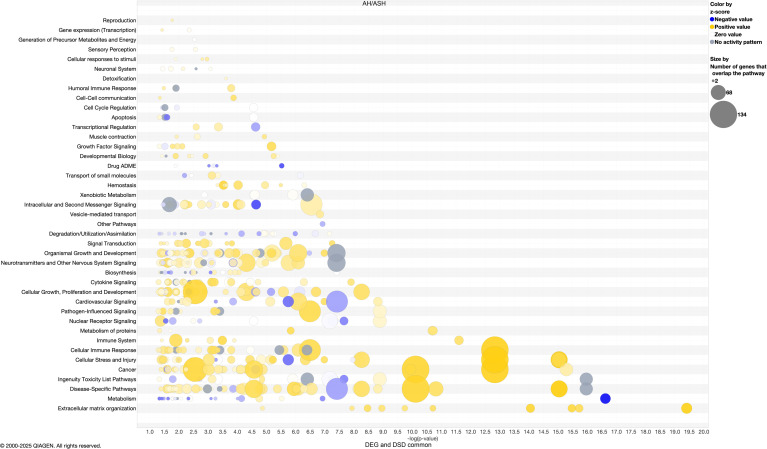
Canonical pathways for DEG and DSD common genes. Canonical pathway scores plotted as pathway category based on gene expression level for the common pathways in DEG and DSD for the AH versus ASH group. The log_10_(*P* value) for each pathway is plotted on the *x*-axis versus the pathway categories plotted along the *y*-axis. By default, the coloring relates to the pathway’s *z* score, and the bubble size relates to the number of dataset genes that overlap each pathway, as shown in the legend.

IPA of significantly changed genes determined by DSD analysis identified differences between less severe ALD (ASH) and more severe disease state (AH) more effectively than DEG analysis. The pathways from their scores clustered into categories were identified. Canonical pathway scores plotted as pathway category based on gene expression level for the DEG exclusive, DSD exclusive, and the DEG and DSD common genes in the AH versus ASH group is presented in Figures 5,6,7. (The other groups’ data are presented in Supplemental Figures 2 and 3). The top genes identified by DEG did not include a significant number of disease-specific pathways, in contrast with the genes identified by DSD. The enrichment analysis using the DSD approach revealed pathways most significantly enriched in categories related to degradation of extracellular matrix, inhibition of matrix metalloproteases, cellular stress and injury, apoptosis, and cellular immune response (Figure 6).

### α-Diversity analysis of hepatic transcriptome in ALD.

**Figure 8:**
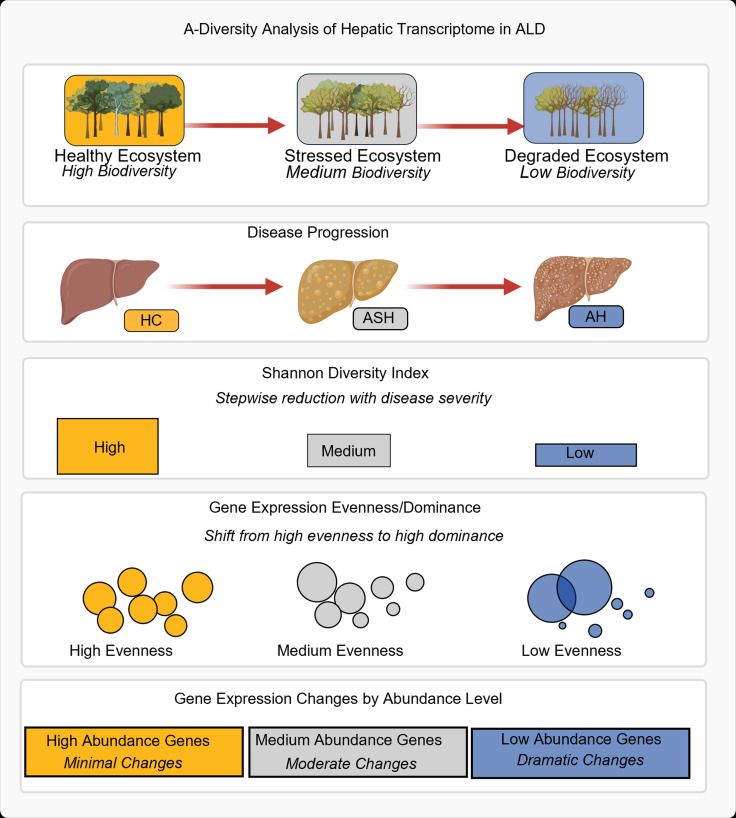
Conceptual framework for α-diversity analysis of hepatic transcriptome in alcohol-associated liver disease progression. (**A**) Schematic illustration of transcriptome diversity changes during liver disease progression using ecological diversity principles. Ecosystem analogy shows transition from healthy (high biodiversity) to stressed (medium biodiversity) to degraded (low biodiversity) states. Corresponding liver disease progression from healthy controls (HCs) through early silent alcohol-associated steatohepatitis (ASH) to alcohol-associated hepatitis (AH). Shannon diversity index demonstrates stepwise reduction in transcriptome diversity with increasing disease severity. Gene expression evenness and dominance patterns shift from high evenness (many genes with similar expression levels) to high dominance (few highly expressed genes). Abundance-dependent gene expression changes showing minimal alterations in high-abundance genes, moderate changes in medium-abundance genes, and dramatic changes in low-abundance genes during disease progression.

Figure 8 illustrates the application of ecological diversity concepts to analyze hepatic gene expression changes across the spectrum of ALD. An ecological framework demonstrates parallel patterns of biodiversity loss between natural ecosystems and liver transcriptomes. Environmental stressors drive ecosystem degradation from diverse communities (Figure 8, left) to homogeneous landscapes with reduced species diversity (Figure 8, right), mirroring transcriptome changes in disease progression. Disease progression from HC (green) to ASH (yellow) to AH (red) involves progressive changes in liver architecture, with increasing lipid accumulation and disruption of normal hepatic structure. The Shannon diversity index, a measure of transcriptome heterogeneity, shows stepwise reduction with disease severity, from high in HC to medium in ASH to low in AH. Gene expression evenness/dominance patterns illustrate how transcriptome composition shifts from high evenness (multiple similarly expressed genes in HC) to low evenness/high dominance (few genes dominating expression in AH). Disease-induced transcriptome changes disproportionately affect genes based on their abundance levels, with low-abundance genes showing the most dramatic expression changes and high-abundance genes remain relatively stable. This ecological perspective demonstrates how chronic alcohol exposure reduces transcriptome diversity in the liver, similar to how environmental stress reduces biodiversity in natural ecosystems.

### Cellular composition changes across ALD disease progression.

**Figure 9:**
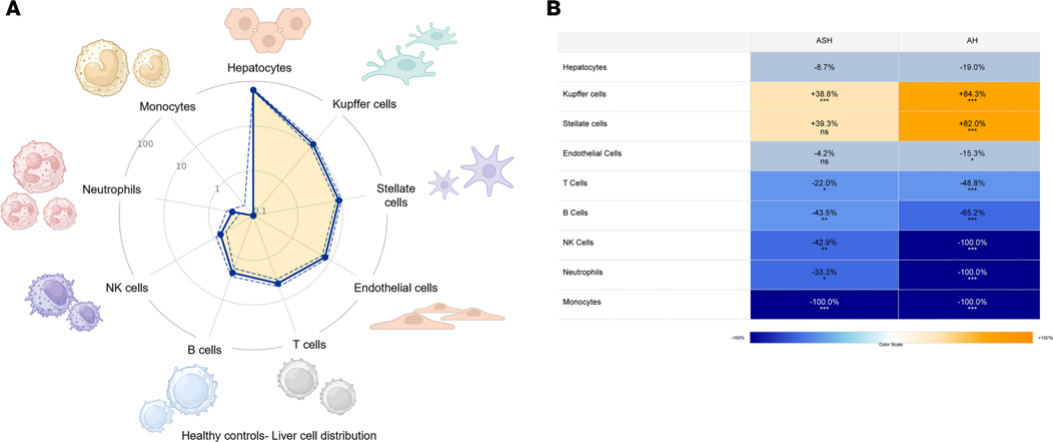
Liver cell type distribution. (**A**) Radar plot displaying liver cell type distribution in healthy controls (*n* = 10) on log_10_ scale. Data points show mean values positioned on radial axes, with error bars representing ±1 SEM. Dashed lines indicate upper and lower confidence boundaries (mean ± SEM). Vector length is proportional to log_10_(cell count), effectively visualizing the 643-fold dynamic range from 0.1 (monocytes) to 64.3 (hepatocytes) cells per field. Gold circle around monocytes indicates high measurement uncertainty (±100% CV). (**B**) Heatmap showing percentage change from healthy controls across disease conditions with integrated statistical significance indicators. ASH (*n* = 12) and AH (*n* = 18) columns display changes for each cell type. Complete cell loss represented as –100% change in dark blue. White indicates minimal change (~0%). Statistical significance displayed below each percentage value: ***P < 0.001, **P < 0.01, *P < 0.05 by Welch’s *t* test versus healthy controls. NS, not significant.

Cellular deconvolution analysis using 528 validated marker genes across 9 major liver cell types was performed to estimate cellular composition (Figure 9, A and B). ALD caused progressive changes in cellular composition across the disease spectrum. Hepatocyte proportions decreased stepwise: HC (64.3% ± 3.2%) → ASH (58.7% ± 4.1%) → AH (52.1% ± 5.8%) (*P* < 0.001), representing a 19% total reduction from healthy to severe disease consistent with established ALD progression (9, 10). Kupffer cell (and macrophage) proportions increased across disease stages: HC (12.1% ± 1.8%) → ASH (16.8% ± 2.3%) → AH (22.3% ± 3.1%) (*P* < 0.001), representing an 84% increase in severe disease. This likely reflects infiltration of monocyte-derived macrophages rather than expansion of resident Kupffer cells, consistent with single-cell RNA-seq (scRNA-seq) studies demonstrating macrophage activation in ALD (11, 12). Stellate cell activation occurred progressively: HC (8.9% ± 1.3%) → ASH (12.4% ± 1.8%) → AH (16.2% ± 2.4%) (*P* < 0.001), likely reflecting the fibrotic response characteristic of advanced ALD, while endothelial cell proportions showed modest decreases with disease progression: HC (7.2% ± 1.1%) → ASH (6.9% ± 1.0%) → AH (6.1% ± 1.2%).

Estimated proportions of lymphocyte populations showed apparent decreases with disease progression. T cell proportions declined from 4.1% ± 0.8% in controls to 2.4% ± 0.6% in severe disease (*P* < 0.01), although recent work has identified patient heterogeneity, with a subset of patients with AH exhibiting elevated hepatic CD8^+^ T cells (13). B cells decreased from 2.3% ± 0.5% to 0.8% ± 0.2% (*P* < 0.001), and NK cells showed an apparent 86% reduction from 0.7% ± 0.2% to 0.1% ± 0.1% (*P* < 0.001). Deconvolution estimates for neutrophils and monocytes suggested low abundance across all groups (<0.5%); however, this finding contradicts the well-established neutrophilic infiltration characteristic of AH (14, 15). This discrepancy likely reflects phenotypic changes in disease-associated neutrophils, including the emergence of low-density neutrophils and myeloid-derived suppressor cells that exhibit reduced expression of canonical neutrophil markers such as CD16 (16, 17). Similarly, the apparent NK cell depletion may partly reflect phenotypic drift, as alcohol exposure alters NK cell maturation and receptor expression (18, 19).

These cellular composition changes occurred in parallel with the transcriptome diversity reduction described in the α-diversity analysis. The observed patterns of hepatocyte loss, macrophage infiltration, and altered immune cell signatures are consistent with findings from recent human scRNA-seq studies in ALD (10–12), although marker-based deconvolution approaches have inherent limitations in detecting cells with disease-altered phenotypes.

## Discussion

Our study explored whether ecological α-diversity metrics could provide additional insights into analyzing differential gene expression in transcriptomic data. Our work suggests that chronic alcohol exposure decreases overall transcriptome diversity in the liver, inducing various stress response pathways. While diversity is a fundamental concept in community ecology (20–22), its application to transcriptomics remains relatively unexplored. Ecological diversity measures have long been used to quantify species distribution patterns, with α-diversity defined as local diversity within a single area or ecosystem and β-diversity describing changes in species composition across areas (20). We hypothesized that applying these established ecological frameworks to transcriptomic data — conceptualizing the transcriptome as an ecosystem and individual genes as species — could reveal patterns and mechanisms missed by traditional analytical approaches. This ecological perspective allows us to investigate how environmental stressors, such as alcohol consumption, might affect the overall “ecosystem health” of the transcriptome, potentially disrupting expression patterns in ways analogous to how environmental stresses impact natural ecosystems. While this ecological lens has been applied in microbial community analysis (23, 24), its application to human transcriptomics represents a cross-disciplinary approach that may provide deeper insights into disease pathophysiology and progression, particularly in complex conditions like ALD.

β-Diversity measures, particularly through PCA, have been extensively used in transcriptomics to examine differences between populations or sample groups (24–26). These approaches help visualize variation across different conditions and identify major sources of transcriptome heterogeneity. β-Diversity metrics have proven particularly valuable in RNA-seq studies for detecting global patterns of gene expression changes across experimental conditions, disease states, and tissue types (6, 11, 12, 27). Researchers have employed distance matrices and ordination techniques like PCA, non-metric multidimensional scaling (NMDS), and t-SNE to relate gene expression patterns to phenotypic variables, treatment effects, and developmental stages (9, 11, 28). In contrast, α-diversity metrics in transcriptomics have typically been limited to describing and quantitating within-group variability (29), serving primarily as quality control measures or indicators of overall transcriptome complexity. Few studies have explored their potential for between-group comparisons in disease states and progression monitoring, despite their established utility in ecology for measuring ecosystem health and stability. This gap represents a significant opportunity to develop new analytical frameworks that could provide complementary insights into traditional differential expression analyses.

Analyzing various α-diversity indices across HC, ASH, and AH samples revealed a clear pattern: highest diversity in HC, followed by ASH, with lowest in AH, confirming that ALD decreases hepatic transcriptome diversity. We systematically evaluated multiple ecological diversity metrics, including Menhinick richness index, Shannon entropy, Brillouin index, and dominance measures to quantify transcriptome heterogeneity across disease states. Our analysis demonstrated a stepwise reduction in diversity metrics that correlated with disease severity, suggesting a progressive disruption of normal gene expression patterns with advancing ALD. Whittaker plots and log_2_FC scatter plots demonstrated that lower-abundance genes show more pronounced expression changes under disease conditions, consistent with our gene prevalence analysis. This finding is particularly significant, as it indicates that disease-related transcriptional changes disproportionately affect genes expressed at lower levels, potentially explaining why traditional differential expression approaches — which often favor highly expressed genes — might miss important pathological mechanisms. The pattern we observed mirrors ecological systems under stress, where rare species often exhibit greater sensitivity to environmental perturbations than dominant ones (30–32).

The healthy liver presents a rich, diverse transcriptome that chronic alcohol exposure disrupts, similar to how environmental stress decreases genetic diversity in natural populations. Chronic alcohol consumption creates a cellular environment characterized by oxidative stress, inflammation, and metabolic dysregulation that places “selective pressure” on gene expression patterns. Several recent studies have examined alcohol-induced transcriptomic changes using RNA-seq (6), multiomics approaches (12), and single-cell transcriptome research (10, 11), but few have applied diversity metrics as analytical tools. RNA-seq studies in murine models exposed to both chronic and binge ethanol feeding have documented widespread changes in gene networks governing lipid metabolism, inflammation, and cellular stress responses, paralleling many of the pathways we identified through our diversity-based approach. Comprehensive omics studies (28) and research on transcriptional dynamics (33) have further expanded our understanding, with recent scRNA-seq studies mapping liver cell heterogeneity in alcohol models and revealing cell-type-specific vulnerability to alcohol toxicity. This growing body of literature confirms alcohol’s profound effects on hepatic gene expression but has largely overlooked the ecological perspective of transcriptome diversity that our study introduces.

This study developed a method to analyze gene expression data called Differential Shannon Diversity (DSD). DSD relies on the Shannon diversity index, capable of measuring both richness and evenness of gene abundance for analyzing expression changes. When compared with DEG analysis, both methods generated overlaps and differences in gene sets. To look macroscopically, the patterns between DEG and DSD were very similar, implying that most of the genes were following the same pattern, but important unique changes were revealed through deeper analysis. Looking at the Venn diagrams (Figure 3, C, F, and I), while most of the genes followed the same pattern, there were genes uniquely identified by DEG analysis, and notably, genes that were uniquely identified by DSD. Interestingly, our analysis revealed that while gene richness (number of different transcripts) initially decreased in ASH compared with HCs, it increased again in AH, yet Shannon diversity continued to decline. This suggests that AH is characterized by the emergence of more transcript types (possibly including low-expressed fetal transcripts) but with decreased evenness due to dramatic expression changes in a subset of transcripts responding to liver failure. The DSD technique helped identify genes not found by DEG analysis, with each metric independently finding disease-relevant genes, such as those encoding the MITF/p300/CBP complex (*P* = 3.48 × 10^–4^), a CREB-binding protein that acts as a transcription regulator, and SPRING1 (*P* = 6.89 × 10^–5^), which is involved in SREBP signaling and cholesterol metabolism. Markedly, as compared with DEG, DSD proved to be a more sensitive method in detecting changes in highly expressed genes, even with small fold changes. Unlike DEG analysis, DSD was capable of detecting shifts in gene proportions, even when the absolute gene expression levels did not change.

Our study thus suggests that prolonged alcohol exposure reduces the variety of RNA transcripts in liver tissue while activating multiple stress response mechanisms. Integrating diversity methodologies through DSD reveals enriched pathways not highlighted by standard approaches. Looking forward, changes in transcriptome diversity could potentially serve as biomarkers for ALD progression or treatment response, although future research should determine whether these findings are specific to AH or generalized to all exogenous stresses.

## Methods

### Sex as a biological variable.

This study performed secondary analysis on publicly available RNA-seq data with both male and female patients. Sex was not considered as a biological variable in the study.

### Publicly available liver RNA-seq analysis.

RNA-seq data were obtained from normal livers (*n* = 10) and from biopsies of patients with ASH (*n* = 11), non-severe AH (*n* = 9), and severe AH (*n* = 9) from the InTEAM Consortium – Alcohol-associated Hepatitis Liver RNA Sequencing study, sponsored by the NIAAA. The study details and sequencing data can be found in the Database of Genotypes and Phenotypes (dbGAP, phs001807.v1.p1) of the NIH. The basic clinical and laboratory data of the patients included in this study, the methods used to extract RNA and perform deep RNA-seq, and the bioinformatic pipelines used to determine transcript counts have been described previously (9). Indices of α-diversity (e.g., Shannon index, evenness, and dominance) were calculated using PAST (v. 4.17) software (https://www.nhm.uio.no/english/research/resources/past/). The abundance rank correlation coefficient was calculated, each data set was ranked from lowest to highest, and then rank differences and square of each of those differences were calculated, followed by the calculation of the sum of all the squared differences. This was calculated as a Spearman rank correlation function in Microsoft Excel.

### DEG and DSD analyses.

The impact of ALD and AH on gene expression was measured by the log_2_FC between groups. We tested 2 differential metrics: one based on the DEGs and the other on the normalized DSD. The probability of observing gene *i* in this sample (*p_i_*) was calculated as detailed in Equation 1.


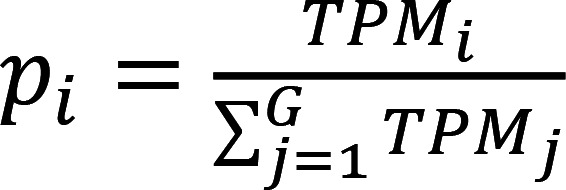


Where TPM represents transcripts per million, and *G* is defined as the total number of expressed genes for a given sample.

Next, the log_2_-based Shannon entropy (*H*) was used to measure the RNA diversity for a given library, which is calculated as detailed in Equation 2.


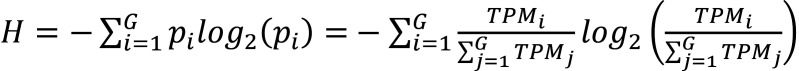


Here, *H* ranges from 0 to log_2_ (*G*). If *H* = 0, then only 1 gene is expressed for that library. And if *H* = log_2_(*G*), then all the genes are evenly expressed.

In order to quantify the expression weight of a gene among all the genes of a given sample, the percentage Shannon entropy (PSE) was defined to illustrate the percentage of gene *i* in terms of Shannon entropy (*H*). This measure represents the contribution of each individual gene to the overall Shannon entropy of the transcriptome, normalized by the total entropy. This is mathematically represented by Equation 3.


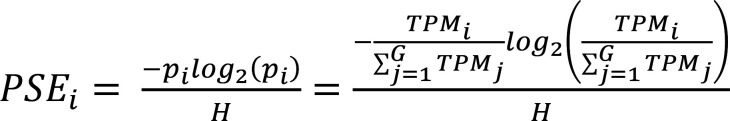


PSE values range from 0 to 1, where 0 indicates that gene is not expressed and PSE will be close to 1 if only gene (*i*) is expressed for a given sample.

Based on PSE per gene, the DSD was calculated as the log_2_FC of PSE values in the case sample over PSE value in the control sample. Thereby, it allows DSD to be directly compared to DEG values, which is defined by the log_2_FC of the gene expression intensity. Finally, the PSE was calculated for all the genes across all the samples. Per gene, the average PSE was calculated across the RNA libraries with the same condition. Wilcox’s tests were performed for pairwise conditions.

### Cellular deconvolution analysis.

Liver cellular composition was estimated using a reference-based deconvolution approach with 528 validated marker genes across 9 major liver cell types (hepatocytes, Kupffer cells, stellate cells, endothelial cells, T cells, B cells, NK cells, neutrophils, and monocytes; Supplemental Table 1). Cell type proportions were calculated for each sample and expressed as percentages of total liver cell composition. Statistical comparisons across disease groups were performed using 1-way ANOVA followed by post hoc analysis, with significance set at *P* less than 0.05.

### Bioinformatics pathway analyses.

Pathway analyses were conducted using IPA software (QIAGEN). This software was utilized for canonical pathway analysis and network discovery. IPA’s core analyses rely on existing knowledge of the relationships between upstream regulators and their downstream target genes, which are stored in the Ingenuity Knowledge Base. Fisher’s exact test was employed to calculate *P* values for the analysis. IPA was used to identify top canonical and enriched biological pathways determined by DEG and DSD approaches. The cutoff for log_2_FC values was 1 or greater and –1 or less for the upregulated and downregulated genes, respectively. A log_10_(*P* value) of 1.3 or greater was selected for the study as the level of significance.

### Statistics.

For the DEG and DSD analyses, Wilcox’s tests were performed. Spearman’s rank correlation test was employed to assess correlations between gene expression and prevalence rank. All statistical analyses were conducted and graphs were generated using Prism software from GraphPad.

### Study approval.

This study involved secondary analysis of existing sequencing data obtained from the Database of Genotypes and Phenotypes (dbGAP, phs001807.v1.p1) maintained by the NIH. As this research utilized only deidentified, publicly available data, institutional review board (IRB) approval was not required. The original studies contributing data to dbGAP received appropriate IRB approval from their respective institutions prior to data deposition.

### Data availability.

Data are available from the Database of Genotypes and Phenotypes (dbGaP, accession phs001807.v1.p1). Data from current study are available in the Supporting Data Values file.

## Author contributions

SC conceptualized the study, developed methodology, analyzed data, conducted experiments, generated figures, and contributed to the original draft of the manuscript. JJL analyzed data, contributed software, and wrote, reviewed, and edited the manuscript. SL analyzed data, contributed software, provided supervision, and wrote, reviewed, and edited the manuscript. MD analyzed data and contributed software. JIB wrote, reviewed, and edited the manuscript. RB provided resources, data curation, and acquired funding, and reviewed and edited the manuscript. JA provided resources, data curation, and reviewed and edited the manuscript. PVB analyzed data, contributed software, developed methodology, and reviewed and edited the manuscript. GEA conceptualized the study, provided supervision and project administration, acquired funding, and wrote, reviewed, and edited the manuscript.

## Funding support

This work is the result of NIH funding, in whole or in part, and is subject to the NIH Public Access Policy. Through acceptance of this federal funding, the NIH has been given a right to make the work publicly available in PubMed Central.

- National Institute of Diabetes and Digestive and Kidney Diseases (NIDDK)/NIH grant R01 DK130294.
- NIAA/NIH grant R01 AA028436.
- NIDDK/NIH Center Core grant P30 DK120531 (to the Pittsburgh Liver Research Center at the University of Pittsburgh).
- National Heart, Lung, and Blood Institute/NIH grant R01 HL159805.

## Supplementary Material

Supplemental data

Supporting data values

## Version 1 01/08/2026

In-Press Preview

## Version 2 03/09/2026

Electronic publication
