# Supplementary material for: α-Diversity analysis of hepatic transcriptome reveals distinct pathways in alcohol-associated hepatitis
Source: JCI Insight. 2026 Jan 8;11(5):e200727. doi: 10.1172/jci.insight.200727 (PMC13041683; doi:10.1172/jci.insight.200727)
Supplement: Supplemental data [file jciinsight-11-200727-s210.pdf]

SUPPLEMENTAL MATERIAL

Alpha diversity analysis of hepatic transcriptome reveals distinct pathways in alcohol-associated hepatitis.

Sudrishti Chaudhary,[1] Jia-Jun Liu,[2,3] Silvia Liu,[2,3,4] Marissa Di,[5] Juliane I Beier,[1,4] Ramon Bataller,[6] Josepmaria Argemi,[1,7] Panayiotis V Benos,[8] Gavin E Arteel[1,4]

[1]Department of Medicine, Division of Gastroenterology, Hepatology and Nutrition, [2]Pharmacology and Chemical Biology, University of Pittsburgh, PA, USA, [3]Organ Pathobiology and therapeutics institute, University of Pittsburgh, PA, USA, [4]Pittsburgh Liver Research Center, University of Pittsburgh, PA, USA, [5]Department of Computational and Systems Biology, University of Pittsburgh, Pittsburgh, PA, USA, [6]Institut d'Investigacions Biomediques August Pi i Sunyer (IDIBAPS), University of Barcelona, Barcelona, Spain, [7]Department of Internal Medicine, Liver Unit, Clinical University of Navarra, Navarra, Spain, [8]Department of Epidemiology, University of Florida, Gainesville, Florida, USA.

Send all correspondence to:

Gavin E. Arteel, PhD, FAASLD
Thomas E. Starzl Biomedical Science Tower
West 1143
200 Lothrop Street
Pittsburgh, PA 15213
Phone: +1-412-648-4187
Email: gearteel@pitt.edu

Supplemental results

The supplement table S1 represents the liver cellular composition estimated using a reference-based deconvolution approach with 528 validated marker genes across 9 major liver cell types in ALD disease progression. The supplement table S2-S10 lists the significant canonical pathways enriched across all the groups. The tables S2, S3 and S4 represents top canonical pathways enriched in ASH/HC group exclusively by DEG, DSD and commonly by both DEG and DSD approaches respectively. The tables S5, S6 and S7 represents top canonical pathways enriched in AH/HC group exclusively by DEG, DSD and commonly by both DEG and DSD approaches respectively. The tables S8, S9 and S10 represents top canonical pathways enriched in AH/ASH group exclusively by DEG, DSD and commonly by both DEG and DSD approaches respectively.

Supplemental Tables

Table S1: Liver cell type marker genes.

Cell type	Number of genes[1]	Genes employed.[1]
Hepatocytes	57	ALB, APOB, CYP3A4, CYP2E1, CYP1A2, SERPINA1, TF, AFP, HNF4A, CYP2C9, CYP1A1, CYP2A6, CYP2B6, CYP2C8, CYP2C19, CYP2D6, CYP3A5, CYP3A7, G6PC, PCK1, CPS1, FABP1, APOA1, APOA2, APOA4, APOC1, APOC2, APOC3, SLCO1B1, SLCO1B3, ABCB11, ABCC2, SLC22A1, SLC22A7, HNF1A, HNF1B, FOXA2, FOXA3, CEBPA, CEBPB, ONECUT1, ONECUT2, TAT, HPX, FGA, FGB, FGG, AHSG, RBP4, IGF1, ANGPTL3, FTL, AGT, C3, CFB, ORM1, ORM2
Kupffer Cells	58	CD68, CD163, CSF1R, MARCO, MSR1, CD14, LYZ, ITGAM, ADGRE1, FCGR1A, CLEC4F, TIMD4, VSIG4, CD5L, HMOX1, SPIC, ID3, CLEC2D, C1QA, C1QB, C1QC, C3, C4A, C4B, CFB, CFD, CFH, CFI, IL1B, IL6, TNF, IL10, IL12A, IL12B, CXCL10, CCL2, CCL3, MERTK, AXL, TYRO3, FCGR2A, FCGR2B, FCGR3A, SCARA1, SCARA5, IRF4, IRF5, IRF8, STAT1, NFKB1, TYROBP, TREM2, CX3CR1, P2RY12, SIGLEC1, LILRB4, CD206, MRC1
Stellate Cells	59	ACTA2, COL1A1, PDGFRB, TIMP1, MMP2, LRAT, RBP1, TAGLN, CNN1, PDGFRA, COL1A2, COL3A1, COL4A1, COL4A2, COL5A1, COL5A2, COL6A1, COL6A2, COL6A3, MMP1, MMP3, MMP9, MMP13, MMP14, TIMP2, TIMP3, TIMP4, SMTN, MYH11, CALD1, DES, VIM, MSN, TGFB1, TGFBR1, TGFBR2, SMAD2, SMAD3, SMAD4, CTGF, NOX4, PLIN2, PLIN3, ADIPOQ, LEP, PPARG, CEBPA, FABP4, FASN, GFAP, NESTIN, THY1, NT5E, ENG, PECAM1, MCAM, HAND2, TWIST1, SNAI1
Endothelial Cells	59	PECAM1, VWF, CDH5, KDR, FLT1, ICAM1, VCAM1, TEK, ENG, PLVAP, CLDN5, OCLN, TJP1, TJP2, JAM2, JAM3, ESAM, NOS3, EDN1, EDNRA, EDNRB, ANGPT1, ANGPT2, TIE1, ROBO4, LYVE1, STAB1, STAB2, CLEC14A, FCN2, FCN3, CLEC4G, CLEC4M, VEGFA, VEGFB, VEGFC, VEGFD, FLT4, NRP1, NRP2, PDGFA, PDGFB, CD34, CD31, MCAM, THY1, PROCR, THBD, F8, SERPINE1, ADAMTS13, COL4A1, EMCN, ERG, FLI1, SOX17, SOX18, EPAS1, HEY1
T cells	61	CD3D, CD3E, CD3G, CD8A, CD4, IL2RB, GZMB, PRF1, CD2, CD5, TRAC, TRBC1, TRBC2, TRGC1, TRGC2, TRDC, CD247, LCK, ZAP70, IL2, IL4, IL5, IL13, IL17A, IL17F, IL21, IL22, IFNG, TNF, GZMA, GZMH, GZMK, GZMM, GNLY, FASLG, TNFSF10, CCL5, CD25, CD69, CD71, CD95, ICOS, CTLA4, PDCD1, LAG3, TIGIT, HAVCR2, CCR7, SELL, CD127, KLRG1, CX3CR1, CD57, CXCR3, CCR4, CCR6, CXCR5, BCL6, FOXP3, RORC, TBX21
B cells	59	CD19, MS4A1, CD79A, CD79B, IGHM, IGHA1, IGHG1, CD20, PAX5, BLNK, IGHG2, IGHG3, IGHG4, IGHA2, IGHD, IGHE, IGKC, IGLC1, IGLC2, IGLC3, EBF1, TCF3, IKZF1, IKZF3, IRF4, IRF8, PRDM1, XBP1, BCL6, AICDA, CD21, CD23, CD27, CD38, CD138, TNFRSF17, TNFRSF13B, TNFRSF13C, SDC1, JCHAIN, MZB1, DERL3, FKBP11, CR2, FCER2, FCMR, FCRL1, FCRL2, FCRL3, FCRL4, FCRL5, CD22, SIGLEC2, BANK1, SPIB, MEF2C, BACH2, IL4R, CXCR4
NK cells	57	KLRB1, NCR1, FCGR3A, GNLY, PRF1, GZMH, KLRD1, KLRF1, NKG7, NCAM1, KIR2DL1, KIR2DL2, KIR2DL3, KIR2DL4, KIR3DL1, KIR3DL2, KIR3DL3, KIR2DS1, KIR2DS2, KIR2DS3, KIR2DS4, KIR2DS5, KIR3DS1, NCR2, NCR3, KLRK1, CD226, TIGIT, DNAM1, GZMA, GZMB, GZMK, GZMM, FASLG, TNFSF10, CCL3, CCL4, CCL5, CD56, CD16, CD57, CD69, CD25, KLRC1, KLRC2, KLRC3, KLRC4, IL2RB, IL15R, IL12RB2, IFNG, TNF, CSF2, XCL1, XCL2, CXCR1, S1PR5
Neutrophils	58	FCGR3B, CSF3R, CEACAM8, S100A12, ELANE, MPO, CAMP, LTF, DEFA1, DEFA3, DEFA4, AZU1, CTSG, PRTN3, BPI, LCN2, MMP8, MMP9, OLFM4, CXCR1, CXCR2, CXCR4, CCR1, CCR2, CCR3, CCR5, C5AR1, ITGAM, ITGB2, SELL, SELPLG, PADI4, HIST1H2A, HIST1H2B, HIST1H3A, HIST1H4A, G0S2, MNDA, ANXA3, S100A8, S100A9, FFAR2, ALPL, CREB5, GFI1, SPI1, CEBPE, LEF1, NAMPT, VNN1, RETN, TCN1, HP, CEACAM1, CEACAM6, ARG1, IL1R2, TNFAIP6

Monocytes	60	CD14, FCGR1A, S100A8, S100A9, LYZ, VCAN, CCR2, CX3CR1, CSF1R, IRF8, FCGR3A, CCR5, CCR7, SELL, ITGAM, ITGAX, TLR2, TLR4, TLR8, CD163, MAFB, MAF, NR4A1, NR4A2, NR4A3, EGR1, EGR2, FOS, JUN, IL1B, IL6, TNF, CCL2, CCL3, CCL4, CXCL10, MNDA, SIGLEC1, MARCO, MSR1, SCARB1, SPI1, IRF4, KLF4, MAFB, CD86, HLA-DRA, HLA-DRB1, HLA-DQA1, HLA-DQB1, CD68, LILRB2, LILRB4, CLEC7A, CLEC4E, CLEC10A, EMR1, VSIG4, FOLR2, STAB1

[1]Total Markers: 528. Unique Markers: 458. Available in Dataset: 496 (93.9%) Average per Cell Type: 58.7 genes.

Table S2: The top canonical pathways enriched in ASH/HC by DEG

Ingenuity Canonical Pathways	log(p-value)	Ratio	z-score	count
Binding and Uptake of Ligands by Scavenger Receptors	8.67	0.105	-3.464	12
Complement cascade	5.85	0.0735	-3.162	10
Cell surface interactions at the vascular wall	4.87	0.0514	-3.317	11
Immunoregulatory interactions between a Lymphoid and a non-Lymphoid cell	4.85	0.0512	-2.714	11
Receptor-type tyrosine-protein phosphatases	4.35	0.2	1	4
Fcgamma receptor (FCGR) dependent phagocytosis	4.31	0.0549	-3	9
Fc epsilon receptor (FCERI) signaling	3.55	0.0437	-3	9
Signaling by the B Cell Receptor (BCR)	3.43	0.0471	-2.828	8
LXR/RXR Activation	3.42	0.0538	-1.633	7
Mevalonate Pathway I	3.28	0.188	-	3
DHCR24 Signaling Pathway	3.17	0.049	-1.89	7
Intrinsic Prothrombin Activation Pathway	3.02	0.093	-2	4
Superpathway of Geranylgeranyldiphosphate Biosynthesis I (via Mevalonate)	2.98	0.15	-	3
B Cell Development	2.77	0.0265	-	13
Pathogen Induced Cytokine Storm Signaling Pathway	2.7	0.0288	-1.508	11
B Cell Receptor Signaling	2.61	0.0235	-	15
Cholesterol biosynthesis	2.59	0.111	-	3
IL-15 Signaling	2.5	0.0247	-	13
FcγRIIB Signaling in B Lymphocytes	2.46	0.0244	-	13
Systemic Lupus Erythematosus in B Cell Signaling Pathway	2.46	0.022	-	16
Hepatic Fibrosis / Hepatic Stellate Cell Activation	2.44	0.0365	-	7
Superpathway of Cholesterol Biosynthesis	2.42	0.0968	-	3
Potassium Channels	2.39	0.0485	0.447	5
Response of EIF2AK4 (GCN2) to amino acid deficiency	2.39	0.0485	-2.236	5
POU5F1 (OCT4), SOX2, NANOG repress genes related to differentiation	2.36	0.2	-	2
NAD biosynthesis II (from tryptophan)	2.27	0.182	-	2
TRIM21 Intracellular Antibody Signaling Pathway	2.24	0.0229	-3.606	13
Vitamin D (calciferol) metabolism	2.2	0.167	-	2
Specification of primordial germ cells	2.2	0.167	-	2
Hematopoiesis from Multipotent Stem Cells	2.2	0.167	-	2

Systemic Lupus Erythematosus Signaling	2.17	0.0187	-	20
p70S6K Signaling	2.16	0.0224	-	13
Nonsense-Mediated Decay (NMD)	2.15	0.0427	-2.236	5
PI3K Signaling in B Lymphocytes	2.09	0.022	-	13
Eukaryotic Translation Elongation	2.08	0.041	-2.236	5
Eukaryotic Translation Termination	2.08	0.041	-2.236	5
Regulation of Insulin-like Growth Factor (IGF) transport and uptake by IGFBPs	2.05	0.0403	-2.236	5
Activation of gene expression by SREBF (SREBP)	2.05	0.0714	-	3
GP6 Signaling Pathway	1.99	0.0391	-0.447	5
Collagen chain trimerization	1.99	0.0682	-	3
Retinoid metabolism and transport	1.99	0.0682	-	3
Extrinsic Prothrombin Activation Pathway	1.95	0.125	-	2
Signaling by Retinoic Acid	1.94	0.0652	-	3
Interleukin-2 family signaling	1.91	0.0638	-	3
Cachexia Signaling Pathway	1.87	0.0245	-1	9
Wound Healing Signaling Pathway	1.85	0.0281	-1.134	7
SRP-dependent cotranslational protein targeting to membrane	1.81	0.0352	-2.236	5
Communication between Innate and Adaptive Immune Cells	1.78	0.0181	-3.742	17
Selenoamino acid metabolism	1.77	0.0342	-2.236	5
Sensory processing of sound by outer hair cells of the cochlea	1.73	0.0545	-	3
Eukaryotic Translation Initiation	1.72	0.0333	-2.236	5
Branched-chain amino acid catabolism	1.72	0.0952	-	2
Formation of the ureteric bud	1.72	0.0952	-	2
Interleukin-3, Interleukin-5 and GM-CSF signaling	1.71	0.0536	-	3
Regulation of TLR by endogenous ligand	1.68	0.0909	-	2
Extracellular matrix organization	1.62	0.0374	-2	4
Transport of inorganic cations/anions and amino acids/oligopeptides	1.61	0.037	0	4
Coronavirus Replication Pathway	1.55	0.0301	-	5
Integrin signaling	1.51	0.0741	-	2
Collagen biosynthesis and modifying enzymes	1.51	0.0448	-	3
G-Protein Coupled Receptor Signaling	1.5	0.0182	-0.277	13
Amyotrophic Lateral Sclerosis Signaling	1.5	0.0342	0	4
Regulation of lipid metabolism by PPARalpha	1.47	0.0336	1	4
EGR2 and SOX10-mediated initiation of Schwann cell myelination	1.46	0.069	-	2

Osteoarthritis Pathway	1.45	0.0252	-0.447	6
Role of Osteoblasts in Rheumatoid Arthritis Signaling Pathway	1.43	0.025	-2.449	6
cAMP-mediated signaling	1.43	0.0249	0.816	6
ID3 Signaling Pathway	1.4	0.0165	2.5	16
ERK5 Signaling	1.4	0.0405	-	3
NAD Biosynthesis III	1.4	0.25	-	1
Major pathway of rRNA processing in the nucleolus and cytosol	1.37	0.0269	-2.236	5
Plasma lipoprotein assembly, remodeling, and clearance	1.37	0.0395	-	3
Airway Inflammation in Asthma	1.35	0.0606	-	2
TR/RXR Activation	1.34	0.0305	-	4
Maturity Onset Diabetes of Young (MODY) Signaling	1.34	0.0385	-	3
Response to elevated platelet cytosolic Ca2+	1.33	0.0303	-2	4
Acute Phase Response Signaling	1.33	0.0262	-	5
Cellular Effects of Sildenafil (Viagra)	1.31	0.0175	0	12
Coagulation System	1.31	0.0571	-	2
NAD Biosynthesis from 2-amino-3-carboxymuconate Semialdehyde	1.3	0.2	-	1
Protein Citrullination	1.3	0.2	-	1
Serotonin and Melatonin Biosynthesis	1.3	0.2	-	1
NAD Salvage Pathway III	1.3	0.2	-	1
Tyrosine Degradation I	1.3	0.2	-	1

Table S3: The top canonical pathways enriched in ASH/HC by DSD

Ingenuity Canonical Pathways	-log(p-value)	Ratio	z-score	COUNT
Cell Cycle Checkpoints	16	0.14	6.164	38
Kinetochore Metaphase Signaling Pathway	11.4	0.194	2	20
Mitotic Prometaphase	9.42	0.122	5	25
RHO GTPases Activate Formins	8.87	0.143	4.472	20
Mitotic Metaphase and Anaphase	6.87	0.0975	4.796	23
Cohesin Chromatin Regulation Pathway	6.61	0.0913	3.873	24
Estrogen-mediated S-phase Entry	6.55	0.308	2.828	8
Mitotic G1 phase and G1/S transition	6.22	0.121	-1.807	16
Nucleosome assembly	6.2	0.204	3.162	10
TR/RXR Activation	5.56	0.115	3.207	15
Glioblastoma Multiforme Signaling	4.75	0.0936	3	16
Ribonucleotide Reductase Signaling Pathway	4.66	0.092	2.84	16
Molecular Mechanisms of Cancer	4.64	0.0523	4.323	45
TP53 Regulates Transcription of Cell Cycle Genes	4.33	0.163	2.828	8
Replacement of protamines by nucleosomes in the male pronucleus	4.28	0.189	2.646	7
DNA methylation	4.25	0.231	2.449	6
HDR through Homologous Recombination (HRR) or Single Strand Annealing (SSA)	4.2	0.105	3.464	12
DNA Damage/Telomere Stress Induced Senescence	3.9	0.143	2.828	8
Erythropoietin Signaling Pathway	3.89	0.0829	-0.632	15
Breast Cancer Regulation by Stathmin1	3.88	0.0545	4.596	33
Oxidative Stress Induced Senescence	3.87	0.114	3.162	10
RUNX1 regulates megakaryocyte differentiation and platelet function	3.79	0.138	-0.707	8
Role of CHK Proteins in Cell Cycle Checkpoint Control	3.79	0.138	-1.89	8
Chromatin modifications during the maternal to zygotic transition (MZT)	3.72	0.188	2.449	6
Synaptic adhesion-like molecules	3.69	0.238	2.236	5
Kinesins	3.59	0.129	2.828	8
Prostate Cancer Signaling	3.5	0.0948	2.53	11
p53 Signaling	3.49	0.102	-0.333	10
Small Cell Lung Cancer Signaling	3.49	0.102	-	10
Mitotic G2-G2/M phases	3.42	0.075	3.873	15
Granulocyte Adhesion and Diapedesis	3.33	0.0735	-	15

Activation of NMDA receptors and postsynaptic events	3.31	0.106	3	9
Cell Cycle Regulation by BTG Family Proteins	3.29	0.158	1.633	6
Docosahexaenoic Acid (DHA) Signaling	3.28	0.0677	1.5	17
Cyclins and Cell Cycle Regulation	3.27	0.105	2.828	9
Amyloid fiber formation	3.2	0.102	3	9
Neuroinflammation Signaling Pathway	3.13	0.0606	4.123	20
Activated PKN1 stimulates transcription of AR regulated genes KLK2 and KLK3	3.08	0.179	2.236	5
WNT/SHH Axonal Guidance Signaling Pathway	3.06	0.0795	2.887	12
Interleukin-4 and Interleukin-13 signaling	3.06	0.0901	1.265	10
SIRT1 negatively regulates rRNA expression	3	0.172	2.236	5
TNFs bind their physiological receptors	3	0.172	2.236	5
Role of Pattern Recognition Receptors in Recognition of Bacteria and Viruses	2.99	0.0779	2.828	12
Pre-NOTCH Expression and Processing	2.95	0.104	2.828	8
Agranulocyte Adhesion and Diapedesis	2.91	0.067	-	15
Ovarian Cancer Signaling	2.87	0.0755	2	12
Reelin Signaling in Neurons	2.87	0.0797	3.317	11
Hereditary Breast Cancer Signaling	2.87	0.0797	1.265	11
DNA damage-induced 14-3-3σ Signaling	2.84	0.13	0.447	6
Aryl Hydrocarbon Receptor Signaling	2.82	0.071	2.111	13
Degradation of the extracellular matrix	2.8	0.0988	2.828	8
ATM Signaling	2.8	0.09	0.378	9
Chronic Myeloid Leukemia Signaling	2.74	0.0605	3.638	17
Potassium Channels	2.71	0.0874	3	9
Autism Signaling Pathway	2.69	0.0583	3.771	18
Sertoli Cell-Germ Cell Junction Signaling Pathway (Enhanced)	2.69	0.0636	-1.291	15
DNA Replication Pre-Initiation	2.68	0.0865	3	9
Pancreatic Adenocarcinoma Signaling	2.67	0.08	2	10
Mitotic Roles of Polo-Like Kinase	2.66	0.104	1.633	7
PRC2 methylates histones and DNA	2.62	0.143	2.236	5
IL-9 Signaling	2.62	0.143	1	5
Transcriptional regulation of granulopoiesis	2.61	0.118	1.633	6
Cell Cycle: G2/M DNA Damage Checkpoint Regulation	2.61	0.118	-1	6
Glycosaminoglycan metabolism	2.59	0.0781	3.162	10
Cell Cycle: G1/S Checkpoint Regulation	2.58	0.101	-2.646	7

B-WICH complex positively regulates rRNA expression	2.57	0.115	2.449	6
NAP1L1 Transcription Regulation Signaling Pathway	2.54	0.0899	2.121	8
CDX Gastrointestinal Cancer Signaling Pathway	2.54	0.066	-2.887	13
ERCC6 (CSB) and EHMT2 (G9a) positively regulate rRNA expression	2.52	0.135	2.236	5
Fatty acyl-CoA biosynthesis	2.52	0.135	2.236	5
Differential Regulation of Cytokine Production in Intestinal Epithelial Cells by IL-17A and IL-17F	2.5	0.174	2	4
Sertoli Cell-Sertoli Cell Junction Signaling	2.5	0.0607	2.324	15
Glycation Signaling Pathway	2.46	0.0622	2.138	14
Cell junction organization	2.46	0.087	2.828	8
ID1 Signaling Pathway	2.45	0.0644	1.387	13
Meiotic synapsis	2.44	0.109	2.449	6
Senescence Pathway	2.43	0.0565	-1.069	17
Regulation of TP53 Activity through Phosphorylation	2.43	0.086	2.828	8
Senescence-Associated Secretory Phenotype (SASP)	2.41	0.0946	2.646	7
Sheddase Signaling Pathway	2.41	0.0637	3.606	13
Class A/1 (Rhodopsin-like receptors)	2.36	0.0542	4.243	18
Role of PKR in Interferon Induction and Antiviral Response	2.36	0.0725	1.89	10
Preeclampsia Signaling Pathway	2.34	0.0683	-0.905	11
Lung Ionic Balance Signaling Pathway	2.34	0.0479	4.6	25
Triacylglycerol Biosynthesis	2.33	0.103	2.236	6
RET signaling	2.32	0.122	2.236	5
Hematoma Resolution Signaling Pathway	2.31	0.0579	-1.291	15
Coronavirus Pathogenesis Pathway	2.29	0.0616	-0.832	13
DNA Double Strand Break Response	2.29	0.102	2.449	6
Activation of gene expression by SREBF (SREBP)	2.27	0.119	2.236	5
GADD45 Signaling	2.25	0.1	-2.236	6
Regulation of TP53 Activity through Association with Co-factors	2.24	0.214	-	3
Signaling by SCF-KIT	2.23	0.116	2.236	5
Role of BRCA1 in DNA Damage Response	2.23	0.0875	-0.378	7
Mitotic Telophase/Cytokinesis	2.15	0.2	-	3
Stearate Biosynthesis I (Animals)	2.15	0.0952	2.236	6
Tuberculosis Active Signaling Pathway	2.14	0.0571	0.277	14
UFMylation Signaling Pathway	2.12	0.0938	0	6
Activation of anterior HOX genes in hindbrain during early embryogenesis	2.11	0.0833	2.646	7

Apelin Pancreas Signaling Pathway	2.1	0.109	-	5
IL-23 Signaling Pathway	2.1	0.109	2	5
Sleep REM Signaling Pathway	2.09	0.0755	-1.414	8
Cyclophilin Signaling Pathway	2.08	0.0562	2.673	14
S100 Family Signaling Pathway	2.07	0.0422	5.396	33
Pathogen Induced Cytokine Storm Signaling Pathway	2.07	0.0497	4.123	19
Transcriptional regulation by RUNX1	2.07	0.0658	3.162	10
Type II Diabetes Mellitus Signaling	2.07	0.0658	2.236	10
BBSome Signaling Pathway	2.07	0.0467	3.838	23
Sonic Hedgehog Signaling	2.03	0.129	-	4
NoRC negatively regulates rRNA expression	2.02	0.0896	2.449	6
IL-10 Signaling	2.01	0.0645	0	10
Trehalose Degradation II (Trehalase)	1.99	0.333	-	2
Nonhomologous End-Joining (NHEJ)	1.99	0.102	2.236	5
Telomere Maintenance	1.98	0.0787	2.646	7
Meiotic recombination	1.95	0.1	2.236	5
Atherosclerosis Signaling	1.93	0.0662	2.828	9
HMGB1 Signaling	1.92	0.0625	2.449	10
Transcriptional regulation by RUNX2	1.92	0.098	1.342	5
G-Protein Coupled Receptor Signaling	1.92	0.0421	4.017	30
Basal Cell Carcinoma Signaling	1.9	0.0845	1	6
Synaptogenesis Signaling Pathway	1.89	0.0506	3.5	16
CGAS-STING Signaling Pathway	1.89	0.0652	3	9
Hepatitis B Chronic Liver Pathogenesis Signaling Pathway	1.86	0.0585	1.265	11
Mitochondrial Uncoupling	1.85	0.286	-	2
Salvage Pathways of Pyrimidine Deoxyribonucleotides	1.85	0.286	-	2
Role of Osteoblasts in Rheumatoid Arthritis Signaling Pathway	1.85	0.0542	0.277	13
Activation of the pre-replicative complex	1.84	0.114	2	4
HOTAIR Regulatory Pathway	1.83	0.0606	2.828	10
ESR-mediated signaling	1.82	0.0678	2.828	8
RNA Polymerase I Transcription	1.82	0.0811	2.449	6
Non-Small Cell Lung Cancer Signaling	1.81	0.0729	2	7
Colorectal Cancer Metastasis Signaling	1.79	0.0517	3.051	14
Autophagy	1.78	0.0548	1.732	12

Myelination Signaling Pathway	1.75	0.0488	4	16
Nuclear Cytoskeleton Signaling Pathway	1.75	0.0543	3.317	12
CREB Signaling in Neurons	1.75	0.0422	4.6	26
Synthesis of DNA	1.74	0.0656	2.828	8
Neurexins and neuroligins	1.72	0.0877	2.236	5
TCF dependent signaling in response to WNT	1.71	0.0556	3.317	11
Neuropathic Pain Signaling in Dorsal Horn Neurons	1.7	0.0693	2.646	7
Role of IL-17A in Arthritis	1.69	0.0862	-	5
Signaling by MET	1.69	0.0759	2.449	6
TREM1 Signaling	1.69	0.0759	2.236	6
Cellular hexose transport	1.68	0.136	-	3
Pulmonary Healing Signaling Pathway	1.67	0.0547	2.53	11
Role of Macrophages, Fibroblasts and Endothelial Cells in Rheumatoid Arthritis	1.66	0.0475	3.5	16
Mitotic Prophase	1.64	0.0673	2.646	7
GDP-glucose Biosynthesis	1.63	0.222	-	2
Glioma Signaling	1.63	0.0625	-	8
IL-6 Signaling	1.61	0.062	2.236	8
Estrogen-Dependent Breast Cancer Signaling	1.6	0.0723	2.236	6
Tumor Microenvironment Pathway	1.6	0.0556	3	10
Tumoricidal Function of Hepatic Natural Killer Cells	1.58	0.125	-	3
Extracellular matrix organization	1.58	0.0654	2.646	7
Transport of bile salts and organic acids, metal ions and amine compounds	1.58	0.0714	2.449	6
PEDF Signaling	1.58	0.0714	2	6
Irritable Bowel Syndrome Signaling Pathway	1.57	0.0484	2.673	14
Acetyl-CoA Biosynthesis III (from Citrate)	1.57	1	-	1
14-3-3-mediated Signaling	1.56	0.0606	0.447	8
Transport of inorganic cations/anions and amino acids/oligopeptides	1.56	0.0648	2.646	7
Chondroitin Sulfate Biosynthesis (Late Stages)	1.56	0.0794	2	5
Neurotransmitter clearance	1.55	0.2	-	2
mRNA Editing	1.55	0.2	-	2
Glucose and Glucose-1-phosphate Degradation	1.55	0.2	-	2
Regulation of the Epithelial Mesenchymal Transition in Development Pathway	1.53	0.0698	2.236	6
TP53 Regulates Transcription of Cell Death Genes	1.51	0.0909	2	4
Assembly and cell surface presentation of NMDA receptors	1.51	0.0909	2	4

HEY1 Signaling Pathway	1.5	0.0559	3	9
Regulation of mitotic cell cycle	1.49	0.0682	2.449	6
Sirtuin Signaling Pathway	1.49	0.0471	-1.414	14
Interleukin-10 signaling	1.48	0.0889	2	4
Pulmonary Fibrosis Idiopathic Signaling Pathway	1.48	0.046	3.873	15
Activation of IRF by Cytosolic Pattern Recognition Receptors	1.48	0.0758	1	5
Induction of Apoptosis by HIV1	1.48	0.0758	1	5
IL-17 Signaling	1.47	0.0529	3.162	10
CD40 Signaling	1.46	0.0746	1	5
Tuberculosis Latent Signaling Pathway	1.45	0.0667	-1.633	6
Role of IL-17F in Allergic Inflammatory Airway Diseases	1.45	0.087	-	4
Hepatic Fibrosis Signaling Pathway	1.45	0.0433	2.324	18
Pyroptosis	1.45	0.111	-	3
Macrophage Alternative Activation Signaling Pathway	1.43	0.0521	1.667	10
Ephrin A Signaling	1.43	0.0571	2.121	8
IL-17A Signaling in Airway Cells	1.43	0.0735	-	5
Ceramide Signaling	1.43	0.0659	0	6
Wound Healing Signaling Pathway	1.4	0.0482	1.155	12
IL-33 Signaling Pathway	1.4	0.0513	2.121	10
FXR/RXR Activation	1.4	0.0513	-1.667	10
Peptide hormone biosynthesis	1.39	0.167	-	2
GP1b-IX-V activation signalling	1.39	0.167	-	2
HDR through MMEJ (alt-NHEJ)	1.39	0.167	-	2
UDP-N-acetyl-D-galactosamine Biosynthesis II	1.39	0.167	-	2
Role of p14/p19ARF in Tumor Suppression	1.37	0.103	-	3
PFKFB4 Signaling Pathway	1.36	0.0816	-	4
Glioma Invasiveness Signaling	1.36	0.0704	2.236	5
Chondroitin Sulfate Biosynthesis	1.36	0.0704	2	5
Hepatic Cholestasis	1.36	0.0489	2.53	11
Chromatin organization	1.34	0.0471	3.464	12
Melanoma Signaling	1.34	0.08	2	4
MYC Mediated Apoptosis Signaling	1.34	0.08	2	4
WNT/β-catenin Signaling	1.33	0.052	0	9
Advanced glycosylation endproduct receptor signaling	1.33	0.154	-	2

Endocannabinoid Cancer Inhibition Pathway	1.33	0.0544	-2.646	8
IL-15 Production	1.32	0.0574	2.449	7
FAT10 Cancer Signaling Pathway	1.31	0.0784	-	4
RAR Activation	1.31	0.0416	0.943	18
IL-13 Signaling Pathway	1.3	0.0569	1.633	7
Interferon gamma signaling	1.3	0.0612	2.449	6
DNA Methylation and Transcriptional Repression Signaling	1.3	0.0612	2.449	6
Signaling by ALK	1.3	0.0968	-	3

Table S4: The top common pathways enriched in ASH/HC by DEG and DSD

Ingenuity Canonical Pathways	-log(p-value)	Ratio	z-score	Gene count
Binding and Uptake of Ligands by Scavenger Receptors	32.4	0.377	-6.557	43
Complement cascade	27.6	0.309	-6.481	42
Fcgamma receptor (FCGR) dependent phagocytosis	25	0.262	-6.557	43
Cell surface interactions at the vascular wall	22.7	0.215	-6.193	46
Immunoregulatory interactions between a Lymphoid and a non-Lymphoid cell	22.6	0.214	-6.193	46
Fc epsilon receptor (FCERI) signaling	22.5	0.218	-6.557	45
Signaling by the B Cell Receptor (BCR)	20.4	0.229	-6.245	39
B Cell Development	17.7	0.126	-	62
PI3K Signaling in B Lymphocytes	17.2	0.115	-2.646	68
Systemic Lupus Erythematosus in B Cell Signaling Pathway	15.7	0.102	-2.183	74
IL-15 Signaling	15.6	0.116	-	61
B Cell Receptor Signaling	15	0.105	-1.633	67
FcγRIIB Signaling in B Lymphocytes	14.8	0.113	-	60
p70S6K Signaling	14.8	0.108	-2	63
TRIM21 Intracellular Antibody Signaling Pathway	14.7	0.109	-7.62	62
Metallothioneins bind metals	9.49	0.727	-2.828	8
Communication between Innate and Adaptive Immune Cells	8.6	0.0744	-7.814	70
Altered T Cell and B Cell Signaling in Rheumatoid Arthritis	8.19	0.0732	-	69
ID3 Signaling Pathway	8.11	0.0724	6.693	70
Role of NFAT in Regulation of the Immune Response	7.42	0.0688	-1.89	72
Systemic Lupus Erythematosus Signaling	7.08	0.0674	-	72
Mitochondrial RNA degradation	7.04	0.36	3	9
Role of Macrophages, Fibroblasts and Endothelial Cells in Rheumatoid Arthritis	6.94	0.0979	-3.413	33
Primary Immunodeficiency Signaling	6.74	0.21	-	13
Phospholipase C Signaling	6.24	0.0641	-3	72
rRNA processing	6	0.281	3	9
Macrophage Alternative Activation Signaling Pathway	5.96	0.115	-3.578	22
ABRA Signaling Pathway	5.67	0.159	-2.673	14
Activin Inhibin Signaling Pathway	5.25	0.104	-3.411	22
S100 Family Signaling Pathway	5.1	0.0665	-5.181	52
tRNA processing in the mitochondrion	5.02	0.22	3	9

Phagosome Formation	4.37	0.0652	-5.466	46
Neutrophil Extracellular Trap Signaling Pathway	4.29	0.0758	-1.095	31
Cardiac conduction	3.73	0.108	-2.333	14
Glycation Signaling Pathway	3.43	0.0844	-2.524	19
VDR/RXR Activation	3.42	0.128	1.633	10
Hepatic Fibrosis / Hepatic Stellate Cell Activation	3.36	0.0885	-	17
Interleukin-4 and Interleukin-13 signaling	3.29	0.108	-2.887	12
PXR/RXR Activation	3.29	0.134	1.342	9
SPINK1 General Cancer Pathway	3.29	0.134	2.333	9
FXR/RXR Activation	3.29	0.0872	0.577	17
Pulmonary Fibrosis Idiopathic Signaling Pathway	3.28	0.0736	-2.294	24
p53 Signaling	3.2	0.112	1.633	11
Airway Pathology in Chronic Obstructive Pulmonary Disease	3.19	0.105	-	12
Interleukin-10 signaling	3.05	0.156	0.378	7
Agranulocyte Adhesion and Diapedesis	3.03	0.0804	-	18
Role of Chondrocytes in Rheumatoid Arthritis Signaling Pathway	2.78	0.0903	-2.496	13
IL-17A Signaling in Gastric Cells	2.74	0.192	-	5
Transcriptional regulation by RUNX2	2.73	0.137	0.378	7
Superpathway of Citrulline Metabolism	2.72	0.25	-2	4
FLT3 Signaling	2.66	0.154	-2.449	6
Cerebral Malformation Signaling Pathway	2.57	0.094	-0.905	11
Potassium Channels	2.49	0.0971	-3.162	10
Thyronamine and Iodothyronamine Metabolism	2.45	0.667	-	2
Thyroid Hormone Metabolism I (via Deiodination)	2.45	0.667	-	2
Smooth Muscle Contraction	2.44	0.14	-2.449	6
Cellular Effects of Sildenafil (Viagra)	2.44	0.0555	1.151	38
FOXO-mediated transcription	2.42	0.211	-2	4
Aspirin ADME	2.39	0.136	-2.449	6
Sirtuin Signaling Pathway	2.38	0.0673	-3	20
Post-translational protein phosphorylation	2.37	0.0935	-3.162	10
HEY1 Signaling Pathway	2.36	0.0807	-1.155	13
Multiple Sclerosis Signaling Pathway	2.34	0.0731	-1.604	16
Ion channel transport	2.29	0.0765	-3.207	14
RAR Activation	2.26	0.06	-1.961	26

Phenylalanine and tyrosine metabolism	2.25	0.273	-	3
Thyroid Cancer Signaling	2.23	0.103	-1.414	8
Hepatic Cholestasis	2.23	0.0711	-1.941	16
IL-8 Signaling	2.18	0.0721	-2.496	15
IL-17A Signaling in Fibroblasts	2.17	0.1	-1.414	8
Glucocorticoid Receptor Signaling	2.14	0.055	-	33
Role of JAK2 in Hormone-like Cytokine Signaling	2.13	0.108	-1.342	7
Pathogen Induced Cytokine Storm Signaling Pathway	2.07	0.0602	-2.683	23
Leukocyte Extravasation Signaling	2.07	0.0722	-2.496	14
Adipogenesis pathway	2.04	0.0797	0.302	11
Transcriptional regulation of testis differentiation	2.03	0.231	-	3
Hematoma Resolution Signaling Pathway	2	0.0656	1.698	17
CREB Signaling in Neurons	1.98	0.0536	-4.95	33
Tumor Microenvironment Pathway	1.97	0.0722	-1.387	13
Erythropoietin Signaling Pathway	1.95	0.0718	0.277	13
Pulmonary Healing Signaling Pathway	1.95	0.0697	-3.207	14
IL-12 Signaling and Production in Macrophages	1.94	0.0661	2.84	16
Tyrosine Degradation I	1.94	0.4	-	2
Role of IL-17A in Psoriasis	1.94	0.214	-	3
Regulation of Insulin-like Growth Factor (IGF) transport and uptake by IGFBPs	1.93	0.0806	-3.162	10
Bupropion Degradation	1.92	0.154	-	4
Neurotransmitter release cycle	1.91	0.125	-2.236	5
Bone Mineralization Signaling Pathway	1.91	0.0764	0.302	11
Extracellular matrix organization	1.9	0.0841	-3	9
Granulocyte Adhesion and Diapedesis	1.89	0.0686	-	14
G alpha (s) signalling events	1.89	0.0759	-2.714	11
Irritable Bowel Syndrome Signaling Pathway	1.87	0.0623	-1.886	18
Oxidative Phosphorylation	1.87	0.0833	1.414	9
Cell Cycle Control of Chromosomal Replication	1.85	0.105	0	6
Wound Healing Signaling Pathway	1.84	0.0643	-2.5	16
Parkinson's Signaling Pathway	1.8	0.0601	-1.606	19
Post-translational modification: synthesis of GPI-anchored proteins	1.79	0.086	-2.828	8
Arginine Biosynthesis IV	1.78	0.333	-	2
FOXO-mediated transcription of cell death genes	1.77	0.188	-	3

Dilated Cardiomyopathy Signaling Pathway	1.77	0.0728	-0.333	11
GADD45 Signaling	1.74	0.1	-0.816	6
Netrin-1 signaling	1.74	0.114	-2.236	5
Molecular Mechanisms of Cancer	1.72	0.0488	-4.629	42
FOXO-mediated transcription of oxidative stress, metabolic and neuronal genes	1.71	0.133	-2	4
FOXO-mediated transcription of cell cycle genes	1.7	0.176	-	3
IL-10 Signaling	1.69	0.071	0	11
IL-7 Signaling Pathway	1.69	0.0886	-2.236	7
Atherosclerosis Signaling	1.67	0.0735	-1.134	10
CDX Gastrointestinal Cancer Signaling Pathway	1.67	0.066	0.277	13
Role of Osteoblasts in Rheumatoid Arthritis Signaling Pathway	1.66	0.0625	-1.291	15
Transcriptional Regulation by MECP2	1.65	0.0952	-2.449	6
Urea Cycle	1.64	0.286	-	2
Metabolism of amine-derived hormones	1.63	0.167	-	3
Estrogen Biosynthesis	1.63	0.106	-	5
Cardiac Hypertrophy Signaling (Enhanced)	1.62	0.0521	-3.138	28
Bladder Cancer Signaling	1.62	0.0756	-	9
STAT3 Pathway	1.62	0.0719	-1.633	10
Class A/1 (Rhodopsin-like receptors)	1.6	0.0572	-0.229	19
Neuropathic Pain Signaling in Dorsal Horn Neurons	1.6	0.0792	-2.121	8
NAFLD Signaling Pathway	1.58	0.0625	-1.604	14
Gap Junction Signaling	1.57	0.0567	0.243	19
RHO GTPases activate CIT	1.57	0.158	-	3
RHO GTPases Activate ROCKs	1.57	0.158	-	3
IL-15 Production	1.56	0.0738	-2.333	9
Sheddase Signaling Pathway	1.56	0.0637	1.387	13
Aryl Hydrocarbon Receptor Signaling	1.56	0.0656	-	12
NRF2-mediated Oxidative Stress Response	1.55	0.0593	0.447	16
Nicotine Degradation II	1.54	0.0824	-	7
rRNA modification in the mitochondrion	1.53	0.25	-	2
Histidine catabolism	1.53	0.25	-	2
Signaling by NOTCH2	1.52	0.118	-2	4
Role of Osteoclasts in Rheumatoid Arthritis Signaling Pathway	1.52	0.0568	-1.5	18
Role of Osteoblasts, Osteoclasts and Chondrocytes in Rheumatoid Arthritis	1.51	0.0611	-	14

Receptor-type tyrosine-protein phosphatases	1.51	0.15	-	3
PIP3 activates AKT signaling	1.51	0.069	-2.53	10
Sleep REM Signaling Pathway	1.49	0.0755	0	8
IL-17 Signaling	1.47	0.0635	-1.732	12
Respiratory electron transport	1.46	0.0795	1.134	7
Melatonin Degradation III	1.46	1	-	1
Histamine Biosynthesis	1.46	1	-	1
4-hydroxybenzoate Biosynthesis	1.46	1	-	1
4-hydroxyphenylpyruvate Biosynthesis	1.46	1	-	1
ROBO SLIT Signaling Pathway	1.45	0.0703	1	9
GP6 Signaling Pathway	1.45	0.0703	-2.828	9
Striated Muscle Contraction	1.44	0.111	-2	4
Sperm Motility	1.43	0.0584	-1.342	15
Pathogenesis of Multiple Sclerosis	1.43	0.222	-	2
G alpha (i) signalling events	1.42	0.0581	-0.258	15
WNT/SHH Axonal Guidance Signaling Pathway	1.4	0.0662	-1.667	10
Superpathway of Melatonin Degradation	1.4	0.0769	-	7
Regulation of the Epithelial Mesenchymal Transition by Growth Factors Pathway	1.39	0.0619	-2.333	12
TR/RXR Activation	1.39	0.0687	-2.121	9
Mitotic G1 phase and G1/S transition	1.38	0.0682	-1	9
Ribonucleotide Reductase Signaling Pathway	1.37	0.0632	-1	11
G-Protein Coupled Receptor Signaling	1.37	0.0477	-3.087	34
Lymphotoxin β Receptor Signaling	1.37	0.0909	-0.447	5
Role of Cytokines in Mediating Communication between Immune Cells	1.37	0.0909	-	5
Regulation of TP53 Expression and Degradation	1.37	0.105	-1	4
Phase I - Functionalization of compounds	1.35	0.0708	-2.121	8
RAF-independent MAPK1/3 activation	1.35	0.13	-	3
Apelin Cardiac Fibroblast Signaling Pathway	1.35	0.13	-	3
Urea cycle	1.34	0.2	-	2
RUNX1 and FOXP3 control the development of regulatory T lymphocytes (Tregs)	1.34	0.2	-	2
Prednisone ADME	1.34	0.2	-	2
Citrulline Biosynthesis	1.34	0.2	-	2
Lung Ionic Balance Signaling Pathway	1.34	0.0498	-3.922	26
NGF-stimulated transcription	1.33	0.103	-2	4

Nicotine Degradation III	1.33	0.08	-	6
Xenobiotic Metabolism PXR Signaling Pathway	1.33	0.0588	-0.816	13
Class B/2 (Secretin family receptors)	1.31	0.0737	-0.378	7
Human Embryonic Stem Cell Pluripotency	1.31	0.06	-2.887	12

Table S5: The top canonical pathways enriched in AH/HC by DEG analysis

Ingenuity Canonical Pathways	-log(p-value)	Ratio	z-score	Gene count
Heme signaling	2.79	0.0851	1	4
Circadian Clock	2.45	0.069	-2	4
Triacylglycerol Biosynthesis	2.45	0.069	-	4
Interferon gamma signaling	2.38	0.051	-1.342	5
cAMP-mediated signaling	2.32	0.0332	-1.134	8
Stearate Biosynthesis I (Animals)	2.32	0.0635	-	4
Sumoylation Pathway	2.32	0.0495	-	5
Macrophage Alternative Activation Signaling Pathway	2.31	0.0365	-2.236	7
Complement System	2.07	0.0769	-	3
Circadian Rhythm Signaling	2.04	0.0296	-	8
Cardiac β-adrenergic Signaling	1.98	0.0353	-1	6
Vitamin E	1.97	1	-	1
Melatonin Degradation III	1.97	1	-	1
Adenine and Adenosine Salvage VI	1.97	1	-	1
Extrinsic Prothrombin Activation Pathway	1.9	0.125	-	2
PFKFB4 Signaling Pathway	1.8	0.0612	-	3
Complement cascade	1.8	0.0368	-0.447	5
GABA synthesis, release, reuptake and degradation	1.76	0.105	-	2
Apelin Adipocyte Signaling Pathway	1.73	0.0426	-	4
UVB-Induced MAPK Signaling	1.69	0.0556	-	3
Nucleotide salvage	1.6	0.087	-	2
IL-10 Signaling	1.58	0.0323	-	5
GABA receptor activation	1.57	0.05	-	3
Relaxin Signaling	1.57	0.0321	-	5
Serotonin Receptor Signaling	1.5	0.0213	-2.53	10
Xanthine and Xanthosine Salvage	1.5	0.333	-	1
4-aminobutyrate Degradation I	1.5	0.333	-	1
Xenobiotic Metabolism PXR Signaling Pathway	1.48	0.0271	-1.342	6
Phase I - Functionalization of compounds	1.47	0.0354	-1	4
Ribavirin ADME	1.45	0.0448	-	3
PXR/RXR Activation	1.45	0.0448	-	3

CDP-diacylglycerol Biosynthesis I	1.44	0.0714	-	2
Sleep NREM Signaling Pathway	1.43	0.0345	-1	4
Glycolysis I	1.41	0.069	-	2
Sphingosine-1-phosphate Signaling	1.4	0.0336	-	4
FOXO-mediated transcription of oxidative stress, metabolic and neuronal genes	1.39	0.0667	-	2
Phosphatidylglycerol Biosynthesis II (Non-plastidic)	1.39	0.0667	-	2
Gluconeogenesis I	1.39	0.0667	-	2
Threonine catabolism	1.37	0.25	-	1
Guanine and Guanosine Salvage I	1.37	0.25	-	1
Adenine and Adenosine Salvage I	1.37	0.25	-	1
Molybdenum Cofactor Biosynthesis	1.37	0.25	-	1
Ephrin B Signaling	1.36	0.0411	-	3
RAR Activation	1.34	0.0208	-2.333	9
RHO GTPases activate IQGAPs	1.33	0.0625	-	2
MHC class II antigen presentation	1.3	0.0312	-2	4

Table S6: The top canonical pathways enriched in AH/HC by DSD analysis

Ingenuity Canonical Pathways	-log(p-value)	Ratio	z-score	Gene count
RHO GTPases Activate Formins	7.54	0.136	4.243	19
Cohesin Chromatin Regulation Pathway	7.14	0.0989	3.051	26
Pulmonary Fibrosis Idiopathic Signaling Pathway	6.89	0.089	4.707	29
Wound Healing Signaling Pathway	6.44	0.0964	4.082	24
Hepatic Fibrosis / Hepatic Stellate Cell Activation	5.4	0.099	-	19
Interleukin-4 and Interleukin-13 signaling	5.34	0.126	2.138	14
Neutrophil degranulation	4.87	0.0671	5.657	32
Collagen biosynthesis and modifying enzymes	4.62	0.149	3.162	10
Sensory processing of sound by inner hair cells of the cochlea	4.51	0.145	3.162	10
Collagen chain trimerization	4.44	0.182	2.828	8
Mitotic Prometaphase	4.44	0.0878	4.123	18
Agrin Interactions at Neuromuscular Junction	3.8	0.132	2.646	9
Germ Cell-Sertoli Cell Junction Signaling	3.8	0.0877	-	15
PAK Signaling	3.74	0.102	3.162	12
Reelin Signaling in Neurons	3.67	0.0942	2.714	13
ABRA Signaling Pathway	3.6	0.114	2.53	10
Molecular Mechanisms of Cancer	3.57	0.0511	5.947	44
L1CAM interactions	3.54	0.0968	3.317	12
Semaphorin Signaling in Neurons	3.51	0.136	-	8
Arachidonic acid metabolism	3.51	0.156	2.646	7
Axonal Guidance Signaling	3.48	0.058	-	30
Ion channel transport	3.48	0.082	3.873	15
Synaptogenesis Signaling Pathway	3.35	0.0665	4.025	21
Synthesis of Lipoxins (LX)	3.33	0.5	-	3
Collagen degradation	3.27	0.125	2.828	8
Semaphorin interactions	3.27	0.125	2.828	8
G alpha (12/13) signalling events	3.27	0.113	3	9
Interferon gamma signaling	3.22	0.102	3.162	10
Crosstalk between Dendritic Cells and Natural Killer Cells	3.22	0.102	2	10
Interferon Signaling	3.18	0.162	2	6

Gap junction trafficking and regulation	3.17	0.137	2.449	7
Multiple Sclerosis Signaling Pathway	3.12	0.0731	3.873	16
p75 NTR receptor-mediated signalling	3.1	0.0823	3.606	13
Epithelial Adherens Junction Signaling	3.1	0.0823	0.577	13
GPER1 signaling	3.05	0.0971	3.162	10
ID1 Signaling Pathway	3.03	0.0743	2.138	15
Ephrin Receptor Signaling	3.03	0.0743	3.162	15
Signaling by Rho Family GTPases	3.02	0.0674	3.742	18
Natural Killer Cell Signaling	3.01	0.0739	1.732	15
Leukotriene Biosynthesis	3	0.25	-	4
Sensory processing of sound by outer hair cells of the cochlea	2.97	0.127	2.646	7
Parkinson's Signaling Pathway	2.96	0.0633	0.943	20
Cell Cycle Checkpoints	2.93	0.0662	4.243	18
Cell junction organization	2.83	0.0978	3	9
Cardiac conduction	2.81	0.0846	2.828	11
Serotonin Receptor Signaling	2.8	0.0553	4.082	26
Phagosome Maturation	2.79	0.076	-	13
Mitotic Metaphase and Anaphase	2.78	0.0678	3.873	16
Assembly and cell surface presentation of NMDA receptors	2.77	0.136	2.236	6
Aggrephagy	2.77	0.136	2.236	6
Interferon alpha/beta signaling	2.77	0.105	2.828	8
Regulation of the Epithelial Mesenchymal Transition by Growth Factors Pathway	2.75	0.0722	3.606	14
Class B/2 (Secretin family receptors)	2.73	0.0947	3	9
Role of MAPK Signaling in Promoting the Pathogenesis of Influenza	2.72	0.0877	3.162	10
RAF/MAP kinase cascade	2.71	0.0649	4	17
Assembly of collagen fibrils and other multimeric structures	2.71	0.115	2.646	7
Autism Signaling Pathway	2.69	0.0615	4.123	19
ILK Signaling	2.69	0.0711	2.309	14
Caveolar-mediated Endocytosis Signaling	2.66	0.101	2.236	8
Kinesins	2.66	0.113	2.449	7
RHOGDI Signaling	2.66	0.0682	-2.887	15
RHO GTPases activate IQGAPs	2.66	0.156	2	5
Nuclear Cytoskeleton Signaling Pathway	2.64	0.0679	3.742	15
Human Embryonic Stem Cell Pluripotency	2.62	0.07	3.742	14

CGAS-STING Signaling Pathway	2.6	0.0797	3.317	11
Tumor Microenvironment Pathway	2.59	0.0722	2.309	13
mRNA Editing	2.59	0.3	-	3
CREB Signaling in Neurons	2.58	0.0503	4.707	31
Role of Osteoclasts in Rheumatoid Arthritis Signaling Pathway	2.57	0.0599	2.982	19
Virus Entry via Endocytic Pathways	2.55	0.0833	2.828	10
Role of MAPK Signaling in the Pathogenesis of Influenza	2.5	0.0952	-	8
Kinetochore Metaphase Signaling Pathway	2.49	0.0874	1.414	9
Abacavir ADME	2.47	0.104	2.646	7
Mitotic Roles of Polo-Like Kinase	2.47	0.104	1	7
Prostanoid Biosynthesis	2.46	0.273	-	3
Neurovascular Coupling Signaling Pathway	2.44	0.0647	3.357	15
RHO GTPase cycle	2.43	0.0533	4.899	24
Myelination Signaling Pathway	2.41	0.0579	3.441	19
Hematoma Resolution Signaling Pathway	2.38	0.0618	-1.5	16
PPAR Signaling	2.35	0.0833	-3	9
Pathogen Induced Cytokine Storm Signaling Pathway	2.34	0.055	4.472	21
Glioblastoma Multiforme Signaling	2.34	0.0702	3.162	12
Glioma Invasiveness Signaling	2.33	0.0986	1.89	7
Remodeling of Epithelial Adherens Junctions	2.33	0.0986	-	7
PTEN Signaling	2.3	0.0728	-2.53	11
Translocation of SLC2A4 (GLUT4) to the plasma membrane	2.3	0.0972	2.449	7
TR/RXR Activation	2.28	0.0763	1.414	10
Regulation of Actin-based Motility by Rho	2.27	0.0811	2.646	9
Transcriptional regulation of testis differentiation	2.24	0.231	-	3
DNA Damage/Telomere Stress Induced Senescence	2.24	0.107	2.449	6
O-linked glycosylation	2.22	0.0796	3	9
HSP90 chaperone cycle for steroid hormone receptors in the presence of ligand	2.2	0.105	2.236	6
Sertoli Cell-Sertoli Cell Junction Signaling	2.19	0.0607	3.207	15
Colorectal Cancer Metastasis Signaling	2.19	0.059	3.742	16
Oxytocin in Brain Signaling Pathway	2.19	0.0647	2.496	13
Macropinocytosis Signaling	2.17	0.0921	2	7
Signaling by PDGF	2.17	0.103	2.449	6
Cancer Drug Resistance by Drug Efflux	2.17	0.103	2	6

Breast Cancer Regulation by Stathmin1	2.16	0.0479	4.6	29
RAC Signaling	2.15	0.073	3	10
Intra-Golgi and retrograde Golgi-to-ER traffic	2.14	0.0637	3.464	13
HMGB1 Signaling	2.12	0.0688	3.162	11
STAT3 Pathway	2.11	0.0719	3	10
GABAergic Receptor Signaling Pathway (Enhanced)	2.08	0.0714	0.333	10
Sphingosine-1-phosphate Signaling	2.08	0.0756	1.667	9
Signaling by MET	2.08	0.0886	2.646	7
Eicosanoid Signaling	2.03	0.0567	4	16
Interconversion of nucleotide di- and triphosphates	2.02	0.138	2	4
EGR2 and SOX10-mediated initiation of Schwann cell myelination	2.02	0.138	2	4
Renin-Angiotensin Signaling	2.01	0.0738	2.449	9
G alpha (s) signalling events	1.98	0.069	3.162	10
Osteoarthritis Pathway	1.97	0.0588	1.897	14
Potassium Channels	1.97	0.0777	2.828	8
Semaphorin Neuronal Repulsive Signaling Pathway	1.94	0.068	-1.265	10
Role of Osteoblasts in Rheumatoid Arthritis Signaling Pathway	1.94	0.0583	1.604	14
Cellular Effects of Sildenafil (Viagra)	1.94	0.0453	-1.461	31
Activation of anterior HOX genes in hindbrain during early embryogenesis	1.94	0.0833	2.449	7
Integrin cell surface interactions	1.91	0.0824	2.646	7
Calcium Signaling	1.91	0.0596	3.162	13
Paxillin Signaling	1.9	0.0755	2.646	8
MHC class II antigen presentation	1.88	0.0703	2.828	9
GP6 Signaling Pathway	1.88	0.0703	3	9
ERK/MAPK Signaling	1.88	0.0591	1.897	13
Protein Kinase A Signaling	1.88	0.0505	0.775	20
Extracellular matrix organization	1.88	0.0748	2.828	8
Ribavirin ADME	1.87	0.0896	2.449	6
PDGF Signaling	1.86	0.0805	2.646	7
Nucleosome assembly	1.86	0.102	2.236	5
Amyloid fiber formation	1.83	0.0795	2.646	7
S100 Family Signaling Pathway	1.83	0.0435	4.382	34
PI3K/AKT Signaling	1.82	0.06	2.121	12
Role of Pattern Recognition Receptors in Recognition of Bacteria and Viruses	1.81	0.0649	2	10

Role of JAK1 and JAK3 in γc Cytokine Signaling	1.81	0.087	2.449	6
NAP1L1 Transcription Regulation Signaling Pathway	1.81	0.0787	2.449	7
Regulation of Cellular Mechanics by Calpain Protease	1.81	0.0787	1.342	7
Pulmonary Healing Signaling Pathway	1.81	0.0597	3.464	12
14-3-3-mediated Signaling	1.8	0.0682	2.646	9
Transcriptional regulation of granulopoiesis	1.79	0.098	2.236	5
Cell Cycle: G2/M DNA Damage Checkpoint Regulation	1.79	0.098	0	5
FOXO-mediated transcription	1.77	0.158	-	3
IL-27 Signaling Pathway	1.77	0.0672	1.414	9
Sheddase Signaling Pathway	1.76	0.0588	1.155	12
Ceramide Signaling	1.76	0.0769	1.633	7
Actin Nucleation by ARP-WASP Complex	1.76	0.0769	2.449	7
B-WICH complex positively regulates rRNA expression	1.75	0.0962	2.236	5
Neuroinflammation Signaling Pathway	1.75	0.0515	1.604	17
Xenobiotic Metabolism Signaling	1.73	0.0514	-	17
IL-9 Signaling	1.73	0.114	2	4
CCR3 Signaling in Eosinophils	1.73	0.0662	2.449	9
PD-1, PD-L1 cancer immunotherapy pathway	1.72	0.0702	0.378	8
G-Protein Coupled Receptor Signaling	1.72	0.0435	4.382	31
EPH-Ephrin signaling	1.71	0.0753	2.646	7
Role of Tissue Factor in Cancer	1.7	0.0577	2.714	12
Role of PKR in Interferon Induction and Antiviral Response	1.69	0.0652	1.89	9
Striated Muscle Contraction	1.69	0.111	2	4
Sleep NREM Signaling Pathway	1.68	0.069	-0.378	8
Integrin Signaling	1.67	0.0571	3.464	12
CLEAR Signaling Pathway	1.67	0.0526	-2.84	15
Immunogenic Cell Death Signaling Pathway	1.67	0.0737	2.449	7
Sertoli Cell-Germ Cell Junction Signaling Pathway (Enhanced)	1.65	0.0551	-0.277	13
Formation of the ureteric bud	1.65	0.143	-	3
HOTAIR Regulatory Pathway	1.63	0.0606	3	10
Irritable Bowel Syndrome Signaling Pathway	1.62	0.0519	2.84	15
TGF-β Signaling	1.62	0.0722	1.633	7
Cachexia Signaling Pathway	1.62	0.0489	3.153	18
Inhibition of Matrix Metalloproteases	1.61	0.105	-	4

Immunoregulatory interactions between a Lymphoid and a non-Lymphoid cell	1.6	0.0558	3.464	12
Neurexins and neuroligins	1.6	0.0877	2	5
Hedgehog 'on' state	1.59	0.0625	3	9
Protein folding	1.58	0.0707	2.449	7
Actin Cytoskeleton Signaling	1.58	0.0537	2.53	13
Orexin Signaling Pathway	1.58	0.0537	3.051	13
NGF-stimulated transcription	1.58	0.103	2	4
VDR/RXR Activation	1.57	0.0769	-	6
PKR-mediated signaling	1.57	0.0769	2.236	6
RHO GTPases Activate Rhotekin and Rhophilins	1.57	0.222	-	2
RUNX1 regulates megakaryocyte differentiation and platelet function	1.57	0.0862	-0.447	5
NOD1/2 Signaling Pathway	1.57	0.057	3.162	11
Cardiac Hypertrophy Signaling (Enhanced)	1.56	0.0447	4.359	24
Phagosome Formation	1.56	0.0425	5.014	30
Regulation of the Epithelial-Mesenchymal Transition Pathway	1.55	0.0567	-	11
AMPK Signaling	1.55	0.0533	0.816	13
NRF2-mediated Oxidative Stress Response	1.55	0.0519	2.828	14
Tuberculosis Active Signaling Pathway	1.54	0.0531	-1.732	13
FXR/RXR Activation	1.54	0.0564	-0.302	11
Gαi Signaling	1.54	0.0612	1.134	9
Asparagine Biosynthesis I	1.53	1	-	1
COPI-mediated anterograde transport	1.52	0.0686	2.449	7
Role of JAK family kinases in IL-6-type Cytokine Signaling	1.5	0.0741	2.449	6
Thrombin Signaling	1.5	0.0538	2.121	12
Adrenergic Receptor Signaling Pathway (Enhanced)	1.5	0.0556	0	11
Other interleukin signaling	1.5	0.125	-	3
Interleukin-6 family signaling	1.5	0.125	-	3
Agranulocyte Adhesion and Diapedesis	1.48	0.0536	-	12
Interleukin-9 signaling	1.48	0.2	-	2
RHOBTB3 ATPase cycle	1.48	0.2	-	2
Gαs Signaling	1.48	0.063	2.646	8
Netrin Signaling	1.47	0.0571	0.632	10
Mitotic G2-G2/M phases	1.47	0.055	3.162	11
Oncostatin M Signaling	1.44	0.093	2	4

Antigen Presentation Pathway	1.44	0.093	-	4
Granulocyte Adhesion and Diapedesis	1.42	0.0539	-	11
Activation of NMDA receptors and postsynaptic events	1.42	0.0706	2	6
Retinoid metabolism and transport	1.41	0.0909	2	4
DNA methylation	1.41	0.115	-	3
Integration of energy metabolism	1.4	0.0648	2.646	7
Phenylalanine and tyrosine metabolism	1.4	0.182	-	2
GABA Receptor Signaling	1.38	0.0602	-	8
Carboxyterminal post-translational modifications of tubulin	1.38	0.0889	-	4
Syndecan interactions	1.36	0.111	-	3
Microautophagy Signaling Pathway	1.35	0.0566	-	9
DNA damage-induced 14-3-3σ Signaling	1.35	0.087	-1	4
ERBB4 Signaling	1.34	0.0746	2	5
Telomere Maintenance	1.33	0.0674	2.449	6
Atherosclerosis Signaling	1.33	0.0588	2.828	8
RHO GTPases activate PKNs	1.32	0.107	-	3
Activated PKN1 stimulates transcription of AR regulated genes KLK2 and KLK3	1.32	0.107	-	3
Preeclampsia Signaling Pathway	1.32	0.0559	-0.333	9
Interleukin-2 family signaling	1.32	0.0851	2	4
Glutamate Receptor Signaling	1.31	0.0735	-	5
Hepatitis B Chronic Liver Pathogenesis Signaling Pathway	1.3	0.0532	1.414	10

Table S7: The top common pathways enriched in AH/HC by DEG and DSD analysis

Ingenuity Canonical Pathways	-log(p-value)	Ratio	z-score	Gene count
Extracellular matrix organization	17.2	0.467	4.243	50
Molecular Mechanisms of Cancer	15.8	0.226	2.691	195
Sheddase Signaling Pathway	14.9	0.343	3.586	70
LXR/RXR Activation	13.7	0.392	-4.422	51
DHCR24 Signaling Pathway	13.6	0.378	-3.781	54
S100 Family Signaling Pathway	13.1	0.221	3.086	173
Regulation of Insulin-like Growth Factor (IGF) transport and uptake by IGFBPs	12.7	0.387	0.289	48
Tumor Microenvironment Pathway	12.3	0.333	4.106	60
Atherosclerosis Signaling	12.2	0.368	0	50
Acute Phase Response Signaling	12.1	0.325	0.949	62
Phase I - Functionalization of compounds	11.8	0.389	-4.529	44
Integrin cell surface interactions	11.7	0.435	4.11	37
Wound Healing Signaling Pathway	11.1	0.289	2.151	72
Post-translational protein phosphorylation	10.8	0.383	0.469	41
Coagulation System	10.3	0.6	-1.606	21
Interleukin-4 and Interleukin-13 signaling	10.2	0.369	1.093	41
Role of Osteoclasts in Rheumatoid Arthritis Signaling Pathway	9.85	0.259	4.129	82
Hepatic Cholestasis	9.65	0.284	2.84	64
Hematoma Resolution Signaling Pathway	9.4	0.27	-2.183	70
Axonal Guidance Signaling	9.36	0.224	-	116
Breast Cancer Regulation by Stathmin1	9.36	0.216	1.782	131
LPS/IL-1 Mediated Inhibition of RXR Function	9.27	0.258	1.091	77
Pulmonary Fibrosis Idiopathic Signaling Pathway	9.21	0.252	5.143	82
Response to elevated platelet cytosolic Ca2+	9.18	0.333	-0.302	44
Phagosome Formation	8.94	0.207	1.623	146
Granulocyte Adhesion and Diapedesis	8.8	0.284	-	58
Aspirin ADME	8.65	0.5	-3.411	22
Hepatic Fibrosis / Hepatic Stellate Cell Activation	8.51	0.286	-	55
IL-6 Signaling	8.45	0.326	1.947	42
RAR Activation	8.22	0.226	-0.308	98

Role of IL-17A in Arthritis	8.05	0.431	-	25
Sertoli Cell-Sertoli Cell Junction Signaling	7.88	0.259	2.898	64
G-Protein Coupled Receptor Signaling	7.87	0.201	0.256	143
FXR/RXR Activation	7.81	0.277	-0.146	54
Collagen degradation	7.69	0.406	3.53	26
HEY1 Signaling Pathway	7.66	0.292	2.771	47
Irritable Bowel Syndrome Signaling Pathway	7.62	0.246	2.492	71
Docosahexaenoic Acid (DHA) Signaling	7.59	0.255	0.128	64
Cellular Effects of Sildenafil (Viagra)	7.49	0.2	-0.26	137
Xenobiotic Metabolism Signaling	7.46	0.236	-	78
Lung Ionic Balance Signaling Pathway	7.34	0.211	0.191	110
Formation of Fibrin Clot (Clotting Cascade)	7.32	0.487	-2.065	19
CREB Signaling in Neurons	7.26	0.203	0.093	125
Hepatic Fibrosis Signaling Pathway	7.25	0.221	2.491	92
IL-8 Signaling	7.19	0.264	3.429	55
Bupropion Degradation	7.17	0.577	-	15
Sertoli Cell-Germ Cell Junction Signaling Pathway (Enhanced)	7.11	0.254	0	60
HIF1α Signaling	7.04	0.262	1.236	55
Glycosaminoglycan metabolism	7.02	0.305	3.244	39
Cachexia Signaling Pathway	7	0.226	2.111	83
Serotonin Receptor Signaling	6.95	0.213	1.91	100
Activin Inhibin Signaling Pathway	6.89	0.259	2.292	55
Senescence Pathway	6.87	0.236	2.047	71
ID1 Signaling Pathway	6.84	0.262	0.832	53
Assembly of collagen fibrils and other multimeric structures	6.84	0.393	4.082	24
Agranulocyte Adhesion and Diapedesis	6.81	0.254	-	57
Dissolution of Fibrin Clot	6.67	0.769	1.265	10
Nicotine Degradation III	6.67	0.36	-0.333	27
p53 Signaling	6.64	0.327	0.655	32
Leukocyte Extravasation Signaling	6.63	0.263	2.592	51
IL-17A Signaling in Fibroblasts	6.59	0.35	2.887	28
Nicotine Degradation II	6.53	0.341	-0.632	29
Airway Pathology in Chronic Obstructive Pulmonary Disease	6.46	0.307	-0.378	35
Estrogen Biosynthesis	6.46	0.426	0	20

IL-17A Signaling in Airway Cells	6.42	0.368	1.147	25
Class A/1 (Rhodopsin-like receptors)	6.42	0.226	0.577	75
HMGB1 Signaling	6.4	0.275	2.611	44
Production of Nitric Oxide and Reactive Oxygen Species in Macrophages	6.38	0.26	-0.603	50
Cardiac conduction	6.34	0.292	-1.3	38
Clathrin-mediated Endocytosis Signaling	6.33	0.254	-	53
WNT/SHH Axonal Guidance Signaling Pathway	6.29	0.278	1.718	42
Colorectal Cancer Metastasis Signaling	6.28	0.236	2.994	64
Retinoid metabolism and transport	6.28	0.432	0.229	19
Aryl Hydrocarbon Receptor Signaling	6.25	0.262	0.209	48
IL-33 Signaling Pathway	6.16	0.256	3.202	50
Bile acid and bile salt metabolism	6.1	0.422	-2.524	19
Transport of bile salts and organic acids, metal ions and amine compounds	6.1	0.333	-0.378	28
Glycation Signaling Pathway	5.99	0.244	3.641	55
Bladder Cancer Signaling	5.97	0.294	1.897	35
PXR/RXR Activation	5.96	0.358	0.832	24
CD40 Signaling	5.96	0.358	1.706	24
Preeclampsia Signaling Pathway	5.9	0.267	1.677	43
Regulation of the Epithelial Mesenchymal Transition by Growth Factors Pathway	5.85	0.253	2.846	49
Semaphorin Neuronal Repulsive Signaling Pathway	5.76	0.272	0.174	40
Pathogen Induced Cytokine Storm Signaling Pathway	5.7	0.212	2.692	81
CDX Gastrointestinal Cancer Signaling Pathway	5.64	0.249	-1.896	49
Elastic fibre formation	5.57	0.409	3.3	18
Germ Cell-Sertoli Cell Junction Signaling	5.55	0.257	-	44
Glutaminergic Receptor Signaling Pathway (Enhanced)	5.51	0.218	0.361	71
Melatonin Degradation I	5.45	0.318	-0.333	27
Activation of Matrix Metalloproteinases	5.42	0.455	0.775	15
Arachidonic acid metabolism	5.4	0.4	-1.213	18
Acetone Degradation I (to Methylglyoxal)	5.39	0.415	1	17
Collagen biosynthesis and modifying enzymes	5.37	0.343	2.294	23
Degradation of the extracellular matrix	5.36	0.321	3.922	26
NAFLD Signaling Pathway	5.35	0.237	1.786	53
Superpathway of Melatonin Degradation	5.32	0.308	-0.632	28
Paxillin Signaling	5.31	0.292	1.964	31

IL-12 Signaling and Production in Macrophages	5.3	0.231	2.496	56
Role of Tissue Factor in Cancer	5.29	0.24	2.263	50
Inhibition of Matrix Metalloproteases	5.21	0.421	-2.138	16
HOTAIR Regulatory Pathway	5.2	0.255	3.363	42
GP6 Signaling Pathway	5.17	0.273	2.535	35
Pancreatic Adenocarcinoma Signaling	4.99	0.272	1.789	34
Actin Cytoskeleton Signaling	4.97	0.227	2.335	55
Type II Diabetes Mellitus Signaling	4.97	0.257	1.46	39
Collagen chain trimerization	4.9	0.386	1.698	17
Autism Signaling Pathway	4.88	0.214	1.664	66
Osteoarthritis Pathway	4.87	0.227	1.859	54
Plasma lipoprotein assembly, remodeling, and clearance	4.87	0.316	-1.225	24
Role of JAK family kinases in IL-6-type Cytokine Signaling	4.85	0.309	1.043	25
Xenobiotic Metabolism CAR Signaling Pathway	4.83	0.227	-0.186	53
IL-10 Signaling	4.75	0.252	0.973	39
G alpha (i) signalling events	4.75	0.221	-0.132	57
NRF2-mediated Oxidative Stress Response	4.74	0.219	1.091	59
Eicosanoid Signaling	4.73	0.216	2.474	61
BBSome Signaling Pathway	4.72	0.193	-0.314	95
GP1b-IX-V activation signalling	4.69	0.667	1.414	8
L1CAM interactions	4.65	0.266	1.219	33
Neutrophil degranulation	4.61	0.193	3.753	92
ERK/MAPK Signaling	4.58	0.227	2.197	50
Myelination Signaling Pathway	4.56	0.207	2.81	68
Signaling by MET	4.55	0.304	2.449	24
Role of MAPK Signaling in Inhibiting the Pathogenesis of Influenza	4.55	0.304	0.894	24
Regulation of Cellular Mechanics by Calpain Protease	4.55	0.292	1.508	26
Cardiac Hypertrophy Signaling (Enhanced)	4.53	0.188	2.429	101
Human Embryonic Stem Cell Pluripotency	4.41	0.23	0.295	46
PI3K/AKT Signaling	4.41	0.23	1.706	46
IL-17 Signaling	4.38	0.233	3.395	44
Role of Chondrocytes in Rheumatoid Arthritis Signaling Pathway	4.38	0.25	2.535	36
Glioma Invasiveness Signaling	4.37	0.31	1.528	22
Cerebral Malformation Signaling Pathway	4.37	0.265	0.539	31

Noradrenaline and Adrenaline Degradation	4.36	0.385	-2.53	15
Syndecan interactions	4.33	0.444	2.887	12
IL-13 Signaling Pathway	4.32	0.26	1.095	32
Pancreatic Secretion Signaling Pathway	4.3	0.218	1.664	53
Signaling by Rho Family GTPases	4.3	0.213	2.53	57
Cell surface interactions at the vascular wall	4.27	0.224	3.464	48
Cell junction organization	4.27	0.283	2.746	26
Ephrin A Signaling	4.27	0.25	1.372	35
ILK Signaling	4.25	0.228	0.649	45
Triacylglycerol Biosynthesis	4.22	0.328	-0.258	19
Xenobiotic Metabolism PXR Signaling Pathway	4.22	0.222	-1.512	49
Neuroinflammation Signaling Pathway	4.21	0.203	3	67
Integrin Signaling	4.18	0.224	2.03	47
Erythropoietin Signaling Pathway	4.17	0.232	-0.16	42
Ethanol Degradation II	4.17	0.389	-2.333	14
Estrogen-Dependent Breast Cancer Signaling	4.17	0.289	2	24
Phase II - Conjugation of compounds	4.16	0.266	-3.157	29
Regulation of the Epithelial-Mesenchymal Transition Pathway	4.09	0.227	-	44
MSP-RON Signaling in Cancer Cells Pathway	4.07	0.245	2.611	35
Ion channel transport	4.06	0.23	0.617	42
CGAS-STING Signaling Pathway	4.03	0.246	4.352	34
Superoxide Radicals Degradation	4.03	0.75	0.816	6
Parkinson's Signaling Pathway	4.02	0.203	-0.128	64
Serotonin Degradation	4.02	0.274	-2.324	26
Gap Junction Signaling	4.01	0.2	1	67
GADD45 Signaling	4	0.317	-0.229	19
Role of IL-17F in Allergic Inflammatory Airway Diseases	4	0.348	2.333	16
RAF/MAP kinase cascade	3.97	0.21	2.1	55
Mitochondrial RNA degradation	3.96	0.44	3.317	11
Metallothioneins bind metals	3.96	0.636	-2.646	7
Paracetamol ADME	3.96	0.414	0	12
FGF Signaling	3.9	0.279	1.877	24
PAK Signaling	3.89	0.254	1.414	30
Adrenergic Receptor Signaling Pathway (Enhanced)	3.88	0.222	-2.412	44

Role of Osteoblasts in Rheumatoid Arthritis Signaling Pathway	3.86	0.212	1.82	51
O-linked glycosylation	3.86	0.257	2.414	29
4-1BB Signaling in T Lymphocytes	3.83	0.382	1.897	13
Intrinsic Prothrombin Activation Pathway	3.8	0.349	-0.832	15
SPINK1 General Cancer Pathway	3.78	0.299	2.524	20
IL-1 Signaling	3.78	0.265	1.5	26
TR/RXR Activation	3.76	0.244	-0.186	32
HGF Signaling	3.76	0.244	1.414	32
Glioblastoma Multiforme Signaling	3.75	0.228	1.616	39
ERBB Signaling	3.75	0.269	2.449	25
Pulmonary Healing Signaling Pathway	3.72	0.219	2.714	44
Maturity Onset Diabetes of Young (MODY) Signaling	3.7	0.282	-	22
Neurotrophin/TRK Signaling	3.7	0.282	0.243	22
Reelin Signaling in Neurons	3.67	0.239	1.257	33
RHOGDI Signaling	3.67	0.214	-2.043	47
eNOS Signaling	3.66	0.23	0.2	37
IGF-1 Signaling	3.65	0.257	0	27
Prostate Cancer Signaling	3.65	0.25	1.347	29
Atorvastatin ADME	3.61	0.667	-2.449	6
Ribonucleotide Reductase Signaling Pathway	3.59	0.224	3	39
CDK5 Signaling	3.58	0.255	0	27
PPARα/RXRα Activation	3.57	0.217	-0.507	43
Gα12/13 Signaling	3.57	0.239	2.117	32
Activation of NMDA receptors and postsynaptic events	3.55	0.271	0.853	23
LPS-stimulated MAPK Signaling	3.55	0.271	1.789	23
Gαq Signaling	3.54	0.225	1.3	38
Lymphotoxin β Receptor Signaling	3.5	0.309	2.138	17
Apelin Pancreas Signaling Pathway	3.43	0.326	0.535	15
IL-23 Signaling Pathway	3.43	0.326	0.535	15
GDNF Family Ligand-Receptor Interactions	3.42	0.276	0.243	21
RAC Signaling	3.39	0.234	2.4	32
Platelet Aggregation (Plug Formation)	3.35	0.538	0.378	7
Toll-like Receptor Signaling	3.34	0.273	2	21
Synaptogenesis Signaling Pathway	3.28	0.193	2.364	61

STAT3 Pathway	3.27	0.23	0.218	32
NF-κB Activation by Viruses	3.26	0.269	2.524	21
Fructose metabolism	3.26	0.714	-2.236	5
Neurotransmitter clearance	3.26	0.6	-1.633	6
Creatine metabolism	3.26	0.6	0	6
Prednisone ADME	3.26	0.6	-2.449	6
Role of Osteoblasts, Osteoclasts and Chondrocytes in Rheumatoid Arthritis	3.26	0.205	-	47
Thrombin Signaling	3.25	0.206	1.667	46
PTEN Signaling	3.25	0.225	-0.447	34
NAP1L1 Transcription Regulation Signaling Pathway	3.24	0.258	2.711	23
Role of Macrophages, Fibroblasts and Endothelial Cells in Rheumatoid Arthritis	3.22	0.19	2.433	64
NCAM signaling for neurite out-growth	3.21	0.286	1.886	18
Sleep REM Signaling Pathway	3.2	0.245	-1.961	26
Signaling by PDGF	3.2	0.293	2.668	17
Hepatitis B Chronic Liver Pathogenesis Signaling Pathway	3.18	0.213	2.53	40
Neurovascular Coupling Signaling Pathway	3.13	0.203	0.457	47
Non-Small Cell Lung Cancer Signaling	3.13	0.25	1.807	24
Tryptophan catabolism	3.1	0.5	-1.89	7
Role of IL-17A in Psoriasis	3.1	0.5	1.342	7
Ephrin Receptor Signaling	3.09	0.208	1.706	42
IL-17A Signaling in Gastric Cells	3.09	0.385	1.134	10
Nucleotide catabolism	3.07	0.343	-1.732	12
IL-9 Signaling	3.07	0.343	0.632	12
Transport of inorganic cations/anions and amino acids/oligopeptides	3.07	0.241	1.177	26
Neurotransmitter release cycle	3.03	0.325	-1.387	13
cAMP-mediated signaling	3.01	0.199	-1.508	48
Interleukin-10 signaling	3.01	0.311	2.673	14
Hereditary Breast Cancer Signaling	3	0.225	0.898	31
Macrophage Alternative Activation Signaling Pathway	2.99	0.208	0.48	40
Bone Mineralization Signaling Pathway	2.99	0.222	2.828	32
Small Cell Lung Cancer Signaling	2.99	0.245	1.941	24
CXCR4 Signaling	2.98	0.214	0.928	36
Chondroitin Sulfate Biosynthesis	2.97	0.268	1.941	19
RANK Signaling in Osteoclasts	2.95	0.247	2.065	23

NOD1/2 Signaling Pathway	2.95	0.207	3.124	40
PIP3 activates AKT signaling	2.94	0.221	0.707	32
Renin-Angiotensin Signaling	2.91	0.23	1.091	28
Apelin Cardiac Fibroblast Signaling Pathway	2.89	0.391	-1.667	9
Sucrose Degradation V (Mammalian)	2.88	0.625	-1.342	5
Glyoxylate metabolism and glycine degradation	2.86	0.421	-2.828	8
GABA synthesis, release, reuptake and degradation	2.86	0.421	0	8
Thyroid Cancer Signaling	2.85	0.256	0.894	20
Aldosterone Signaling in Epithelial Cells	2.84	0.211	0.775	36
Ovarian Cancer Signaling	2.83	0.214	1.5	34
April Mediated Signaling	2.81	0.31	1.732	13
PEDF Signaling	2.81	0.25	1.213	21
p38 MAPK Signaling	2.8	0.226	2.041	28
Glucocorticoid Receptor Signaling	2.75	0.168	-	101
Interleukin-6 family signaling	2.74	0.375	1	9
Androgen Biosynthesis	2.74	0.375	-2.236	9
Cardiac Hypertrophy Signaling	2.73	0.192	1.067	50
Triglyceride metabolism	2.72	0.316	-0.577	12
Actin Nucleation by ARP-WASP Complex	2.71	0.242	1.732	22
Lysine catabolism	2.71	0.5	-2.449	6
B Cell Activating Factor Signaling	2.71	0.302	1.897	13
Uracil Degradation II (Reductive)	2.69	1	-	3
Thymine Degradation	2.69	1	-	3
Potassium Channels	2.67	0.233	0	24
Extrinsic Prothrombin Activation Pathway	2.67	0.438	-	7
Glycerophospholipid biosynthesis	2.59	0.219	0	28
Glioma Signaling	2.59	0.219	0.5	28
Endocannabinoid Developing Neuron Pathway	2.59	0.219	0.816	28
HER-2 Signaling in Breast Cancer	2.58	0.194	3.904	44
Regulation of Actin-based Motility by Rho	2.55	0.225	1.069	25
Amyotrophic Lateral Sclerosis Signaling	2.54	0.222	1.279	26
Role of NANOG in Mammalian Embryonic Stem Cell Pluripotency	2.54	0.22	0.258	27
Choline catabolism	2.5	0.667	-2	4
Lipid particle organization	2.5	0.667	-1	4

JAK/STAT Signaling	2.5	0.241	1.414	20
Metabolism of Angiotensinogen to Angiotensins	2.49	0.412	-1.89	7
Neuregulin Signaling	2.49	0.22	2.138	26
EGF Signaling	2.46	0.268	1.291	15
Estrogen-mediated S-phase Entry	2.46	0.346	1.667	9
VDR/RXR Activation	2.46	0.244	0.277	19
Transcriptional regulation by RUNX2	2.44	0.275	1.941	14
FAT10 Cancer Signaling Pathway	2.44	0.275	1.732	14
AMPK Signaling	2.42	0.189	0.557	46
Signaling by ALK	2.42	0.323	1.897	10
Sperm Motility	2.42	0.187	0.258	48
Tuberculosis Latent Signaling Pathway	2.42	0.233	-0.655	21
Dermatan Sulfate Biosynthesis	2.42	0.247	1.732	18
Kinesins	2.42	0.258	2.5	16
IL-7 Signaling Pathway	2.39	0.241	0.5	19
Role of PKR in Interferon Induction and Antiviral Response	2.39	0.21	1.964	29
Estrogen Receptor Signaling	2.38	0.173	3.048	71
Dilated Cardiomyopathy Signaling Pathway	2.35	0.205	-0.2	31
TGF-β Signaling	2.35	0.227	1.5	22
Chondroitin Sulfate Biosynthesis (Late Stages)	2.34	0.254	1.897	16
Stearate Biosynthesis I (Animals)	2.34	0.254	-1.265	16
Xenobiotic Metabolism AHR Signaling Pathway	2.34	0.22	0	24
Gαs Signaling	2.33	0.213	-0.688	27
Transcriptional Regulatory Network in Embryonic Stem Cells	2.33	0.201	0.87	33
Nuclear Cytoskeleton Signaling Pathway	2.33	0.19	3.363	42
Ketone body metabolism	2.32	0.5	-0.447	5
Epithelial Adherens Junction Signaling	2.32	0.203	1.976	32
rRNA processing	2.31	0.312	3.162	10
Extra-nuclear estrogen signaling	2.29	0.24	2.828	18
Endocannabinoid Cancer Inhibition Pathway	2.26	0.204	-1.3	30
Azathioprine ADME	2.25	0.348	0	8
NFE2L2 regulating anti-oxidant/detoxification enzymes	2.25	0.348	1.414	8
Post-translational modification: synthesis of GPI-anchored proteins	2.24	0.226	-0.218	21
Role of Pattern Recognition Receptors in Recognition of Bacteria and Viruses	2.22	0.201	2.324	31

Macropinocytosis Signaling	2.22	0.237	1.508	18
Metabolism of steroid hormones	2.21	0.289	-0.302	11
TNFR2 Signaling	2.21	0.303	1.414	10
BEX2 Signaling Pathway	2.21	0.232	-0.471	19
Role of JAK2 in Hormone-like Cytokine Signaling	2.2	0.246	-1.732	16
CCR3 Signaling in Eosinophils	2.2	0.206	1	28
Apelin Adipocyte Signaling Pathway	2.19	0.223	-0.447	21
Apoptosis Signaling	2.18	0.217	0.688	23
γ-linolenate Biosynthesis II (Animals)	2.18	0.368	-1.134	7
Bile Acid Biosynthesis, Neutral Pathway	2.18	0.368	-2	7
G alpha (q) signalling events	2.18	0.195	0.686	34
Tuberculosis Active Signaling Pathway	2.17	0.184	0	45
Multiple Sclerosis Signaling Pathway	2.17	0.187	1.718	41
TP53 Regulates Transcription of Cell Cycle Genes	2.17	0.265	3.606	13
PFKFB4 Signaling Pathway	2.17	0.265	0.905	13
Angiopoietin Signaling	2.16	0.234	0.632	18
Relaxin Signaling	2.14	0.199	0.447	31
Netrin-1 signaling	2.14	0.273	0	12
Catecholamine Biosynthesis	2.13	0.75	-	3
Interleukin-7 signaling	2.13	0.333	0	8
Fatty Acid Activation	2.13	0.4	-0.816	6
Sensory processing of sound by outer hair cells of the cochlea	2.12	0.255	-0.535	14
SUMOylation of intracellular receptors	2.11	0.31	-2.333	9
Role of p14/p19ARF in Tumor Suppression	2.11	0.31	-1.134	9
Phenylalanine and tyrosine metabolism	2.11	0.455	-2.236	5
Aspartate and asparagine metabolism	2.11	0.455	-1.342	5
NFE2L2 regulating tumorigenic genes	2.11	0.455	2.236	5
Glycine Betaine Degradation	2.11	0.455	-2.236	5
14-3-3-mediated Signaling	2.1	0.205	1.414	27
Melanoma Signaling	2.09	0.26	0.905	13
Role of MAPK Signaling in the Pathogenesis of Influenza	2.09	0.226	-	19
NAD Signaling Pathway	2.09	0.199	0.756	30
MSP-RON Signaling in Macrophages Pathway	2.09	0.208	1.877	25
Neutrophil Extracellular Trap Signaling Pathway	2.09	0.169	0.985	69

Role of MAPK Signaling in Promoting the Pathogenesis of Influenza	2.08	0.211	1.279	24
Prolactin Signaling	2.08	0.219	0.535	21
Apelin Endothelial Signaling Pathway	2.08	0.2	0.853	29
White Adipose Tissue Browning Pathway	2.07	0.201	0.2	28
CD27 Signaling in Lymphocytes	2.05	0.25	1.265	14
Cyclophilin Signaling Pathway	2.05	0.181	3.13	45
Ceramide Signaling	2.03	0.22	1	20
Mouse Embryonic Stem Cell Pluripotency	2.03	0.214	0.655	22
GABAergic Receptor Signaling Pathway (Enhanced)	2.03	0.2	-0.192	28
Gap junction trafficking and regulation	2.01	0.255	1.387	13
Gustation Pathway	2	0.185	-0.333	39
Gαi Signaling	2	0.197	-1.279	29
Sleep NREM Signaling Pathway	1.99	0.207	-0.408	24
Signaling by Retinoic Acid	1.98	0.261	-2.309	12
WNT/β-catenin Signaling	1.97	0.191	0.392	33
Platelet Adhesion to exposed collagen	1.97	0.375	0.816	6
Superpathway of Citrulline Metabolism	1.97	0.375	-1.633	6
Oxytocin Signaling Pathway	1.97	0.175	2.48	50
DAG and IP3 signaling	1.95	0.268	0.632	11
tRNA processing in the mitochondrion	1.95	0.268	3.317	11
RET signaling	1.95	0.268	0.302	11
Cholecystokinin/Gastrin-mediated Signaling	1.94	0.205	1.46	24
α-Adrenergic Signaling	1.94	0.207	0	23
Melanocyte Development and Pigmentation Signaling	1.93	0.212	1.213	21
Formation of intermediate mesoderm	1.93	0.5	0	4
NFE2L2 regulating MDR associated enzymes	1.93	0.5	1	4
Vitamin D (calciferol) metabolism	1.92	0.417	-0.447	5
Oleate Biosynthesis II (Animals)	1.92	0.417	-1.342	5
Tight Junction Signaling	1.92	0.188	-	34
Netrin Signaling	1.9	0.189	0.898	33
Metabolism of porphyrins	1.9	0.308	-1.414	8
ERB2-ERBB3 Signaling	1.9	0.234	1.387	15
Chronic Myeloid Leukemia Signaling	1.89	0.174	4.907	49
ABRA Signaling Pathway	1.87	0.216	2.065	19

Signaling by EGFR	1.87	0.245	1.387	13
Xenobiotic Metabolism General Signaling Pathway	1.87	0.19	0.816	31
Adrenomedullin signaling pathway	1.87	0.183	0.343	37
Th1 and Th2 Activation Pathway	1.87	0.188	-	33
Orexin Signaling Pathway	1.86	0.178	0.926	43
Role of NFAT in Cardiac Hypertrophy	1.85	0.179	1	41
Detoxification of Reactive Oxygen Species	1.84	0.27	1.265	10
Neuropathic Pain Signaling in Dorsal Horn Neurons	1.83	0.208	-0.218	21
Mitochondrial Dysfunction	1.82	0.168	-0.687	58
Surfactant metabolism	1.81	0.281	0.333	9
Signaling by TGF-beta Receptor Complex	1.8	0.241	2.496	13
UVB-Induced MAPK Signaling	1.8	0.241	1.155	13
Effects of PIP2 hydrolysis	1.8	0.296	0.707	8
BMAL1:CLOCK,NPAS2 activates circadian gene expression	1.8	0.296	0.707	8
Integrin signaling	1.8	0.296	0.707	8
GABA Receptor Signaling	1.8	0.195	-	26
Glycine Degradation (Creatine Biosynthesis)	1.79	1	-	2
Glutamine Degradation I	1.79	1	-	2
Integration of energy metabolism	1.79	0.204	1.706	22
PPAR Signaling	1.79	0.204	-1.342	22
Transport of vitamins, nucleosides, and related molecules	1.79	0.256	-0.302	11
Melanin biosynthesis	1.78	0.6	-	3
Intestinal absorption	1.78	0.6	-	3
Lysine Degradation II	1.78	0.6	-	3
Lysine Degradation V	1.78	0.6	-	3
Tyrosine Degradation I	1.78	0.6	-	3
Renal Cell Carcinoma Signaling	1.77	0.218	0.905	17
iNOS Signaling	1.76	0.245	2.53	12
SUMOylation of immune response proteins	1.76	0.385	0.447	5
Autophagy	1.75	0.178	0.324	39
Death Receptor Signaling	1.73	0.206	0.243	20
IL-15 Production	1.73	0.197	1.46	24
PI3K Cascade	1.72	0.25	0.302	11
TAK1-dependent IKK and NF-kappa-B activation	1.72	0.25	0.905	11

Assembly and cell surface presentation of NMDA receptors	1.72	0.25	0.905	11
Interleukin-1 processing	1.72	0.444	2	4
Sphingosine and Sphingosine-1-phosphate Metabolism	1.72	0.444	-1	4
Caveolar-mediated Endocytosis Signaling	1.72	0.215	3.207	17
Sphingolipid metabolism	1.71	0.2	1.667	22
Interleukin-1 family signaling	1.7	0.194	1.043	25
Clathrin-mediated endocytosis	1.7	0.194	0.6	25
Endothelin-1 Signaling	1.7	0.18	0.557	35
MYC Mediated Apoptosis Signaling	1.69	0.24	-1.508	12
Retinoate Biosynthesis I	1.69	0.24	-2	12
Nephrin family interactions	1.69	0.304	1.134	7
MAP kinase activation	1.68	0.256	0.632	10
Acute Myeloid Leukemia Signaling	1.68	0.207	0.775	19
Antioxidant Action of Vitamin C	1.68	0.197	-0.577	23
Cyclins and Cell Cycle Regulation	1.67	0.209	0	18
Cargo recognition for clathrin-mediated endocytosis	1.65	0.2	0.218	21
Neurexins and neuroligins	1.62	0.228	-1.387	13
Carnitine metabolism	1.61	0.357	-0.447	5
Glutamate and glutamine metabolism	1.61	0.357	-0.447	5
Glycolysis I	1.61	0.276	-0.378	8
Transcriptional Regulation by MECP2	1.61	0.222	-1.604	14
Sensory processing of sound by inner hair cells of the cochlea	1.61	0.217	0.258	15
Cell Cycle: G1/S Checkpoint Regulation	1.61	0.217	-2.111	15
Protein Kinase A Signaling	1.6	0.162	1.508	64
Sphingosine-1-phosphate Signaling	1.6	0.193	1.342	23
Tryptophan Degradation III (Eukaryotic)	1.59	0.292	-2.449	7
G alpha (s) signalling events	1.59	0.186	-0.192	27
Calcium Signaling	1.59	0.174	0.2	38
Mitochondrial L-carnitine Shuttle Pathway	1.58	0.316	-0.816	6
Acetylcholine Receptor Signaling Pathway	1.58	0.177	0.169	35
GPVI-mediated activation cascade	1.56	0.257	1	9
Semaphorin interactions	1.55	0.219	2.138	14
Peroxisomal protein import	1.55	0.219	-3.742	14
Caspase activation via Dependence Receptors in the absence of ligand	1.54	0.4	1	4

Glycogen Degradation II	1.54	0.4	-	4
Citrulline Biosynthesis	1.54	0.4	-1	4
FOXO-mediated transcription of oxidative stress, metabolic and neuronal genes	1.53	0.267	-1.414	8
NFE2L2 regulating inflammation associated genes	1.52	0.5	-	3
Arginine Biosynthesis IV	1.52	0.5	-	3
Chondroitin and Dermatan Biosynthesis	1.52	0.5	-	3
Tryptophan Degradation to 2-amino-3-carboxymuconate Semialdehyde	1.52	0.5	-	3
Basal Cell Carcinoma Signaling	1.51	0.211	0.333	15
Semaphorin Signaling in Neurons	1.5	0.22	-	13
Oxytocin in Brain Signaling Pathway	1.49	0.174	0.174	35
BMP signaling pathway	1.49	0.2	0.258	18
Macrophage Classical Activation Signaling Pathway	1.48	0.176	3.889	33
Receptor-type tyrosine-protein phosphatases	1.48	0.3	-2.449	6
Synthesis, secretion, and deacylation of Ghrelin	1.48	0.3	-0.816	6
Valine Degradation I	1.48	0.3	-1.633	6
Glucocorticoid Biosynthesis	1.48	0.3	-	6
Apelin Liver Signaling Pathway	1.48	0.3	0.816	6
Circadian Rhythm Signaling	1.47	0.167	-	45
Insulin Receptor Signaling	1.46	0.186	0.853	24
Induction of Apoptosis by HIV1	1.45	0.212	1.155	14
Oxidative Ethanol Degradation III	1.45	0.212	-1	14
Dermatan Sulfate Biosynthesis (Late Stages)	1.45	0.217	1.134	13
Natural Killer Cell Signaling	1.44	0.172	2.263	35
Neuroprotective Role of THOP1 in Alzheimer's Disease	1.43	0.185	0	24
Growth Hormone Signaling	1.41	0.205	-0.277	15
Pregnenolone Biosynthesis	1.41	0.269	-	7
Ethanol Degradation IV	1.41	0.269	-0.816	7
Abacavir ADME	1.4	0.209	-0.535	14
Intrinsic Pathway for Apoptosis	1.39	0.218	2.887	12
Signaling by PTK6	1.39	0.218	1.732	12
Regulation of the Epithelial Mesenchymal Transition in Development Pathway	1.39	0.198	2.324	17
Branched-chain amino acid catabolism	1.38	0.286	-1.633	6
RIPK1-mediated regulated necrosis	1.37	0.25	0.707	8
ERK5 Signaling	1.36	0.203	3.051	15

RHO GTPases regulate CFTR trafficking	1.35	0.667	-	2
Interconversion of 2-oxoglutarate and 2-hydroxyglutarate	1.35	0.667	-	2
Serine Biosynthesis	1.35	0.667	-	2
Tyrosine Biosynthesis IV	1.35	0.667	-	2
Cell Cycle Regulation by BTG Family Proteins	1.35	0.237	1.89	9
TWEAK Signaling	1.35	0.237	1.134	9
MSP-RON Signaling Pathway	1.34	0.21	-	13
Thyroid Hormone Metabolism II (via Conjugation and/or Degradation)	1.34	0.21	-1.134	13
Signaling by ERBB2	1.34	0.22	1.508	11
Signaling by TGFBR3	1.34	0.22	2.111	11
Chemokine Signaling	1.33	0.198	0.277	16
fMLP Signaling in Neutrophils	1.33	0.18	1.698	24
Histidine Degradation VI	1.33	0.259	-1	7
Erythrocytes take up oxygen and release carbon dioxide	1.32	0.429	-	3
Ceramide Degradation	1.32	0.429	-	3
Heparan Sulfate Biosynthesis	1.3	0.188	0.905	19
Sumoylation Pathway	1.3	0.188	-0.258	19
Airway Inflammation in Asthma	1.3	0.242	2.236	8
Inhibition of Angiogenesis by TSP1	1.3	0.242	0.816	8
IL-27 Signaling Pathway	1.3	0.179	2.041	24

Table S8: The top canonical pathways enriched in AH/ASH by DEG analysis

**Ingenuity Canonical Pathways**	**-log(p-value)**	**Ratio**	**z-score**	**Gene count**
Fcgamma receptor (FCGR) dependent phagocytosis	10.4	0.11	4.243	18
Complement cascade	9.77	0.118	3	16
Binding and Uptake of Ligands by Scavenger Receptors	8.87	0.123	3.207	14
Immunoregulatory interactions between a Lymphoid and a non-Lymphoid cell	7.67	0.0791	3.638	17
Fc epsilon receptor (FCERI) signaling	5.63	0.068	3.742	14
Cell surface interactions at the vascular wall	5.44	0.0654	3.742	14
Mitochondrial Fatty Acid Beta-Oxidation	4.83	0.162	-1.633	6
Stearate Biosynthesis I (Animals)	4.43	0.111	-	7
Signaling by the B Cell Receptor (BCR)	4.36	0.0647	3.317	11
Remodeling of Epithelial Adherens Junctions	4.09	0.0986	-	7
RHO GTPases activate IQGAPs	4.02	0.156	2.236	5
Fatty Acid β-oxidation I	3.89	0.147	-	5
eNOS Signaling	3.86	0.0621	1.342	10
Response to elevated platelet cytosolic Ca2+	3.84	0.0682	1	9
Transport of glycerol from adipocytes to the liver by Aquaporins	3.67	1	-	2
Inhibition of Matrix Metalloproteases	3.66	0.132	-0.447	5
IL-15 Signaling	3.5	0.0361	-	19
Leukocyte Extravasation Signaling	3.22	0.0515	1.414	10
Translocation of SLC2A4 (GLUT4) to the plasma membrane	3.19	0.0833	1.633	6
LXR/RXR Activation	3.17	0.0615	-2.449	8
Paracetamol ADME	3.1	0.138	-1	4
Gap junction trafficking and regulation	3.05	0.098	2.236	5
FcγRIIB Signaling in B Lymphocytes	3.02	0.0338	-	18
p70S6K Signaling	2.99	0.0327	-	19
HIF1α Signaling	2.95	0.0476	1.667	10
Phase II - Conjugation of compounds	2.95	0.0642	-1.134	7
DHCR24 Signaling Pathway	2.9	0.0559	-1.134	8
NAD Biosynthesis III	2.9	0.5	-	2
PI3K Signaling in B Lymphocytes	2.9	0.0321	-	19
HSP90 chaperone cycle for steroid hormone receptors in the presence of ligand	2.83	0.0877	2.236	5

Xenobiotic Metabolism PXR Signaling Pathway	2.79	0.0452	-1.134	10
Intestinal absorption	2.68	0.4	-	2
Chondroitin Sulfate Biosynthesis (Late Stages)	2.64	0.0794	-	5
Xenobiotic Metabolism CAR Signaling Pathway	2.62	0.0429	0.378	10
Semaphorin interactions	2.61	0.0781	2.236	5
B Cell Development	2.59	0.0326	-	16
Systemic Lupus Erythematosus in B Cell Signaling Pathway	2.58	0.0288	2.236	21
Human Embryonic Stem Cell Pluripotency	2.55	0.045	1.667	9
B Cell Receptor Signaling	2.53	0.0298	-	19
Pulmonary Healing Signaling Pathway	2.53	0.0448	2.333	9
Germ Cell-Sertoli Cell Junction Signaling	2.42	0.0468	-	8
Chondroitin Sulfate Biosynthesis	2.41	0.0704	-	5
Aggrephagy	2.41	0.0909	2	4
Chaperone Mediated Autophagy	2.41	0.136	-	3
Dermatan Sulfate Biosynthesis	2.36	0.0685	-	5
RAF-independent MAPK1/3 activation	2.35	0.13	-	3
Neutrophil degranulation	2.32	0.0314	2.84	15
Tryptophan Degradation III (Eukaryotic)	2.3	0.125	-	3
Tumor Microenvironment Pathway	2.29	0.0444	1.89	8
NF-κB Activation by Viruses	2.24	0.0641	2	5
Maturity Onset Diabetes of Young (MODY) Signaling	2.24	0.0641	-	5
RHO GTPase cycle	2.16	0.0311	3.742	14
NAD Signaling Pathway	2.16	0.0464	-1.134	7
Lipophagy	2.14	0.222	-	2
Neuregulin Signaling	2.1	0.0508	-	6
Integrin cell surface interactions	2.08	0.0588	0.447	5
Activation of NMDA receptors and postsynaptic events	2.08	0.0588	1.342	5
Phospholipase C Signaling	2.08	0.024	2.828	27
Osteoarthritis Pathway	2.06	0.0378	0.816	9
TRIM21 Intracellular Antibody Signaling Pathway	2.01	0.0282	4	16
L1CAM interactions	2	0.0484	2.449	6
NAP1L1 Transcription Regulation Signaling Pathway	2	0.0562	2.236	5
Regulation of Cellular Mechanics by Calpain Protease	2	0.0562	-	5
Xenobiotic Metabolism Signaling	2	0.0332	-	11

Triacylglycerol Biosynthesis	1.99	0.069	-	4
Th1 Pathway	1.99	0.048	2.236	6
Granulocyte Adhesion and Diapedesis	1.97	0.0392	-	8
TCR signaling	1.97	0.0476	2.449	6
Aspartate and asparagine metabolism	1.97	0.182	-	2
NAD biosynthesis II (from tryptophan)	1.97	0.182	-	2
Cell junction organization	1.94	0.0543	2.236	5
Dermatan Sulfate Biosynthesis (Late Stages)	1.94	0.0667	-	4
Reversible hydration of carbon dioxide	1.89	0.167	-	2
Lysine catabolism	1.89	0.167	-	2
Oleate Biosynthesis II (Animals)	1.89	0.167	-	2
LPS/IL-1 Mediated Inhibition of RXR Function	1.89	0.0336	-	10
Kinesins	1.89	0.0645	2	4
Primary Immunodeficiency Signaling	1.89	0.0645	-	4
Aldosterone Signaling in Epithelial Cells	1.88	0.0409	-	7
Hematoma Resolution Signaling Pathway	1.84	0.0347	0.707	9
Methylthiopropionate Biosynthesis	1.83	1	-	1
Methylglyoxal Degradation VI	1.83	1	-	1
Adenine and Adenosine Salvage VI	1.83	1	-	1
Passive transport by Aquaporins	1.83	0.154	-	2
Nuclear Cytoskeleton Signaling Pathway	1.78	0.0362	2.828	8
Acetylcholine binding and downstream events	1.76	0.143	-	2
Agranulocyte Adhesion and Diapedesis	1.75	0.0357	-	8
FLT3 Signaling	1.72	0.0769	-	3
Fatty Acid Activation	1.7	0.133	-	2
Paxillin Signaling	1.7	0.0472	2	5
Role of NFAT in Regulation of the Immune Response	1.68	0.0229	2.646	24
Axonal Guidance Signaling	1.68	0.0271	-	14
Mechanisms of Viral Exit from Host Cells	1.66	0.0732	-	3
Hepatic Fibrosis / Hepatic Stellate Cell Activation	1.63	0.0365	-	7
DAP12 interactions	1.6	0.0698	-	3
Glutaryl-CoA Degradation	1.6	0.118	-	2
Interferon alpha/beta signaling	1.6	0.0526	1	4
Pathogen Induced Cytokine Storm Signaling Pathway	1.59	0.0288	0.707	11

Assembly and cell surface presentation of NMDA receptors	1.58	0.0682	-	3
Aspirin ADME	1.58	0.0682	-	3
IL-12 Signaling and Production in Macrophages	1.57	0.0331	1.633	8
Carboxyterminal post-translational modifications of tubulin	1.55	0.0667	-	3
Mitotic G2-G2/M phases	1.55	0.035	2.646	7
Asparagine Degradation I	1.54	0.5	-	1
β-alanine Degradation I	1.54	0.5	-	1
Glutamate Biosynthesis II	1.54	0.5	-	1
Glutamate Degradation X	1.54	0.5	-	1
Epithelial Adherens Junction Signaling	1.54	0.038	-0.816	6
Sertoli Cell-Sertoli Cell Junction Signaling	1.53	0.0324	2.828	8
Natural Killer Cell Signaling	1.52	0.0345	2.449	7
γ-linolenate Biosynthesis II (Animals)	1.51	0.105	-	2
Mitochondrial L-carnitine Shuttle Pathway	1.51	0.105	-	2
Sheddase Signaling Pathway	1.51	0.0343	0.378	7
Role of JAK family kinases in IL-6-type Cytokine Signaling	1.51	0.0494	1	4
Interleukin-2 family signaling	1.5	0.0638	-	3
IL-15 Production	1.47	0.041	2.236	5
Integrin Signaling	1.45	0.0333	2.449	7
Sperm Motility	1.44	0.0311	-	8
RAF/MAP kinase cascade	1.4	0.0305	2.121	8
Glycosaminoglycan metabolism	1.4	0.0391	2.236	5
MHC class II antigen presentation	1.4	0.0391	2.236	5
GP6 Signaling Pathway	1.4	0.0391	1	5
Cellular hexose transport	1.39	0.0909	-	2
Regulation of TLR by endogenous ligand	1.39	0.0909	-	2
Clathrin-mediated endocytosis	1.38	0.0388	0.447	5
RHO GTPases regulate CFTR trafficking	1.36	0.333	-	1
Pyrophosphate hydrolysis	1.36	0.333	-	1
Uracil Degradation II (Reductive)	1.36	0.333	-	1
Thymine Degradation	1.36	0.333	-	1
Anandamide Degradation	1.36	0.333	-	1
Nephrin family interactions	1.36	0.087	-	2
Nucleotide salvage	1.36	0.087	-	2

Th1 and Th2 Activation Pathway	1.34	0.0341	-	6
NLR signaling pathways	1.31	0.0536	-	3
Heparan Sulfate Biosynthesis (Late Stages)	1.31	0.0426	-	4
Molecular Mechanisms of Cancer	1.3	0.0221	3.771	19

Table S9: The top canonical pathways enriched in AH/ASH by DSD analysis

Ingenuity Canonical Pathways	-log(p-value)	Ratio	z-score	Gene count
CREB Signaling in Neurons	6.15	0.0325	-3.9	20
BBSome Signaling Pathway	5.65	0.0345	-3.5	17
Lung Ionic Balance Signaling Pathway	5.32	0.0326	-3.638	17
S100 Family Signaling Pathway	5.13	0.0269	-4.025	21
Breast Cancer Regulation by Stathmin1	5.05	0.0297	-3.638	18
Cellular Effects of Sildenafil (Viagra)	4.35	0.0263	2.828	18
Phagosome Formation	4.18	0.0255	-3.638	18
G-Protein Coupled Receptor Signaling	4.13	0.0252	-3.638	18
Molecular Mechanisms of Cancer	4.03	0.0232	-3.638	20
Melanin biosynthesis	3.11	0.4	-	2
Class A/1 (Rhodopsin-like receptors)	3.09	0.0301	-3.162	10
Neurotransmitter clearance	2.47	0.2	-	2
Oleate Biosynthesis II (Animals)	2.31	0.167	-	2
FAK Signaling	2.27	0.0174	-3.638	18
Cardiac conduction	2.21	0.0385	-2.236	5
Neuroprotective Role of THOP1 in Alzheimer's Disease	2.21	0.0385	-1	5
TR/RXR Activation	2.2	0.0382	-0.447	5
Platelet homeostasis	2.14	0.0465	-1	4
G alpha (i) signalling events	2.07	0.0271	-1.89	7
Lanosterol Biosynthesis	2.05	1	-	1
Gαi Signaling	1.99	0.034	-1	5
Metabolism of amine-derived hormones	1.96	0.111	-	2
γ-linolenate Biosynthesis II (Animals)	1.92	0.105	-	2
Potassium Channels	1.87	0.0388	-1	4
Cellular hexose transport	1.79	0.0909	-	2
L-dopachrome Biosynthesis	1.75	0.5	-	1
G alpha (q) signalling events	1.7	0.0287	-2.236	5
cAMP-mediated signaling	1.68	0.0249	-1.342	6
Cholesterol biosynthesis	1.62	0.0741	-	2
Serotonin Receptor Signaling	1.6	0.0191	-1.667	9

NFE2L2 regulating TCA cycle genes	1.58	0.333	-	1
Thyronamine and Iodothyronamine Metabolism	1.58	0.333	-	1
Thyroid Hormone Metabolism I (via Deiodination)	1.58	0.333	-	1
Superpathway of Cholesterol Biosynthesis	1.51	0.0645	-	2
Circadian Rhythm Signaling	1.47	0.0222	-	6
Eumelanin Biosynthesis	1.46	0.25	-	1
Degradation of the extracellular matrix	1.45	0.037	-	3
Serotonin and Melatonin Biosynthesis	1.36	0.2	-	1
Citrulline-Nitric Oxide Cycle	1.36	0.2	-	1
Tuberculosis Latent Signaling Pathway	1.34	0.0333	-	3
Neurotransmitter release cycle	1.31	0.05	-	2

Table S10: The top common pathways enriched in AH/ASH by DEG and DSD analysis

Ingenuity Canonical Pathways	-log(p-value)	Ratio	z-score	Gene count
Extracellular matrix organization	19.4	0.421	6.112	45
Phase I - Functionalization of compounds	16.6	0.381	-4.529	43
Hepatic Fibrosis / Hepatic Stellate Cell Activation	16	0.297	-	57
Collagen degradation	15.7	0.484	4.49	31
Assembly of collagen fibrils and other multimeric structures	15.5	0.492	5.112	30
Sheddase Signaling Pathway	15.3	0.284	2.364	58
Pulmonary Fibrosis Idiopathic Signaling Pathway	15	0.236	6.437	77
Wound Healing Signaling Pathway	15	0.261	4.5	65
Integrin cell surface interactions	14.1	0.4	5.488	34
S100 Family Signaling Pathway	12.8	0.17	5.945	133
Interleukin-4 and Interleukin-13 signaling	11.6	0.324	4	36
Role of Osteoclasts in Rheumatoid Arthritis Signaling Pathway	10.8	0.211	4.202	67
Post-translational protein phosphorylation	10.7	0.318	3.43	34
Elastic fibre formation	10.7	0.477	4.146	21
Regulation of Insulin-like Growth Factor (IGF) transport and uptake by IGFBPs	10.7	0.298	3.452	37
Molecular Mechanisms of Cancer	10.1	0.156	5.371	134
Tumor Microenvironment Pathway	9.94	0.25	4.841	45
Collagen chain trimerization	9.74	0.455	3.578	20
Collagen biosynthesis and modifying enzymes	8.96	0.358	3.674	24
LPS/IL-1 Mediated Inhibition of RXR Function	8.88	0.201	1.387	60
Atherosclerosis Signaling	8.82	0.265	2.191	36
Degradation of the extracellular matrix	8.46	0.321	4.315	26
Hepatic Fibrosis Signaling Pathway	8.26	0.178	4.131	74
Osteoarthritis Pathway	8.15	0.21	1.183	50
Coagulation System	7.96	0.457	-0.258	16
Syndecan interactions	7.93	0.519	3.207	14
IL-17A Signaling in Fibroblasts	7.9	0.312	3.545	25
LXR/RXR Activation	7.66	0.254	-2.353	33
Cellular Effects of Sildenafil (Viagra)	7.42	0.152	-1.8	104
Estrogen Biosynthesis	7.4	0.383	0	18

Axonal Guidance Signaling	7.4	0.162	-	84
FXR/RXR Activation	7.28	0.215	-0.324	42
Signaling by PDGF	7.25	0.345	4.025	20
RAR Activation	7.17	0.169	-0.12	73
Bupropion Degradation	7.16	0.5	0.447	13
ABRA Signaling Pathway	6.98	0.284	4.2	25
Bile acid and bile salt metabolism	6.92	0.378	-2.183	17
Arachidonic acid metabolism	6.92	0.378	-1.5	17
Binding and Uptake of Ligands by Scavenger Receptors	6.83	0.254	2.785	29
ILK Signaling	6.7	0.208	4.004	41
Nicotine Degradation II	6.68	0.282	-0.333	24
Irritable Bowel Syndrome Signaling Pathway	6.54	0.183	2.885	53
G-Protein Coupled Receptor Signaling	6.54	0.146	2.312	104
Phagosome Formation	6.49	0.146	4.724	103
Inhibition of Matrix Metalloproteases	6.47	0.395	-1.155	15
Xenobiotic Metabolism Signaling	6.4	0.175	-	58
Agranulocyte Adhesion and Diapedesis	6.39	0.196	-	44
Glycation Signaling Pathway	6.33	0.196	3.92	44
Formation of Fibrin Clot (Clotting Cascade)	6.3	0.385	0.775	15
Airway Pathology in Chronic Obstructive Pulmonary Disease	6.28	0.246	1.89	28
Transport of bile salts and organic acids, metal ions and amine compounds	6.16	0.274	-0.209	23
Sertoli Cell-Germ Cell Junction Signaling Pathway (Enhanced)	6.14	0.191	-0.447	45
Neuroinflammation Signaling Pathway	6.09	0.173	3.244	57
Cardiac Hypertrophy Signaling (Enhanced)	6.09	0.153	3.75	82
Role of Chondrocytes in Rheumatoid Arthritis Signaling Pathway	6.03	0.222	3.651	32
Ethanol Degradation II	5.99	0.389	-1.897	14
Pathogen Induced Cytokine Storm Signaling Pathway	5.94	0.165	4.557	63
Xenobiotic Metabolism CAR Signaling Pathway	5.9	0.189	0.2	44
O-linked glycosylation	5.83	0.239	4.426	27
Serotonin Receptor Signaling	5.77	0.155	2.926	73
Hematoma Resolution Signaling Pathway	5.75	0.181	-2.714	47
Class A/1 (Rhodopsin-like receptors)	5.67	0.169	3.742	56
Leukocyte Extravasation Signaling	5.58	0.196	3.157	38
Sertoli Cell-Sertoli Cell Junction Signaling	5.58	0.182	3.13	45

Aspirin ADME	5.52	0.341	-3.357	15
Retinoid metabolism and transport	5.52	0.341	0.775	15
Dissolution of Fibrin Clot	5.49	0.615	2.828	8
Granulocyte Adhesion and Diapedesis	5.44	0.191	-	39
Formation of the ureteric bud	5.38	0.476	0.707	10
Preeclampsia Signaling Pathway	5.37	0.205	2.611	33
Nicotine Degradation III	5.25	0.267	0	20
NCAM signaling for neurite out-growth	5.25	0.286	2.828	18
GP6 Signaling Pathway	5.22	0.219	4.536	28
Acetone Degradation I (to Methylglyoxal)	5.2	0.341	0.447	14
Lung Ionic Balance Signaling Pathway	5.19	0.148	2.393	77
Regulation of the Epithelial Mesenchymal Transition by Growth Factors Pathway	5.17	0.191	4.382	37
DHCR24 Signaling Pathway	5.16	0.21	-1.671	30
HIF1α Signaling	5.13	0.186	2	39
Regulation of Actin-based Motility by Rho	4.96	0.225	2.668	25
Superpathway of Melatonin Degradation	4.96	0.242	0.707	22
Response to elevated platelet cytosolic Ca2+	4.95	0.212	2.646	28
Smooth Muscle Contraction	4.93	0.326	3.207	14
Melatonin Degradation I	4.92	0.247	0.378	21
Activation of Matrix Metalloproteinases	4.86	0.364	2.309	12
Colorectal Cancer Metastasis Signaling	4.85	0.17	3.124	46
Neurovascular Coupling Signaling Pathway	4.81	0.177	1.298	41
Regulation of the Epithelial-Mesenchymal Transition Pathway	4.78	0.186	-	36
Hepatic Cholestasis	4.78	0.178	3.042	40
Glycosaminoglycan metabolism	4.75	0.211	2.2	27
Noradrenaline and Adrenaline Degradation	4.74	0.333	-1.667	13
Role of JAK family kinases in IL-6-type Cytokine Signaling	4.71	0.247	1.886	20
Bone Mineralization Signaling Pathway	4.65	0.201	2.785	29
RHOGDI Signaling	4.64	0.177	-3.4	39
CDX Gastrointestinal Cancer Signaling Pathway	4.63	0.183	-1.715	36
Xenobiotic Metabolism PXR Signaling Pathway	4.6	0.176	0.209	39
Breast Cancer Regulation by Stathmin1	4.57	0.139	4.472	84
Aryl Hydrocarbon Receptor Signaling	4.56	0.186	0	34
IL-10 Signaling	4.44	0.194	0.186	30

Pulmonary Healing Signaling Pathway	4.43	0.179	3.773	36
CREB Signaling in Neurons	4.31	0.136	3.85	84
HOTAIR Regulatory Pathway	4.31	0.188	3.402	31
Role of Macrophages, Fibroblasts and Endothelial Cells in Rheumatoid Arthritis	4.22	0.154	2.188	52
Phase II - Conjugation of compounds	4.15	0.211	-1.46	23
Serotonin Degradation	4.15	0.221	-1.508	21
Role of Osteoblasts in Rheumatoid Arthritis Signaling Pathway	4.12	0.167	3.042	40
PI3K/AKT Signaling	4.12	0.175	2.183	35
Role of Tissue Factor in Cancer	4.11	0.173	4.564	36
Chondroitin Sulfate Biosynthesis (Late Stages)	4.05	0.254	2.111	16
Ephrin Receptor Signaling	4.03	0.173	1.213	35
Signaling by Rho Family GTPases	4.03	0.161	4.49	43
Integrin Signaling	4.03	0.171	4.017	36
Fructose metabolism	4.01	0.714	-1.342	5
Activin Inhibin Signaling Pathway	3.94	0.17	4	36
Glioma Invasiveness Signaling	3.92	0.239	1.291	17
Chondroitin Sulfate Biosynthesis	3.92	0.239	1.732	17
Interleukin-6 family signaling	3.9	0.375	1.667	9
Cell junction organization	3.87	0.217	3.9	20
Dilated Cardiomyopathy Signaling Pathway	3.86	0.185	-1.342	28
WNT/SHH Axonal Guidance Signaling Pathway	3.86	0.185	1.89	28
Adrenergic Receptor Signaling Pathway (Enhanced)	3.85	0.172	-1.715	34
Signaling by MET	3.82	0.228	3.771	18
HMGB1 Signaling	3.79	0.181	2.982	29
Dermatan Sulfate Biosynthesis	3.76	0.233	1.732	17
Actin Cytoskeleton Signaling	3.72	0.161	3.13	39
IL-12 Signaling and Production in Macrophages	3.72	0.161	0.164	39
Signaling by ALK	3.64	0.323	3.162	10
Platelet Adhesion to exposed collagen	3.62	0.438	1.89	7
Sucrose Degradation V (Mammalian)	3.61	0.625	-1.342	5
Superoxide Radicals Degradation	3.61	0.625	1.342	5
PAK Signaling	3.6	0.195	3.873	23
Regulation of Cellular Mechanics by Calpain Protease	3.6	0.213	2.333	19
GP1b-IX-V activation signalling	3.56	0.5	2.449	6

Bladder Cancer Signaling	3.55	0.193	2.449	23
Cell surface interactions at the vascular wall	3.53	0.164	5.24	35
Retinoate Biosynthesis I	3.5	0.26	0	13
Immunoregulatory interactions between a Lymphoid and a non-Lymphoid cell	3.49	0.163	4.802	35
MSP-RON Signaling in Cancer Cells Pathway	3.49	0.182	3.266	26
Clathrin-mediated Endocytosis Signaling	3.4	0.163	-	34
Maturity Onset Diabetes of Young (MODY) Signaling	3.39	0.218	-	17
HEY1 Signaling Pathway	3.38	0.174	2.117	28
ID1 Signaling Pathway	3.35	0.163	2.335	33
Transport of inorganic cations/anions and amino acids/oligopeptides	3.33	0.194	1.091	21
Caveolar-mediated Endocytosis Signaling	3.33	0.215	3.742	17
NRF2-mediated Oxidative Stress Response	3.32	0.152	0	41
Platelet Aggregation (Plug Formation)	3.32	0.462	1.633	6
Atorvastatin ADME	3.3	0.556	-2.236	5
Apelin Adipocyte Signaling Pathway	3.28	0.202	0.775	19
Apelin Cardiac Fibroblast Signaling Pathway	3.26	0.348	-1.89	8
Metabolism of amine-derived hormones	3.25	0.389	-1.134	7
IL-17 Signaling	3.22	0.164	4.158	31
Dermatan Sulfate Biosynthesis (Late Stages)	3.21	0.233	1.897	14
Paracetamol ADME	3.19	0.31	-0.333	9
Acute Phase Response Signaling	3.14	0.162	2.065	31
Ion channel transport	3.13	0.164	2.921	30
IL-8 Signaling	3.12	0.159	4.271	33
Agrin Interactions at Neuromuscular Junction	3.12	0.221	2.53	15
Role of IL-17A in Psoriasis	3.11	0.429	1.342	6
Docosahexaenoic Acid (DHA) Signaling	3.1	0.151	-0.333	38
Production of Nitric Oxide and Reactive Oxygen Species in Macrophages	3.1	0.161	1.134	31
Choline catabolism	3.1	0.667	-2	4
Trehalose Degradation II (Trehalase)	3.1	0.667	-	4
GABA synthesis, release, reuptake and degradation	3.08	0.368	0.378	7
Bile Acid Biosynthesis, Neutral Pathway	3.08	0.368	-2.236	7
Complement cascade	3.07	0.176	2.041	24
Prednisone ADME	3.03	0.5	-2.236	5
Actin Nucleation by ARP-WASP Complex	3.03	0.198	1.667	18

G alpha (s) signalling events	3.02	0.172	1.8	25
Cachexia Signaling Pathway	3.02	0.139	3.286	51
Role of Osteoblasts, Osteoclasts and Chondrocytes in Rheumatoid Arthritis	2.98	0.153	-	35
Detoxification of Reactive Oxygen Species	2.96	0.27	2.53	10
Glioblastoma Multiforme Signaling	2.95	0.164	2.294	28
EPH-Ephrin signaling	2.91	0.194	3.3	18
GABAergic Receptor Signaling Pathway (Enhanced)	2.89	0.171	0.209	24
Regulation of the Epithelial Mesenchymal Transition in Development Pathway	2.89	0.198	2.138	17
G alpha (i) signalling events	2.87	0.147	3.569	38
WNT/β-catenin Signaling	2.87	0.162	-0.392	28
Xenobiotic Metabolism AHR Signaling Pathway	2.87	0.183	0	20
Ethanol Degradation IV	2.86	0.308	-0.707	8
GABA Receptor Signaling	2.85	0.173	-	23
NFE2L2 regulating tumorigenic genes	2.8	0.455	2.236	5
Oxidative Ethanol Degradation III	2.78	0.212	-0.816	14
Calcium Signaling	2.77	0.151	1.807	33
Role of IL-17F in Allergic Inflammatory Airway Diseases	2.73	0.239	2.449	11
PXR/RXR Activation	2.71	0.209	-0.816	14
Glutaminergic Receptor Signaling Pathway (Enhanced)	2.7	0.138	2.111	45
IL-6 Signaling	2.67	0.171	1.606	22
Extra-nuclear estrogen signaling	2.66	0.2	3.357	15
Signaling by TGF-beta Receptor Complex	2.63	0.222	3.464	12
Cardiac conduction	2.63	0.169	1.291	22
STAT3 Pathway	2.59	0.165	2.496	23
Neurotransmitter uptake and metabolism In glial cells	2.59	0.75	-	3
Catecholamine Biosynthesis	2.59	0.75	-	3
NAFLD Signaling Pathway	2.58	0.147	2.959	33
Sensory processing of sound by outer hair cells of the cochlea	2.56	0.218	0.577	12
FAK Signaling	2.56	0.114	5.199	118
Glycolysis I	2.53	0.276	0	8
Heparan Sulfate Biosynthesis	2.5	0.178	1.732	18
VDR/RXR Activation	2.49	0.192	0.333	15
Heparan Sulfate Biosynthesis (Late Stages)	2.46	0.181	2.111	17
Formation of the nephric duct	2.45	0.333	0.816	6

Transport of vitamins, nucleosides, and related molecules	2.43	0.233	0	10
Intrinsic Prothrombin Activation Pathway	2.43	0.233	1	10
Androgen Biosynthesis	2.42	0.292	-2	7
Glycerophospholipid biosynthesis	2.37	0.164	0.218	21
Role of IL-17A in Arthritis	2.36	0.207	-	12
Oxytocin Signaling Pathway	2.36	0.137	4.542	39
Netrin-1 signaling	2.36	0.227	1.897	10
Transcriptional regulation by RUNX2	2.35	0.216	0.333	11
RAC Signaling	2.34	0.161	3.606	22
Germ Cell-Sertoli Cell Junction Signaling	2.33	0.152	-	26
Glyoxylate metabolism and glycine degradation	2.32	0.316	-2.449	6
BBSome Signaling Pathway	2.3	0.124	2.994	61
Semaphorin Signaling in Neurons	2.3	0.203	-	12
Interleukin-10 signaling	2.28	0.222	3.162	10
Cardiac Hypertrophy Signaling	2.28	0.138	1.732	36
Parkinson's Signaling Pathway	2.27	0.133	1.093	42
Paxillin Signaling	2.27	0.17	2.714	18
RAF/MAP kinase cascade	2.25	0.137	4	36
PCP (Planar Cell Polarity) Pathway	2.24	0.2	1.732	12
Taurine Biosynthesis	2.22	0.6	-	3
IL-17A Signaling in Airway Cells	2.21	0.191	1.89	13
IL-17A Signaling in Gastric Cells	2.21	0.269	1.633	7
Eicosanoid Signaling	2.2	0.135	3.244	38
Plasma lipoprotein assembly, remodeling, and clearance	2.19	0.184	-1.604	14
ERK/MAPK Signaling	2.17	0.141	1.706	31
Airway Inflammation in Asthma	2.16	0.242	2	8
CXCR4 Signaling	2.15	0.149	2.828	25
Neurotransmitter release cycle	2.14	0.225	1	9
Factors Promoting Cardiogenesis in Vertebrates	2.14	0.152	1.706	23
Neuregulin Signaling	2.11	0.161	3	19
Glutamine Degradation I	2.1	1	-	2
eNOS Signaling	2.09	0.149	0.775	24
Creatine metabolism	2.08	0.4	0	4
Glucose and Glucose-1-phosphate Degradation	2.08	0.4	-	4

PIP3 activates AKT signaling	2.05	0.152	2.558	22
Gap Junction Signaling	2.05	0.128	0.156	43
Class B/2 (Secretin family receptors)	2.04	0.168	1	16
Role of MAPK Signaling in Inhibiting the Pathogenesis of Influenza	2.04	0.177	1.387	14
Autism Signaling Pathway	2	0.129	2.271	40
CGAS-STING Signaling Pathway	1.99	0.152	3.13	21
Extrinsic Prothrombin Activation Pathway	1.98	0.312	-	5
G alpha (q) signalling events	1.96	0.144	3	25
Lipid particle organization	1.95	0.5	-	3
HGF Signaling	1.94	0.153	2.53	20
CDK5 Signaling	1.93	0.16	-0.333	17
Striated Muscle Contraction	1.92	0.222	2.121	8
IL-13 Signaling Pathway	1.92	0.154	0.728	19
BEX2 Signaling Pathway	1.9	0.171	1.155	14
ERK5 Signaling	1.9	0.176	3.317	13
Nuclear Cytoskeleton Signaling Pathway	1.9	0.136	3.651	30
Th1 and Th2 Activation Pathway	1.9	0.142	-	25
Neutrophil degranulation	1.89	0.119	4.371	57
FAT10 Cancer Signaling Pathway	1.89	0.196	2.333	10
Azathioprine ADME	1.89	0.261	0	6
NFE2L2 regulating anti-oxidant/detoxification enzymes	1.89	0.261	0.816	6
L1CAM interactions	1.89	0.153	3.441	19
Microautophagy Signaling Pathway	1.88	0.145	1	23
Sleep NREM Signaling Pathway	1.87	0.155	0.5	18
Metabolism of folate and pterines	1.87	0.294	-0.447	5
cAMP-mediated signaling	1.86	0.133	-0.577	32
Estrogen-Dependent Breast Cancer Signaling	1.86	0.169	1.633	14
CD40 Signaling	1.86	0.179	2.53	12
Tryptophan Degradation X (Mammalian, via Tryptamine)	1.85	0.233	-0.816	7
Pancreatic Adenocarcinoma Signaling	1.85	0.152	2.887	19
Gα12/13 Signaling	1.84	0.149	3.153	20
Amyotrophic Lateral Sclerosis Signaling	1.83	0.154	1.155	18
Cerebral Malformation Signaling Pathway	1.83	0.154	2.828	18
Cholecystokinin/Gastrin-mediated Signaling	1.83	0.154	2.5	18

Hepatitis B Chronic Liver Pathogenesis Signaling Pathway	1.81	0.138	3.922	26
PPARα/RXRα Activation	1.79	0.136	-1.342	27
ERBB Signaling	1.78	0.161	3.207	15
Metabolism of steroid hormones	1.78	0.211	0	8
Reversible hydration of carbon dioxide	1.77	0.333	0	4
Specification of primordial germ cells	1.77	0.333	2	4
Apelin Endothelial Signaling Pathway	1.76	0.145	2.84	21
Sensory processing of sound by inner hair cells of the cochlea	1.76	0.174	0.577	12
ROBO SLIT Signaling Pathway	1.75	0.148	-0.471	19
Retinol Biosynthesis	1.72	0.185	-1.633	10
Potassium Channels	1.72	0.155	0.5	16
Multiple Sclerosis Signaling Pathway	1.72	0.132	3.157	29
Role of PKR in Interferon Induction and Antiviral Response	1.71	0.145	2.324	20
Semaphorin Neuronal Repulsive Signaling Pathway	1.7	0.143	0.471	21
Surfactant metabolism	1.7	0.219	0.378	7
Gustation Pathway	1.69	0.133	2.502	28
Neuroprotective Role of THOP1 in Alzheimer's Disease	1.68	0.146	1.155	19
Glucocorticoid Receptor Signaling	1.67	0.113	-	68
Uracil Degradation II (Reductive)	1.65	0.667	-	2
Serine Biosynthesis	1.65	0.667	-	2
Thyronamine and Iodothyronamine Metabolism	1.65	0.667	-	2
Thymine Degradation	1.65	0.667	-	2
Thyroid Hormone Metabolism I (via Deiodination)	1.65	0.667	-	2
Dopamine Degradation	1.65	0.2	-0.378	8
Cholesterol Biosynthesis I	1.64	0.308	-2	4
Cholesterol Biosynthesis II (via 24,25-dihydrolanosterol)	1.64	0.308	-2	4
Cholesterol Biosynthesis III (via Desmosterol)	1.64	0.308	-2	4
TNFR2 Signaling	1.63	0.212	2	7
IL-33 Signaling Pathway	1.63	0.133	3.838	26
Metabolism of porphyrins	1.63	0.231	-2.449	6
Thrombin Signaling	1.62	0.13	2.065	29
CD27 Signaling in Lymphocytes	1.62	0.179	2.121	10
Ovarian Cancer Signaling	1.62	0.138	1.89	22
Sleep REM Signaling Pathway	1.61	0.151	-1.5	16

Myelination Signaling Pathway	1.61	0.122	4.427	40
DAG and IP3 signaling	1.59	0.195	1.134	8
Chronic Myeloid Leukemia Signaling	1.59	0.125	3.43	35
Opioid Signaling Pathway	1.59	0.125	0.756	35
IL-1 Signaling	1.59	0.153	1.414	15
PTEN Signaling	1.59	0.139	-2.496	21
PFKFB4 Signaling Pathway	1.57	0.184	0.378	9
Role of JAK2 in Hormone-like Cytokine Signaling	1.57	0.169	0.333	11
Tuberculosis Latent Signaling Pathway	1.57	0.156	0	14
Folate Signaling Pathway	1.57	0.175	1.667	10
Apelin Liver Signaling Pathway	1.56	0.25	2.236	5
Type II Diabetes Mellitus Signaling	1.56	0.138	1	21
Formation of intermediate mesoderm	1.56	0.375	-	3
PPAR Signaling	1.55	0.148	-2.673	16
April Mediated Signaling	1.53	0.19	2.646	8
Glutamate and glutamine metabolism	1.52	0.286	0	4
Triacylglycerol Biosynthesis	1.52	0.172	-0.707	10
Tight Junction Signaling	1.52	0.133	-	24
Erythropoietin Signaling Pathway	1.52	0.133	-0.426	24
Human Embryonic Stem Cell Pluripotency	1.51	0.13	2.746	26
B Cell Activating Factor Signaling	1.48	0.186	2.449	8
Sulfur amino acid metabolism	1.48	0.214	-1.633	6
RHO GTPases activate PKNs	1.48	0.214	2.449	6
CDP-diacylglycerol Biosynthesis I	1.48	0.214	1	6
Cyclophilin Signaling Pathway	1.47	0.124	3.053	31
GPCR-Mediated Integration of Enteroendocrine Signaling Exemplified by an L Cell	1.46	0.158	-0.577	12
NOD1/2 Signaling Pathway	1.45	0.13	3.838	25
GNRH Signaling	1.45	0.13	1.886	25
SNARE Signaling Pathway	1.44	0.138	2.065	19
Virus Entry via Endocytic Pathways	1.44	0.142	3.5	17
MSP-RON Signaling in Macrophages Pathway	1.44	0.142	1.291	17
Activation of NMDA receptors and postsynaptic events	1.44	0.153	0.302	13
Netrin Signaling	1.44	0.131	2.837	23
GADD45 Signaling	1.43	0.167	-0.632	10

YAP1- and WWTR1 (TAZ)-stimulated gene expression	1.42	0.267	2	4
Peroxisomal lipid metabolism	1.41	0.207	-2.449	6
TNFs bind their physiological receptors	1.41	0.207	1.633	6
EGR2 and SOX10-mediated initiation of Schwann cell myelination	1.41	0.207	0.816	6
Serine biosynthesis	1.41	0.333	-	3
Histidine Degradation III	1.41	0.333	-	3
GDP-glucose Biosynthesis	1.41	0.333	-	3
Epithelial Adherens Junction Signaling	1.4	0.133	0.894	21
Fatty Acid α-oxidation	1.4	0.227	-0.447	5
Senescence Pathway	1.39	0.12	3.286	36
Thyroid Cancer Signaling	1.38	0.154	2.309	12
Retinoate Biosynthesis II	1.38	0.5	-	2
D-glucuronate Degradation I	1.38	0.5	-	2
L-cysteine Degradation I	1.38	0.5	-	2
Signaling by EGFR	1.38	0.17	2.333	9
Estrogen Receptor Signaling	1.37	0.114	3.479	47
Regulation of CDH11 Expression and Function	1.35	0.2	2.449	6
Glutathione Redox Reactions I	1.35	0.2	1	6
Phosphatidylglycerol Biosynthesis II (Non-plastidic)	1.35	0.2	1	6
Thyroid Hormone Metabolism II (via Conjugation and/or Degradation)	1.35	0.161	0	10
Acetylcholine Receptor Signaling Pathway	1.34	0.126	1.225	25
Amyloid fiber formation	1.34	0.148	1.941	13
Death Receptor Signaling	1.33	0.144	1.155	14
TGF-β Signaling	1.33	0.144	0.632	14
Triglyceride metabolism	1.33	0.184	-2.646	7
MIF Regulation of Innate Immunity	1.33	0.174	1.89	8
IL-23 Signaling Pathway	1.33	0.174	0.378	8
Histamine Degradation	1.32	0.217	-1	5
Th1 Pathway	1.3	0.136	1.698	17

Supplemental Figures

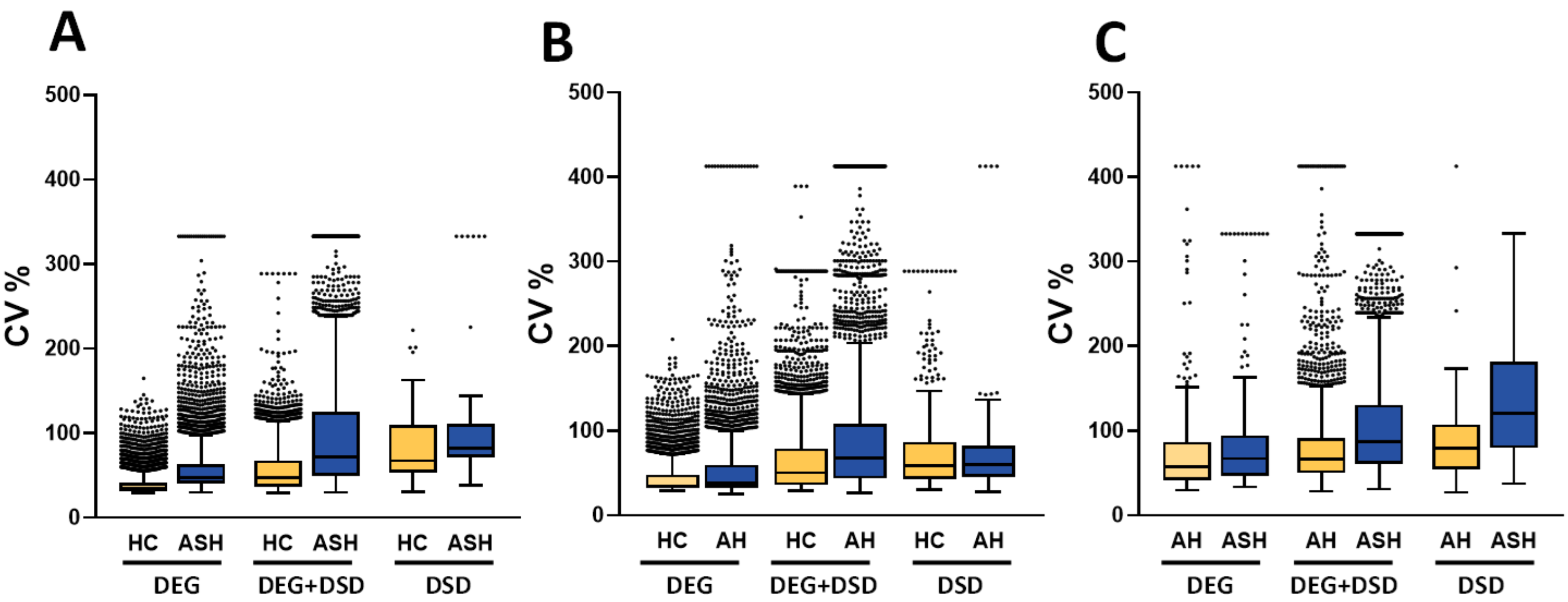

Figure S1

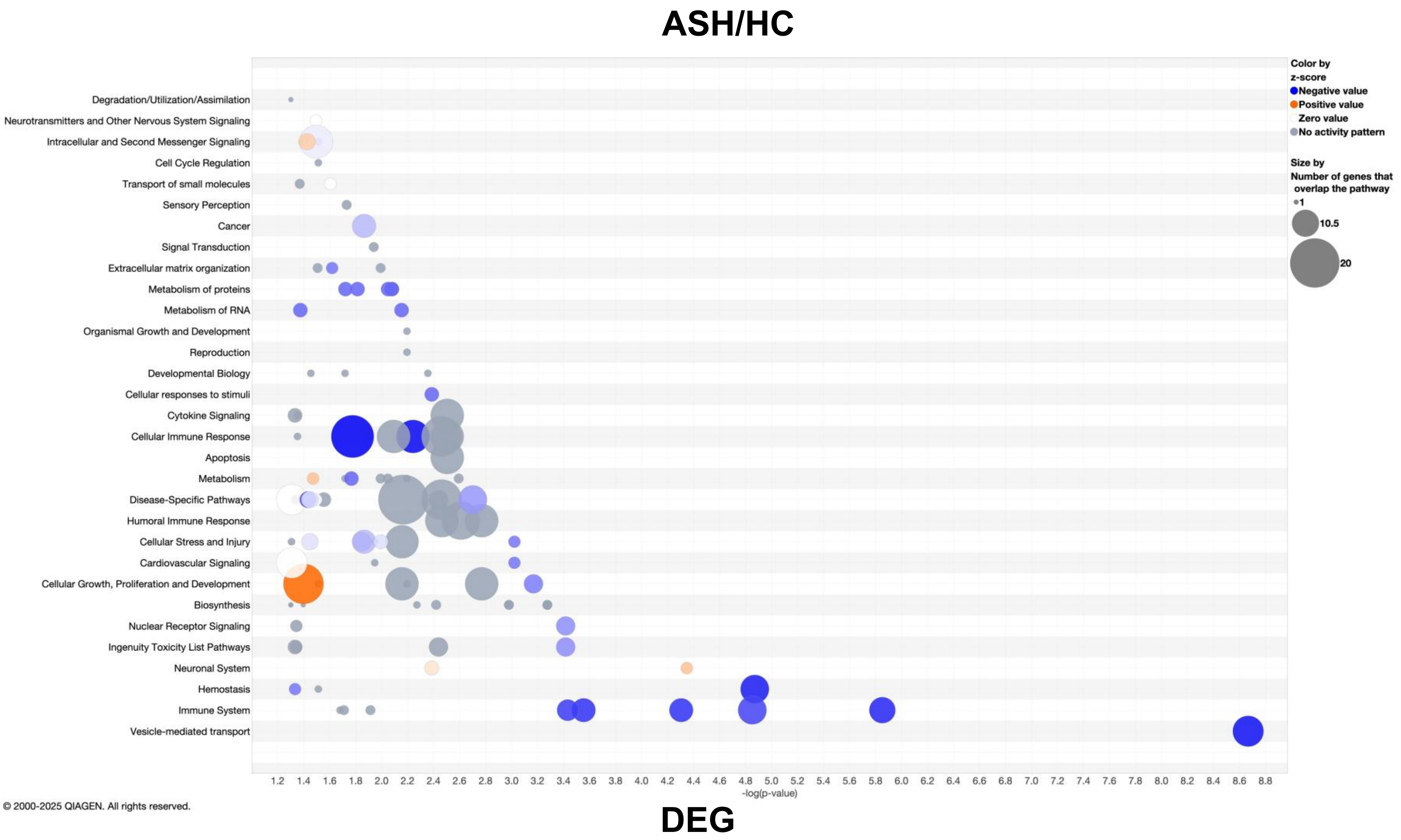

© 2000-2025 QIAGEN. All rights reserved.

Figure S2

ASH/HC

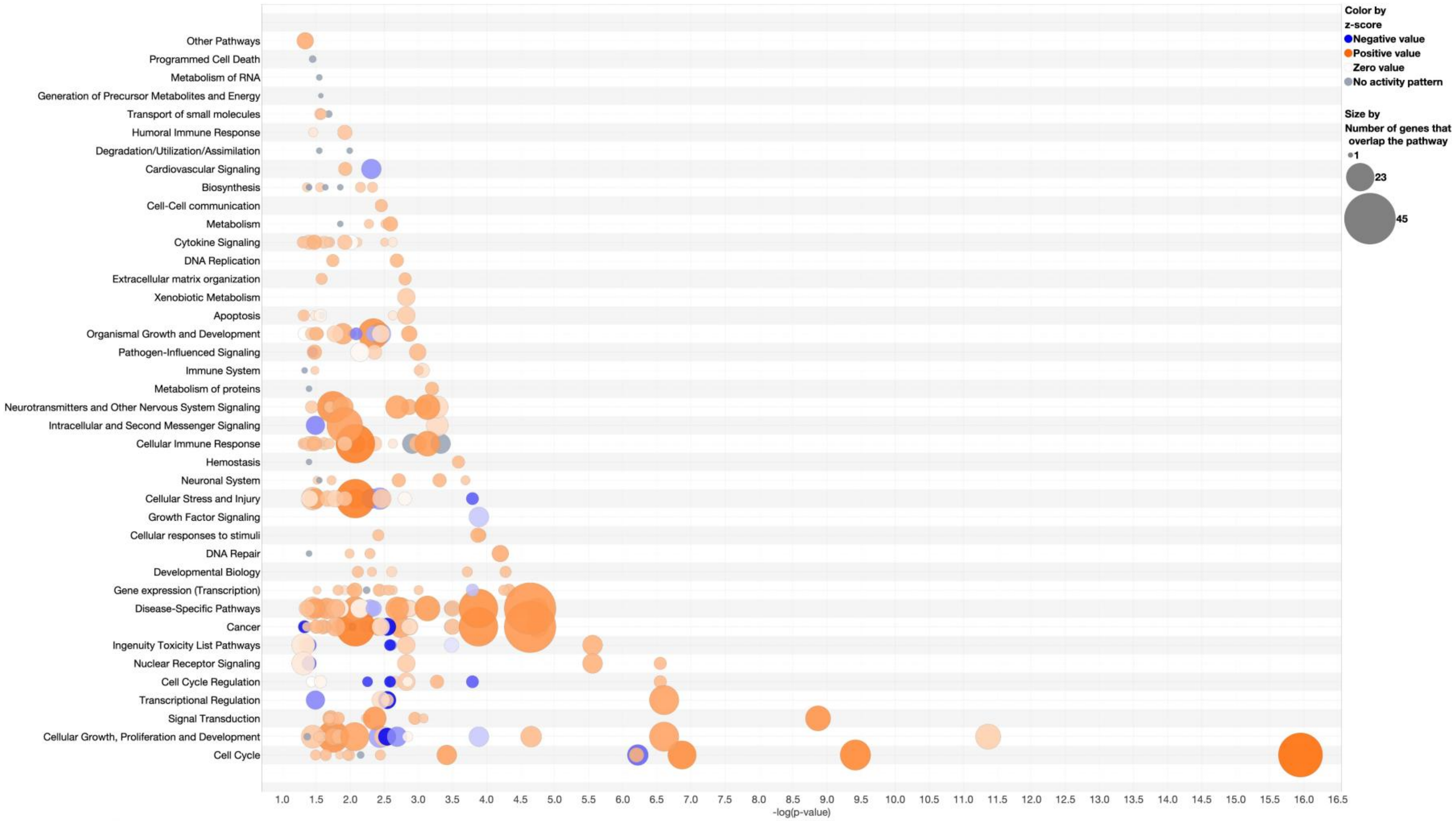

© 2000-2025 QIAGEN. All rights reserved.

DSD

Figure S2

ASH/HC

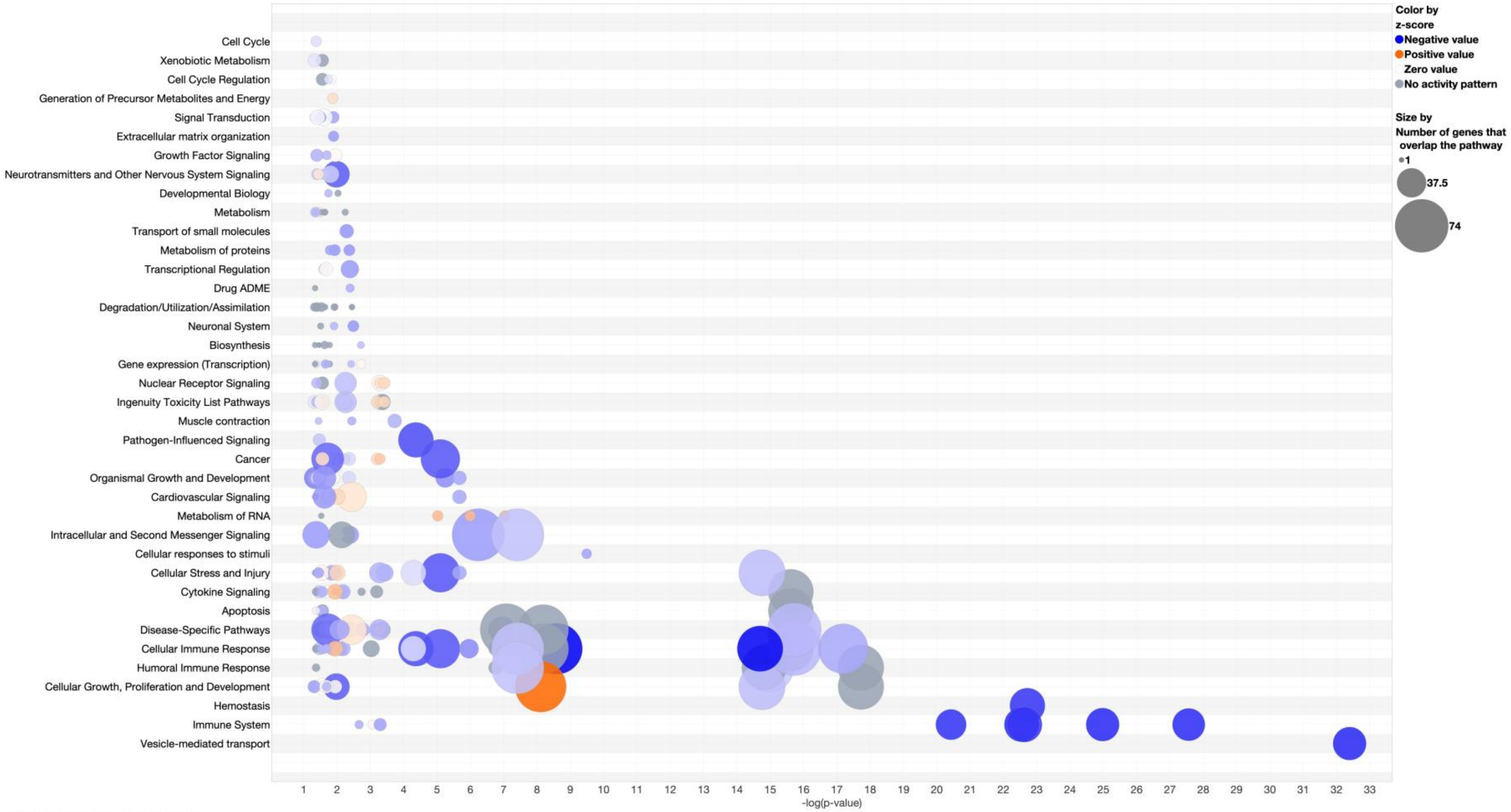

© 2000-2025 QIAGEN. All rights reserved.

DEG and DSD (Common)

Figure S2

AH/HC

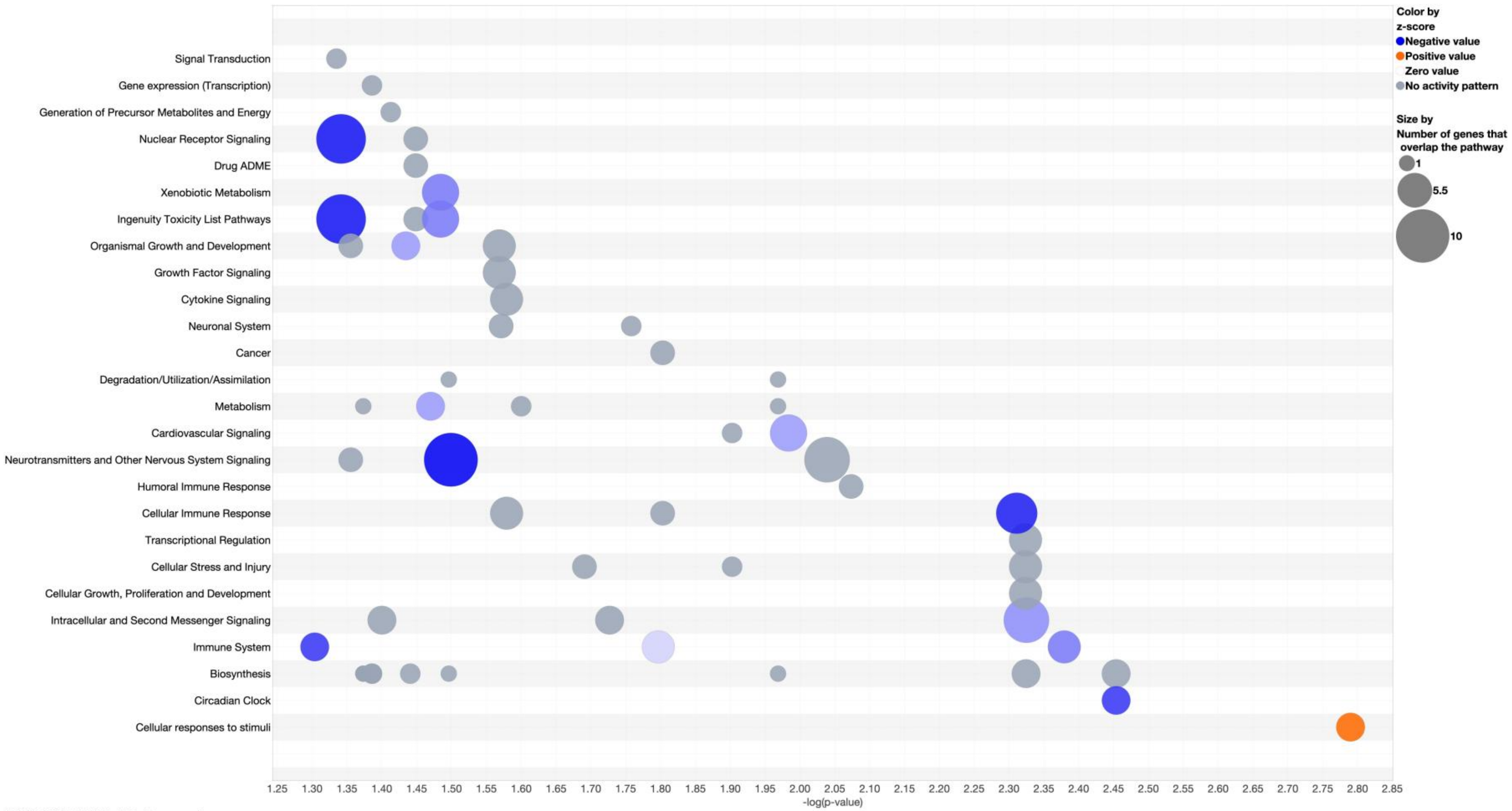

© 2000-2025 QIAGEN. All rights reserved.

DEG

Figure S3

AH/HC

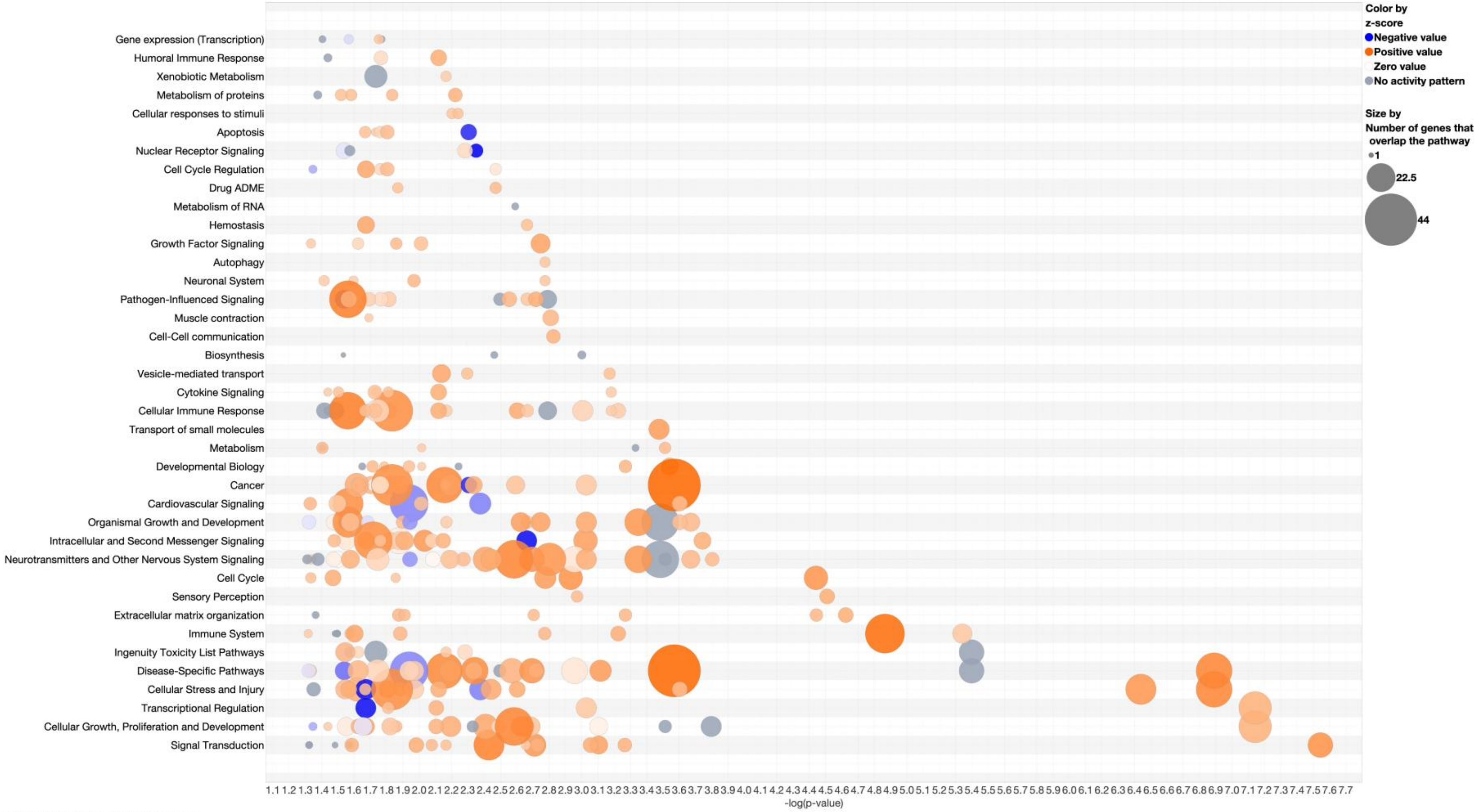

© 2000-2025 QIAGEN. All rights reserved.

DSD

Figure S3

AH/HC

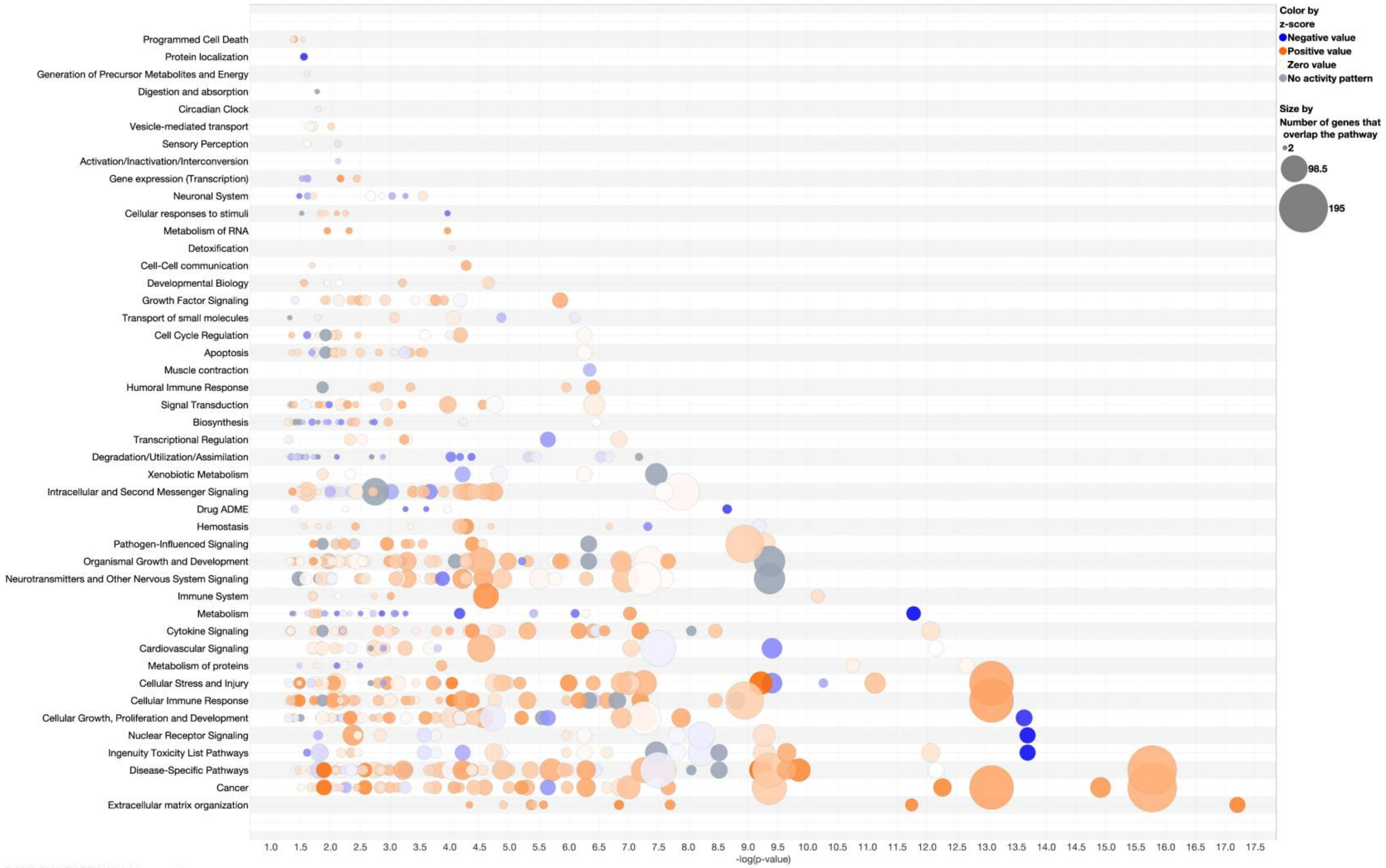

© 2000-2025 QIAGEN. All rights reserved.

DEG and DSD (Common)

Figure S3

Supplemental Figure legends

Figure S1(A-C): Variability represented by the percentage of CV (coefficient of variation, that is standard deviation divided by mean) of three abundance distributions ASH vs HC (S1), AH vs HC (S2) and AH vs ASH (S3) for genes exclusive to DEG and DSD and common and shared by both DEG and DSD analysis.

Figure S2, S3: Canonical Pathway bubble chart. Canonical Pathway scores plotted as pathway category based on gene expression level for the DEG and DSD exclusive and common pathways in the ASH vs. HC and AH vs. HC group. The log of the p-value for each pathway is plotted on the x-axis versus the pathway categories plotted along the y-axis. By default, the coloring relates to the pathway's z-score, and the bubble size relates to the number of dataset genes that overlap each pathway, as shown in the legend.
